# Times are changing but order matters: transferable prediction of small-molecule liquid chromatography retention times

**DOI:** 10.1038/s41592-026-03243-2

**Published:** 2026-10-01

**Authors:** Fleming Kretschmer, Eva-Maria Harrieder, Michael Witting, Sebastian Böcker

**Affiliations:** 1 Chair for Bioinformatics, Institute for Computer Science, Friedrich Schiller University Jena, Jena, Germany; 2 Research Unit Analytical BioGeoChemistry, Helmholtz Zentrum München, Neuherberg, Germany; 3 Metabolomics and Proteomics Core, Helmholtz Zentrum München, Neuherberg, Germany; 4 Chair of Analytical Food Chemistry, TU München, Freising, Germany; 5 Present Address: Labor Becker MVZ eGbR, Munich, Germany

**Keywords:** Machine learning, Metabolomics

## Abstract

Liquid chromatography is a predominant technology for the separation of small molecules. Hundreds of retention time prediction models have been published over the past decades, yet truly transferable prediction (requiring no training data from the target chromatographic system) remains an open challenge. Unfortunately, retention times may change massively, even for nominally identical chromatographic conditions. Retention order is considerably more conserved; but even retention order may change if chromatographic conditions vary. Here we present 2-step, a two-step method for the prediction of retention times in reversed-phase chromatography. In the first step, a machine-learning model predicts a retention order index, taking into account chromatographic conditions. In the second step, we map predicted indices to absolute retention times. Disentangling these two tasks finally enables transferable retention time prediction across chromatographic conditions and compound classes, without requiring any target-system training data. Our 2-step method outperforms existing methods that were trained on the target dataset. Finally, we systematically study what chromatographic conditions result in notable changes of retention order.

## Abstract (web summary)

A method for transferable retention time prediction across chromatographic conditions and compound classes is presented. The approach makes use of a two-step strategy that first predicts a condition-aware retention order index followed by mapping it to absolute retention times.

## Main

Liquid chromatography (LC) allows us to separate a complex chemical mixture into its individual components. It is frequently employed in the life sciences to separate molecules of biological interest, such as peptides, metabolites or lipids. Retention time is the time a particular molecule requires to pass through the LC column and is dependent on the molecule structure and the applied chromatographic conditions; clearly, this value carries information about the molecule’s identity (for example, polarity based on the separation mode). Consequently, using machine learning to predict retention time from the structure of a molecule is one of the most investigated problems in the field of quantitative structure-property relationships (QSPR)^1^. Whereas predicting retention times is relatively straightforward for polymeric biomolecules such as peptides^2,3^ and oligonucleotides^4^, it turned out to be an extremely challenging problem for so-called small molecules with mass below 1,500 Da. We will concentrate on small molecules of biological interest (‘biomolecules’) such as primary and secondary metabolites, drugs and drug degradation products, and toxins.

Despite four decades of development^1,5–7^ and many hundreds of publications, retention time prediction for small molecules is still far from being in everyday use. The main problem is that retention time is not a property of a compound alone but rather, a combination of the employed chromatographic column, eluents and their composition, the compound itself, and further used LC equipment. Usually, a publication on retention time prediction proceeds by first measuring a small dataset of at most a few hundred compounds on one particular fixed experimental platform, then training and evaluating a machine-learning model using these data. The resulting models cannot transfer predictions to novel chromatographic conditions or molecules that are structurally very different from those used in training and evaluation. One method that can predict retention times for different chromatographic conditions is MultiConditionRT^8^, which takes into account eluent composition for its predictions, but does not consider multiple chromatographic columns beyond separation mode.

The currently best-performing methods for retention time prediction use transfer learning^9–17^. Here, a machine-learning model is first pretrained on a large dataset with arbitrary chromatographic conditions, then fine-tuned on a smaller target dataset with the chromatographic conditions of interest. Notably, such models cannot make predictions for an arbitrary chromatographic setup; rather, we have to measure hundreds of authentic standards on our target system, then fine-tune (train) the model on these data before predicting any retention times. The resulting model cannot be used for any other chromatographic systems, even those that are nominally identical.

Retention indices enable the normalization of differences in chromatographic conditions using a set of standards. This strategy is commonly used in gas chromatography, and has recently been suggested for LC^18–20^. Unfortunately, elution order may change in different chromatographic conditions^21^, rendering a universal normalization by standards impossible. Retention projection approaches map retention times between different LC gradients, but have restrictions with regard to the employed column or mobile phase^21,22^. Clearly, we may choose to predict a retention index instead of retention time^22,23^. For this, index standards must have been measured for all LC runs in the training data, as well as for the target chromatographic condition^24^, severely limiting the available training data.

**Extended Data Fig. 1:**
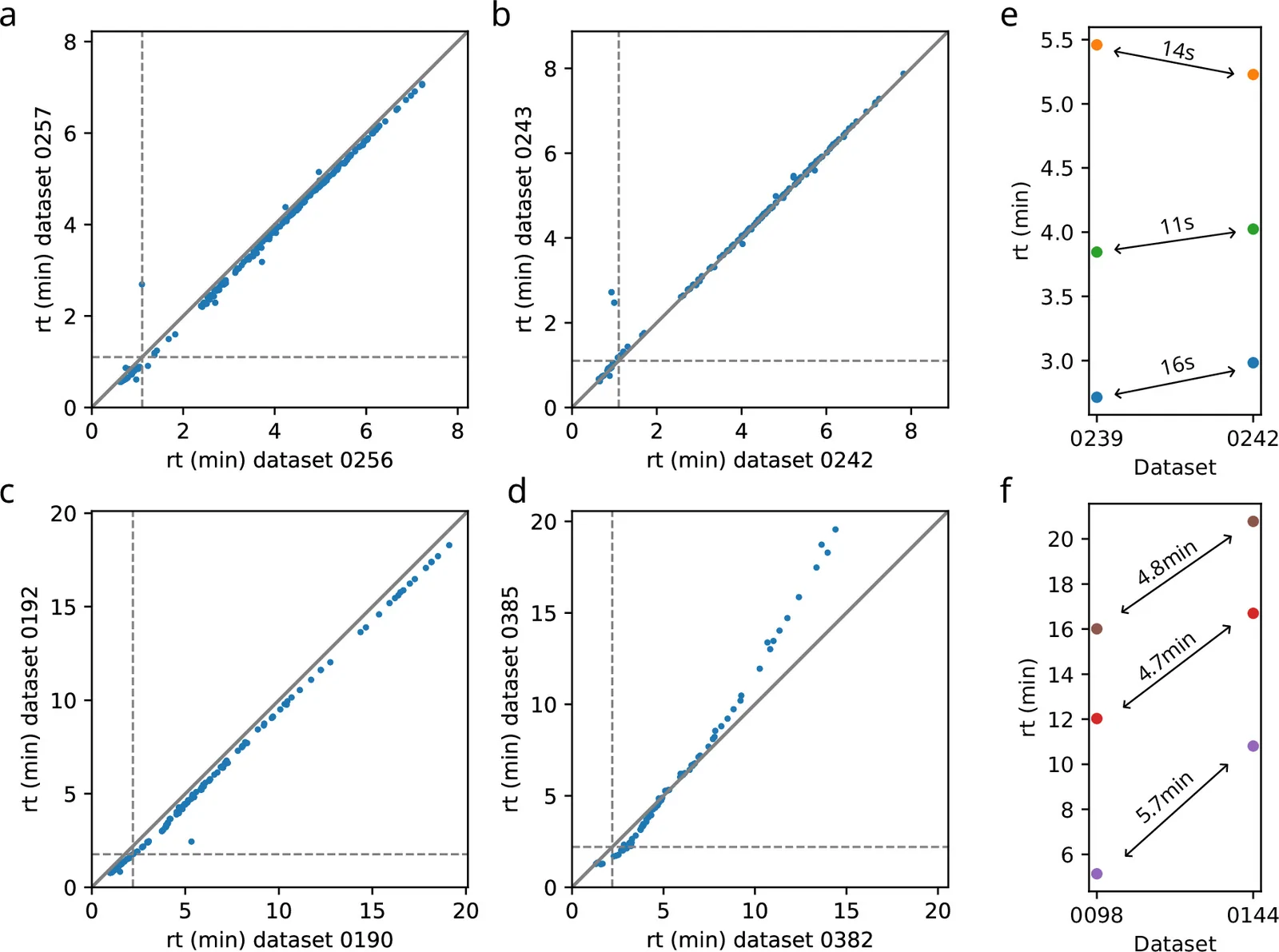
Retention time deviations for nominally identical or highly similar setups. Shown are pairs of datasets with nominally identical (**a**,**b**) or very similar (**c**: differing slightly in flow rate, **d**: differing in gradient and flow rate) chromatographic conditions but deviating retention times. The thresholds for the void volumes are displayed as dashed lines, the solid line through the origin corresponds to exact matches in retention times. Examples of single compounds with substantially varying retention times between datasets of nominally identical (**e**) and very similar (**f**, differing in gradient and flow rate) chromatographic conditions are also shown; note the different magnitude of retention time variations between the two examples.

Retention order is known to be much better conserved than retention time^21,25^: Using a longer capillary between the exit of the column and the entrance of the mass spectrometer will cause a shift of retention times for all compounds, but does not change their elution/retention order (Extended Data Fig. 1ef). Consequently, some computational methods focus on retention order: PredRet^25^ computes a nonlinear mapping of retention times between two datasets (see also refs. ^24,26^). Bach et al.^27^ suggested predicting retention order instead of retention time (refs. ^28,29^). Bach et al.^27^ also pioneered the idea of predicting a retention order index (ROI), allowing a machine-learning model to ‘create’ an index that best fits the data. This differs from the computational task of predicting a retention index. There, one would decide for one particular retention index type (based on specific chemical indexing substances) upfront, then transform all measured retention times into retention index values, and finally, train a machine-learning model that predicts exactly this retention index type. Of note, predicting an ROI does not require the measurement of any authentic standards for the training data or the target system. Again, all methods implicitly assume that retention order does not change between datasets and chromatographic conditions.

In metabolomics, natural products research, environmental research and numerous related areas from analytical chemistry and life science, LC is usually combined with mass spectrometry (LC–MS). This combination has been utilized in millions of studies and can detect thousands of compounds in complex biological samples. Tandem mass spectrometry (MS/MS) is used to annotate small molecules. Notably, no generally agreed chromatographic separation methods have been established in the metabolomics or other life science communities^30^: Metabolomics laboratories often specialize in a specific topic or organism (for example, human gut or plants), and methods are then optimized for the specific matrix and sample type. LC vendors offer various instruments with different capabilities, and thousands of different column types. Consequently, with the exception of searching in-house MS/MS spectral libraries measured on the same chromatographic system, retention times are usually ignored for compound annotation. Only at the last stage, when a handful of putatively annotated features are compared against authentic standards, retention times are considered.

## Results

Throughout this manuscript, we concentrate on reversed-phase chromatography, as hydrophilic interaction chromatography (HILIC) columns are substantially more diverse. We present a method for transferable prediction of retention times, meaning that our method can do predictions for an arbitrary chromatographic setup, without the need of training data from that setup. Our method proceeds in two steps: In the first step, we use a machine-learning model to predict retention order indices. This model allows us to predict retention order indices for an arbitrary chromatographic setup; it outperforms all models that ignore chromatographic conditions. Notably, the model does not require any training data from the target system, comparable to the pretrain phase of pretrain/fine-tune models. Clearly, the target dataset used for evaluation is never part of the training data. In the second step, we map predicted retention order indices to retention times using a low-order polynomial. For doing so, about 30 compounds in the target dataset must be putatively identified with high confidence, such as by spectral library search. Our method allows us to predict retention times for any chromatographic setup, without the need of measuring references or training a model for that setup. In our evaluation, our method outperforms two state-of-the-art methods for retention time prediction when evaluated using a realistic split of the target dataset. Notably, these methods follow the pretrain/fine-tune paradigm and cannot do transferable predictions, but rather had to be trained using data from the target system.

Notably, retention time constitutes orthogonal information to the MS/MS of a compound. Such orthogonal information is required to reach a level 1 identification^31,32^ but at present, restricts library searches to in-house libraries, where retention times were recorded for the target chromatographic system. Here, transferable retention time prediction allows us to reach ‘almost level 1’ identifications when searching in more comprehensive public spectral libraries. Other areas of application include improving the quality of spectral library search, integration with in silico methods that search small-molecule MS/MS in structure databases, resolving stereochemistry of annotated compounds, optimizing chromatographic parameters and more (Supplementary Note 1). Of note, transferable prediction of retention times has fundamental advantages over direct prediction and pretrain/fine-tune methods. In particular, pretrain/fine-tune methods cannot make predictions unless hundreds of reference compounds were measured on the target chromatographic system (Supplementary Note 2). Notably, the performance of our 2-step approach improves when more datasets become available, whereas pretrain/fine-tune methods do not improve in performance.

What metadata about chromatographic conditions do we need to preserve for posterity, to enable the re-analysis and reuse of retention times recorded in a dataset? As mentioned, retention times may vary substantially even for nominally identical chromatographic conditions. Variations may be due to different void volumes, gradient delay volumes and related parameters of the LC systems, which in turn depend on the used capillaries and numerous other parts of the LC, or alterations of the column due to repeated use. Clearly, such variations are the rule and not the exception. Retention order between nominally similar systems remains essentially unchanged; yet, it can change massively for deviating chromatographic parameters. We identified a minimum set of basic parameters (column, mobile phase, gradient, temperature and flow rate) apparently sufficient to determine the retention order of a chromatographic system.

### Predicting retention order indices

We first study the problem of predicting an ROI^27^. Given a compound and chromatographic conditions of an experiment, we predict a real-valued number (the ROI) as follows: if compound A elutes before compound B under chromatographic conditions *Z*, then retention order indices *x* of A,*Z* and *y* of B,*Z* should satisfy *x* < *y*. Predicting a ROI is conceptually similar to learning-to-rank methods from information retrieval^33^; different from there, loss and evaluation must not concentrate on top predictions but rather, consider all compounds equally. Predicting ROI instead of retention order has several conceptual advantages. A model for predicting ROI can also predict retention order, whereas the reverse is not true. Most notably, a model predicting ROI can make a prediction for a single compound, instead of a pair of compounds. In the next section, we show how this enables transferable prediction of retention times.

**Extended Data Fig. 2:**
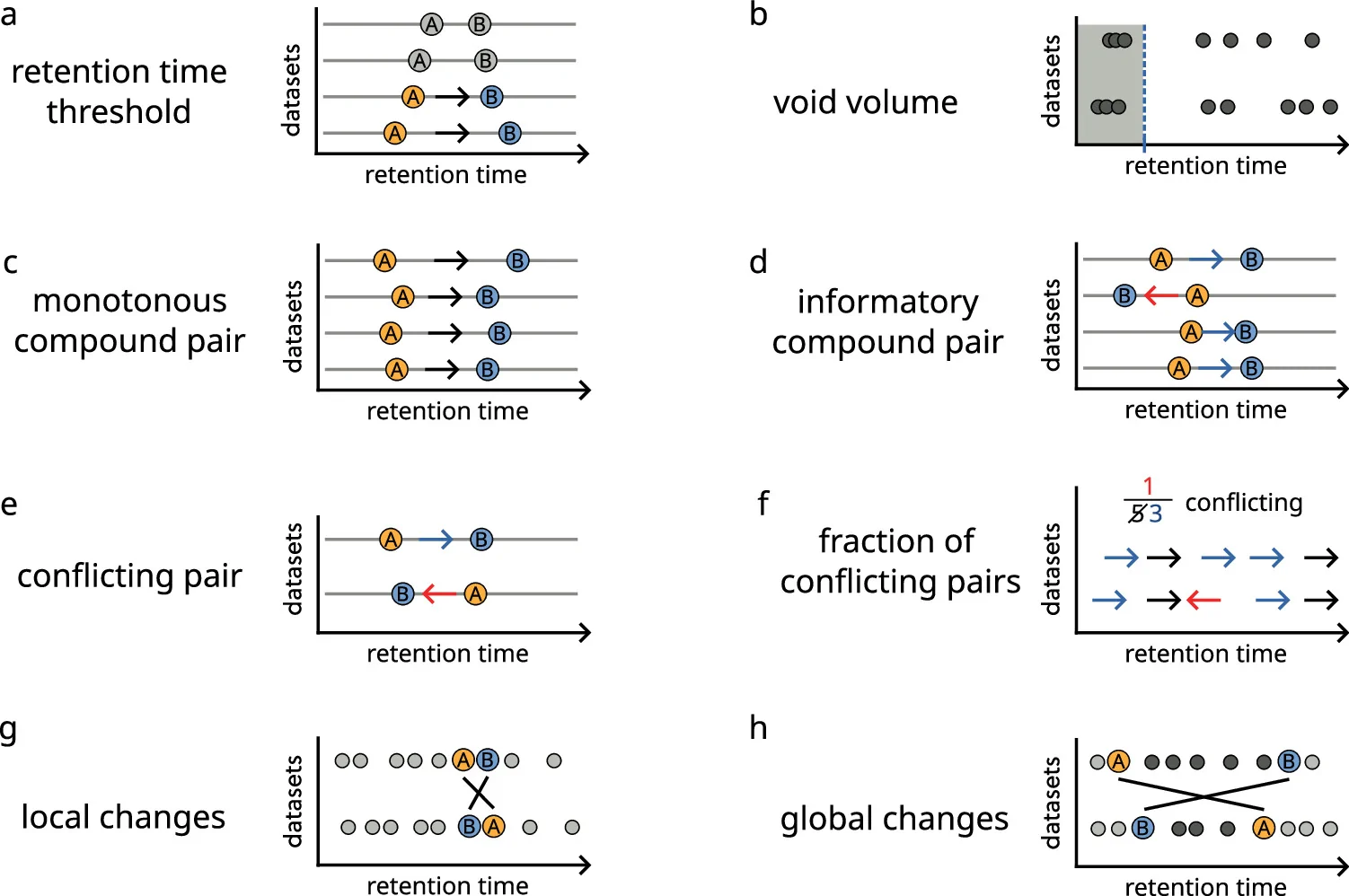
Illustrations of the most important retention order-related concepts. (**a**) We consider only compound pairs with retention time difference above a threshold; compounds with almost identical retention times may elute in varying order for a number of reasons, including inaccurate picking of the apex from the extracted ion chromatogram. (**b**) We do not consider compound pairs eluting in the void volume, as the order in the void volume is mostly random. We *do consider* compound pairs where only one of the compounds is in the void volume. (**c**) A monotonous compound pair has the same order in all datasets; (**d**) an informatory pair has deviating order for at least one dataset. (**e**) A conflicting compound pair consists of two compounds that have different order in two datasets. (**f**) When computing the fraction of of conflicting pairs, we ignore monotonous pairs, pairs in the void volume, and pairs below the threshold. (**g**,**h**) Both local and global changes of retention order contribute to conflicting pairs.

For evaluations and statistics, we only consider compound pairs with retention time differences above a certain threshold; compounds with almost identical retention times may elute in varying order for a number of reasons, including inaccurate picking of the apex from the extracted ion chromatogram. Throughout this paper, we use a threshold of 10 s unless mentioned otherwise. Furthermore, it is not reasonable to compare the retention times or order of two compounds both eluting in the void volume (Extended Data Fig. 2).

For training and evaluation, we used data from RepoRT, a repository for retention time data plus metadata on chromatographic conditions^34^. RepoRT contains thousands of unique compounds measured on tens of chromatographic columns using varying eluents, flow rates, and temperatures. Following dataset curation (Methods), 171 reversed-phase datasets from RepoRT remained to train and evaluate our models. On average, 22.1% of entries elute within the void volume of their dataset. We used a chromatographic condition-disjoint fivefold cross-validation, where the method is evaluated exclusively on datasets and chromatographic conditions that were absent from the training data. We evaluated models using the fraction of compound pairs for which the model predicts the (in)correct order. As models often reach accuracy beyond 90%, we instead report the error rate (one minus accuracy). To avoid large datasets dominating our evaluation statistics, we computed error rates for each dataset individually and then obtained an average over all datasets.

**Fig. 1:**
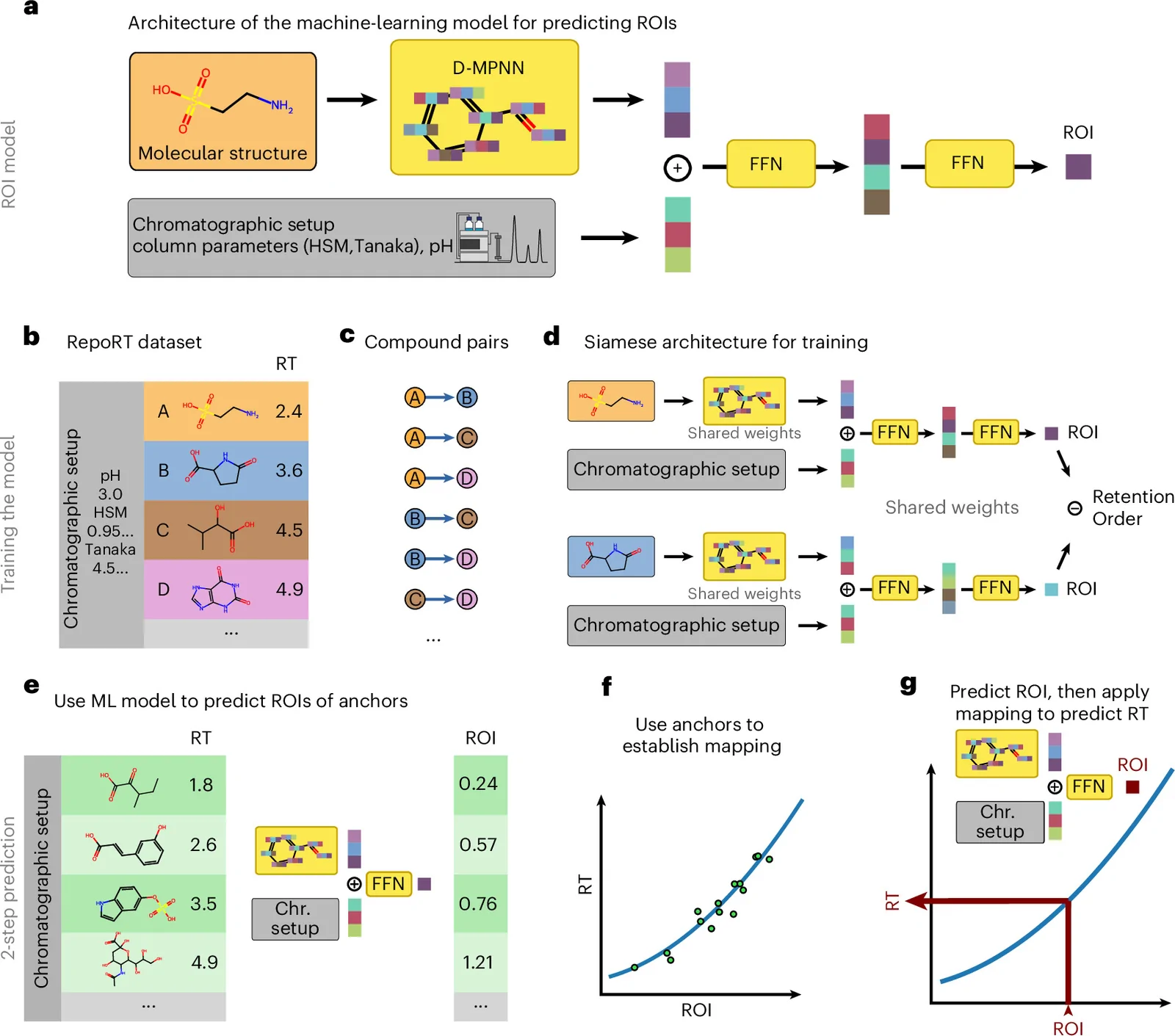
Workflow of the 2-step method. **a**, The machine-learning model inputs a molecular structure and chromatographic condition parameters, and outputs a single number, the ROI. Compound structures are encoded using a D-MPNN, extra layers encode the chromatographic setup. FFN, feed-forward network. **b**, For training the ROI model, we use curated reversed-phase datasets from RepoRT. **c**, Each dataset is transformed into a set of compound pairs with retention order. **d**, We use compound pairs to train the ROI model using a Siamese architecture. In the loss function, we subtract the ROIs of the two compounds, and evaluate whether the implied order agrees with the training data. **e**, For predicting retention times in any target dataset, we establish a set of anchors using high-confidence annotations. ML, machine learning. **f**, We establish a mapping between ROI and retention time of the anchor compounds. **g**, Our 2-step method can now predict the retention time (RT) of an arbitrary compound structure, such as candidates from a molecular structure database. First, we apply the ML model to predict a ROI; second, we map the ROI to the predicted RT of the compound.

We trained a graph neural network (GNN) to predict ROI, using the chromatographic parameters as additional features (Fig. 1). Compounds are encoded as molecular graphs, with atoms being nodes and bonds being edges. Nodes are labeled by atom type, edges by bond type (single bond, double bond etc.) and hydrogens are encoded implicitly. Atom and bond features include information on stereochemistry but not three-dimensional (3D) structure. We use a directed message-passing neural network (D-MPNN) as the basis for our GNN, taking the molecular graph with its atom and bond features as input and producing an encoding of the molecule of size 512 (refs. ^35,36^). The chromatographic setup is encoded by a 13-dimensional vector, where mobile phase pH is a single real-valued number. Notably, chromatographic columns are encoded using a 12-dimensional vector, combining hydrophobic subtraction model (HSM) parameters^37^ and Tanaka parameters^38^. This allows the model to generalize between columns, and to make predictions for novel columns missing from the training data. Tanaka and HSM parameters quantify column properties such as hydrophobicity, steric selectivity and hydrogen bonding capacity. Chromatographic features are integrated into the neural network using two additional, independent layers both of size 256. After two further layers of size 256 and 64, the final output consists of a single neuron, the ROI of the compound. Solely for training the network, we use a Siamese architecture where the network is duplicated with identical weights. The Siamese network takes as input two compounds measured under identical chromatographic conditions in one dataset. In this Siamese training mode, the output consists of two neurons, being the ROIs of the two compounds. If the order of the compounds in the input dataset agrees with the ranks of the ROIs, this results in zero loss; otherwise, the loss depends on the retention time difference in the dataset via a sigmoid weight function, and the difference in predicted ROIs via the margin ranking loss function employed. The model has approximately 1 million parameters; it does not require us to encode chemical compounds beyond the graph representation of the molecular structure itself. Our chromatographic condition-aware model reaches an error rate of 4.89% (Extended Data Table 1).

For comparison, we also evaluated three models that do not (fully) use the information about chromatographic conditions. As a baseline, we evaluated the ranking support vector machine (RankSVM) from ref. ^27^, which ignores chromatographic conditions. Due to high training times, we uniformly selected 100 datasets from the RepoRT training data to train the RankSVM (Supplementary Table 1). The RankSVM reaches an error rate of 7.99%. Second, we trained a RankNet model^39^ using molecular descriptors as features, ignoring chromatographic conditions. This model reaches an error rate of 5.80%. Third, we trained and evaluated a GNN model without features for chromatographic conditions; the model uses basically the same state-of-the-art architecture and training procedure as the chromatographic condition-aware model but leaves out the part encoding the chromatographic setup (Fig. 1). As it has no information about the chromatographic setup, the model basically tries to predict the ‘majority vote’ among the datasets, as shown below. This model reaches an error rate of 5.61%. Fourth, we used a simple one-hot encoding for the column, instead of HSM and Tanaka parameters. Again, the model architecture and training are otherwise identical to the chromatographic condition-aware model. Clearly, the one-hot model cannot generalize to unseen columns; also, it cannot use information about column similarity in its predictions. The one-hot model reaches an error rate of 5.01%, demonstrating that our chromatographic condition-aware model is indeed already generalizing between datasets with similar columns. We repeated all of the above evaluations using a second, more challenging dataset split where the target dataset must not share column and similar pH with any training datasets. We observe similar trends, except that the one-hot encoding model performed worse than both the model ignoring chromatographic conditions and RankNet (Extended Data Table 1 provides the s.d. values).

**Extended Data Fig. 3:**
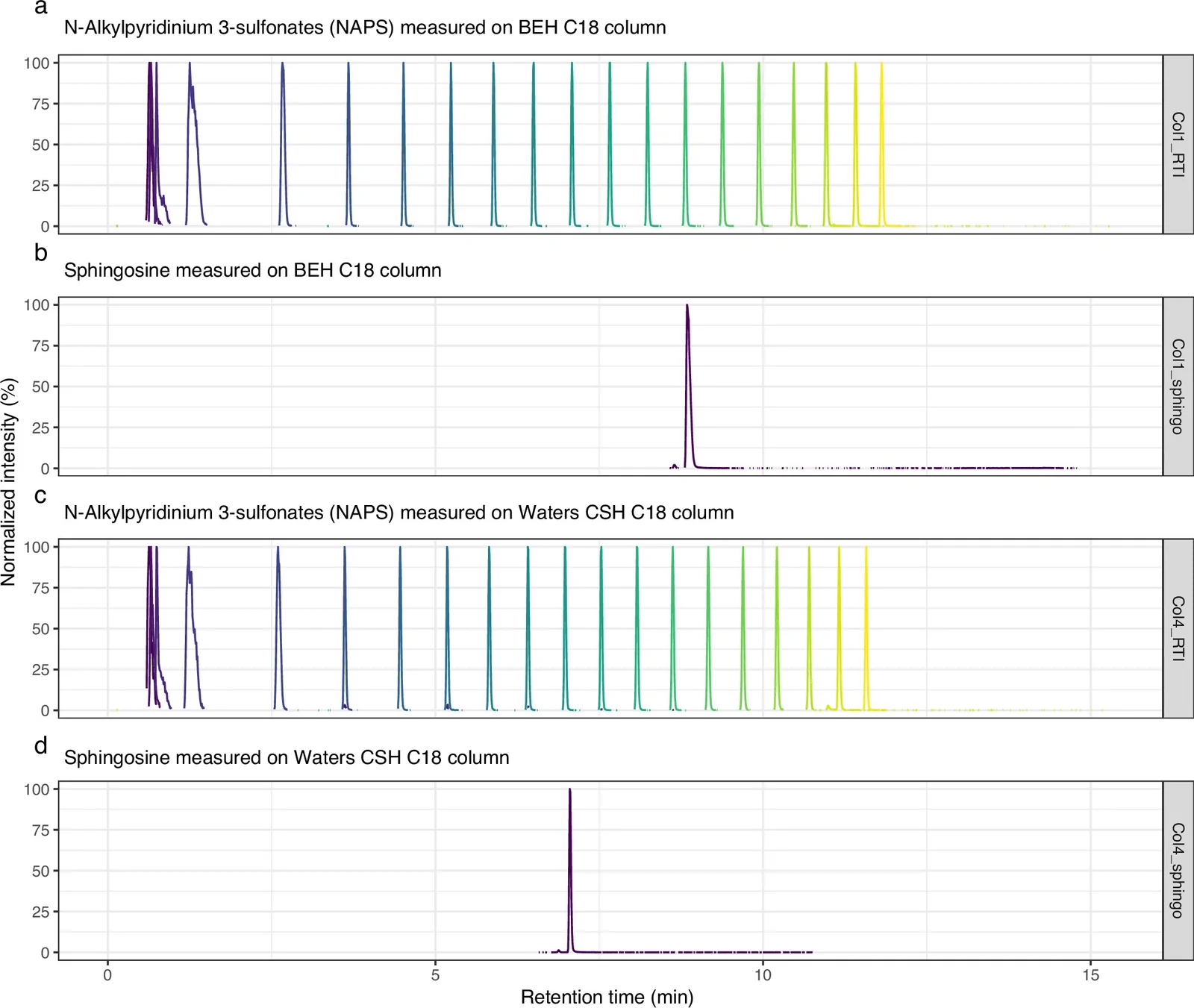
Monotonous versus informatory pairs. N-Alkylpyridinium 3-sulfonates (NAPS) (**a**,**c**) and sphingosine (**b**,**d**) were measured on a BEH C18 column (**a**,**b**) and a Waters CSH C18 column (**c**,**d**), with otherwise identical chromatographic parameters. Whereas retention times of NAPS are mostly unaffected and retention order remains unchanged, sphingosine shows a large shift in retention time, resulting in a change of retention order and three conflicting pairs between the two datasets (**a**,**b**) versus (**c**,**d**). Despite the massive change in sphingosine retention time, the fraction of conflicting pairs is only 2.2% (3 conflicting pairs out of 136 pairs, assuming 16 NAPS beyond the void volume). This example motivates the definition of monotonous and informatory pairs; we may assume that the NAPS molecules will not change order under any reasonable chromatographic conditions.

Our evaluation demonstrates that including chromatographic conditions indeed improves the performance of a model predicting retention order. It may seem counterintuitive that the performance of the model increases only slightly (error rates 4.89% versus 5.61% for the regular dataset split). This is in part an evaluation artifact. Certain compound pairs will never change order for any (reasonable) chromatographic conditions (such as the *N*-alkylpyridinium 3-sulfonate molecules shown in Extended Data Fig. 3). For an arbitrary pair of compounds, we generally cannot determine if they will not change order under any (reasonable) chromatographic conditions. Yet, we can check whether two compounds have identical order for any pair of datasets in the RepoRT repository. We define compound pair as monotonous if the same retention order is observed for all datasets; all other pairs are informatory (Extended Data Fig. 2). Numbers on monotonous and informatory pairs reported below are solely a snapshot of the current RepoRT repository. When new datasets are added, the number of informatory pairs in existing datasets can only increase, because a single dataset with deviating order is sufficient for that. We stress that predicting ROI would still be a sensible and important task if there were zero informatory pairs; in this case, we could simply ignore chromatographic conditions.

Clearly, including information about chromatographic conditions cannot improve a model’s performance for monotonous compound pairs. On average, there are 93.8% compound pairs per dataset in the training data where retention order does not change (Supplementary Figs. 1 and 2). For these compound pairs, the chromatographic condition-aware model cannot outperform its uninformed counterpart. These pairs clearly dominate the evaluation statistics. Informatory pairs make up 6.16% of all compound pairs; we stress that the seemingly small number is an artifact of considering pairs of compounds (Supplementary Note 3). Notably, 0.70% of compound pairs have changing order even when HSM/Tanaka parameters plus mobile phase pH are identical. Only models encoding chromatographic parameters beyond column and pH would be capable of predicting two different orders.

In the following analysis, we concentrate on the 5.46% compound pairs that are informatory, but do not change order for identical chromatographic parameters. Even for these pairs, retention order often only changes for few datasets. For every two compounds, we choose the order observed in the majority of datasets as the consensus retention order; we say that a compound pair is setup-characteristic if its retention order is different from the consensus retention order. On average, setup-characteristic compound pairs make up 1.26% of pairs per dataset. Setup-characteristic pairs are the apogee of evaluation statistics. Despite their relatively small number, they are the hardest to predict for a machine-learning model; they are of particular importance for certain applications (Supplementary Note 1); and their fraction will in all likelihood substantially increase when more datasets with ‘new’ compounds and ‘new’ chromatographic conditions are added to our training and evaluation data. Finally, recall that the small number is again an artifact of considering pairs of compounds (Supplementary Note 3).

**Fig. 2:**
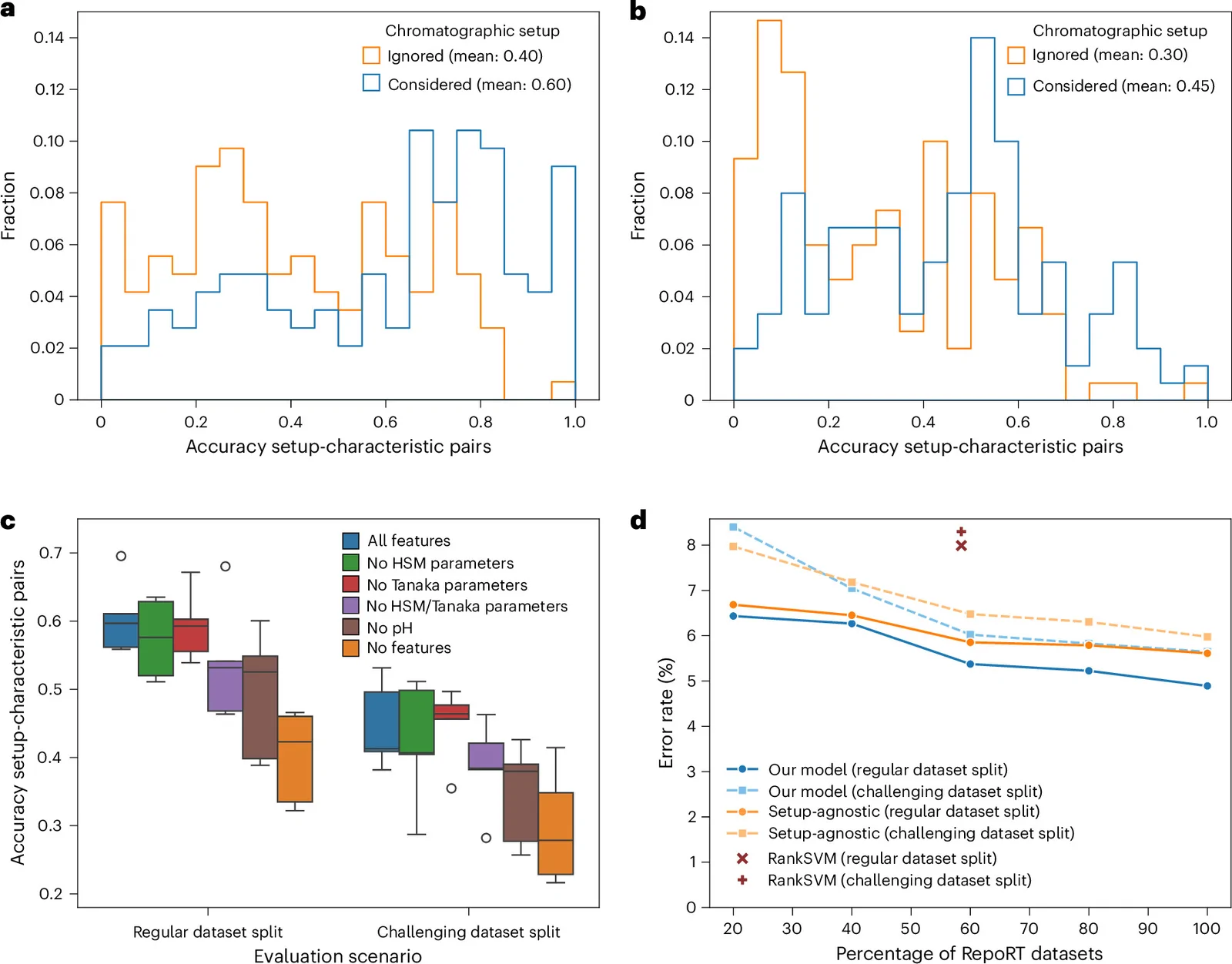
Detailed evaluation of ROI models. Training and test data were disjoint regarding (nominal) chromatographic setup (regular dataset split), or combinations of chromatographic column and estimated pH value (challenging dataset split). **a**,**b**, Comparison of the chromatographic condition-aware model and its uninformed counterpart, considering only setup-characteristic compound pairs. Distribution of accuracies (average per dataset) for the regular dataset split (**a**) and the challenging dataset split (**b**). **c**, Ablation studies for the chromatographic condition-aware ROI model. Accuracy for models excluding HSM parameters, Tanaka parameters, both, pH values, or all of these from the chromatographic features. Only setup-characteristic pairs are considered. The box plots show the distributions over five cross-validation folds per ablation study, depicting the first and third quartiles as the box limits with the median as center line. Whiskers extend to the smallest and largest value but no further than 1.5 × interquartile range from the hinges, with points falling outside shown explicitly. **d**, Error rate of ROI models trained only on fractions (20%, 40%, 60% and 80%) of the training data. All compound pairs are considered. Error rates of the RankSVM are shown for comparison; the RankSVM was trained on a reduced set of 100 (58.5%) RepoRT datasets. Source data

For setup-characteristic compound pairs, the performance of the model with and without knowledge of chromatographic parameters is indeed very different. For better differentiation, we use accuracy instead of error rate for all evaluations on setup-characteristic compound pairs. For these pairs, the average accuracy per dataset of the chromatographic condition-aware model increased from 40.1% to 60.5% for the regular dataset split, and from 29.7% to 44.6% for the challenging dataset split (Fig. 2a,b). Notably, this is not substantially better than flipping a coin; yet, flipping a coin would predict only 50% of the consensus pairs correctly, too. Any reasonable machine-learning model will try to learn the consensus retention order, and a good model that is setup-agnostic may actually predict none of the setup-characteristic pairs correctly.

Next, we investigated the influence of the different feature sets on model performance (Fig. 2c). While leaving out either HSM or Tanaka parameters did not result in a drop in accuracy, models with no features describing the chromatographic column show reduced accuracy, implying some redundancy between HSM and Tanaka parameters. Similarly, omitting pH resulted in a substantial decrease in accuracy. Unsurprisingly, the performance drops even more when no chromatographic features are used at all.

Finally, we evaluated how the amount of available training data affects the performance of ROI prediction. This affects the prediction of all types of compound pairs, so we again report error rates. We partitioned the RepoRT training data into five uniform folds of 1–5, then trained model *i* using folds 1, …, *i* for *i* = 1, …, 4. For our model, we observe a massive performance improvement for larger training datasets, both for the regular dataset split (error rate 6.43% versus 4.89%) and the challenging dataset split (error rate 8.40% versus 5.65%) (Fig. 2d).

### Mapping retention order indices to retention times

Analyzing data from a small-molecule LC–MS/MS run often proceeds in phases: First, we annotate compounds using spectral libraries of authentic standards. Second, we annotate further compounds using in silico tools that search in molecular structure databases. Of note, we may assume that for any dataset, a subset of compounds has been annotated upfront with high confidence. Alternatively, a set of authentic standards may have been measured using the same chromatographic setup; notably, these compounds do not have to be present in the experimental sample. In the following, we will call these compounds anchors. Using spectral library search, some anchors may be incorrectly annotated, but restricting ourselves to high-confidence hits that agree with our biological intuition of the sample, we can ensure that the majority is correct.

The combination of ROI prediction and anchors allows us to finally tackle the problem of transferable retention time prediction: For a given molecular structure, we predict its ROI, then map the ROI to a retention time using a mapping function established on the known anchors (Fig. 1g). In the following, we will exclusively use the chromatographic condition-aware model for predicting ROI. We stress that for all results presented below, the target dataset was explicitly excluded from the training data of the ROI model.

**Fig. 3:**
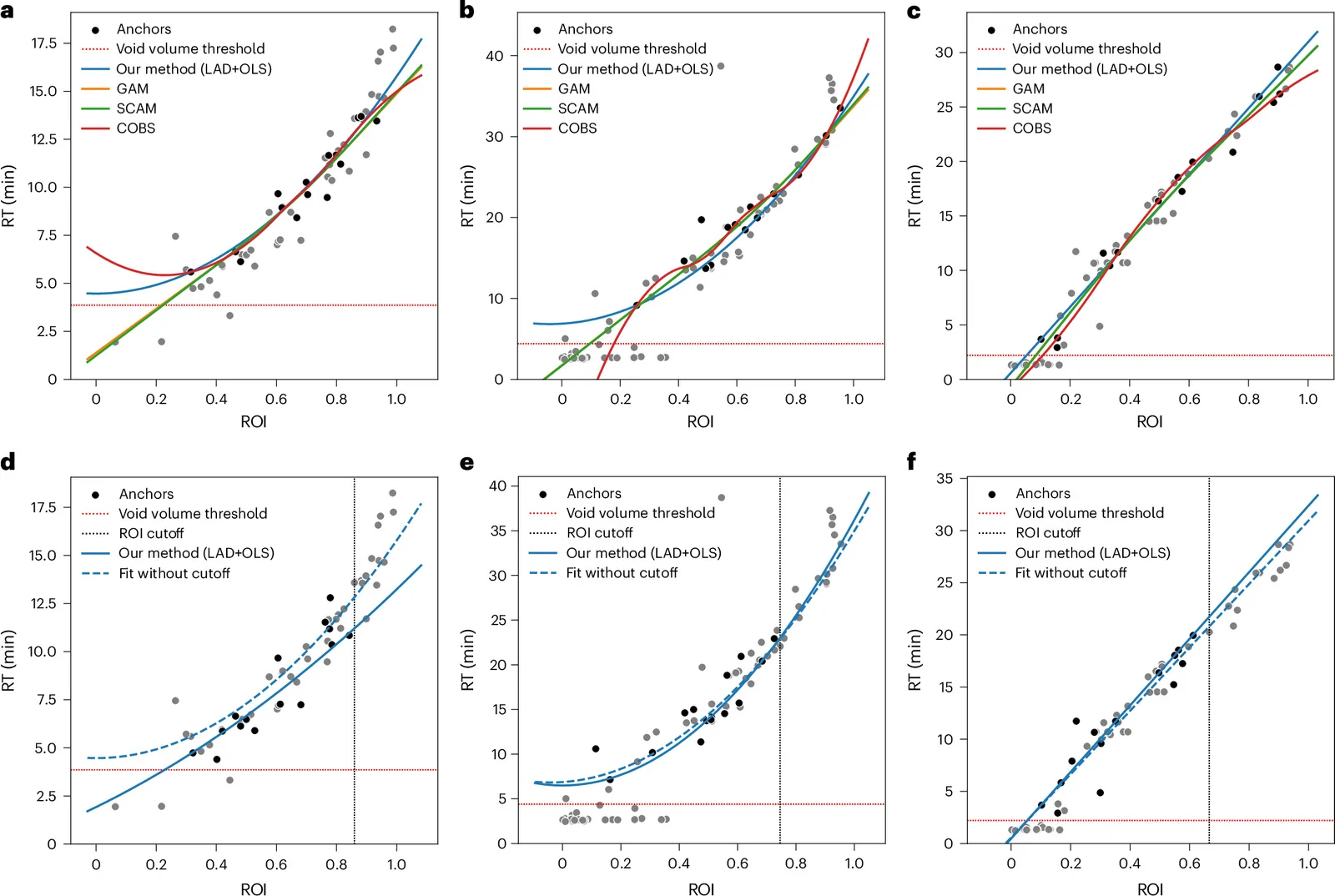
Retention order indices can be robustly mapped to retention time. A mapping is established using a polynomial of degree two, with coefficients estimated combining median regression (least absolute deviations; LADs) and ordinary least squares (OLS). **a**-**c**, For comparison, mappings using the robust regression methods GAM, shape constrained additive model (SCAM) and constrained B-Splines nonparametric regression quantiles (COBS) are shown. Only 15 randomly selected data points were used to establish the mapping, corresponding to an extremely low number of annotated anchor compounds. Compounds in the void volume were discarded. **d**–**f**, Excluding 25% of the compounds with the highest ROIs before randomly selecting 15 anchors. The small difference between mapping with and without exclusion indicates that the method can extrapolate. RepoRT datasets shown: 0270 (**a**,**d**), 0138 (**b**,**e**) and 0101 (**c**,**f**). Source data

**Extended Data Fig. 4:**
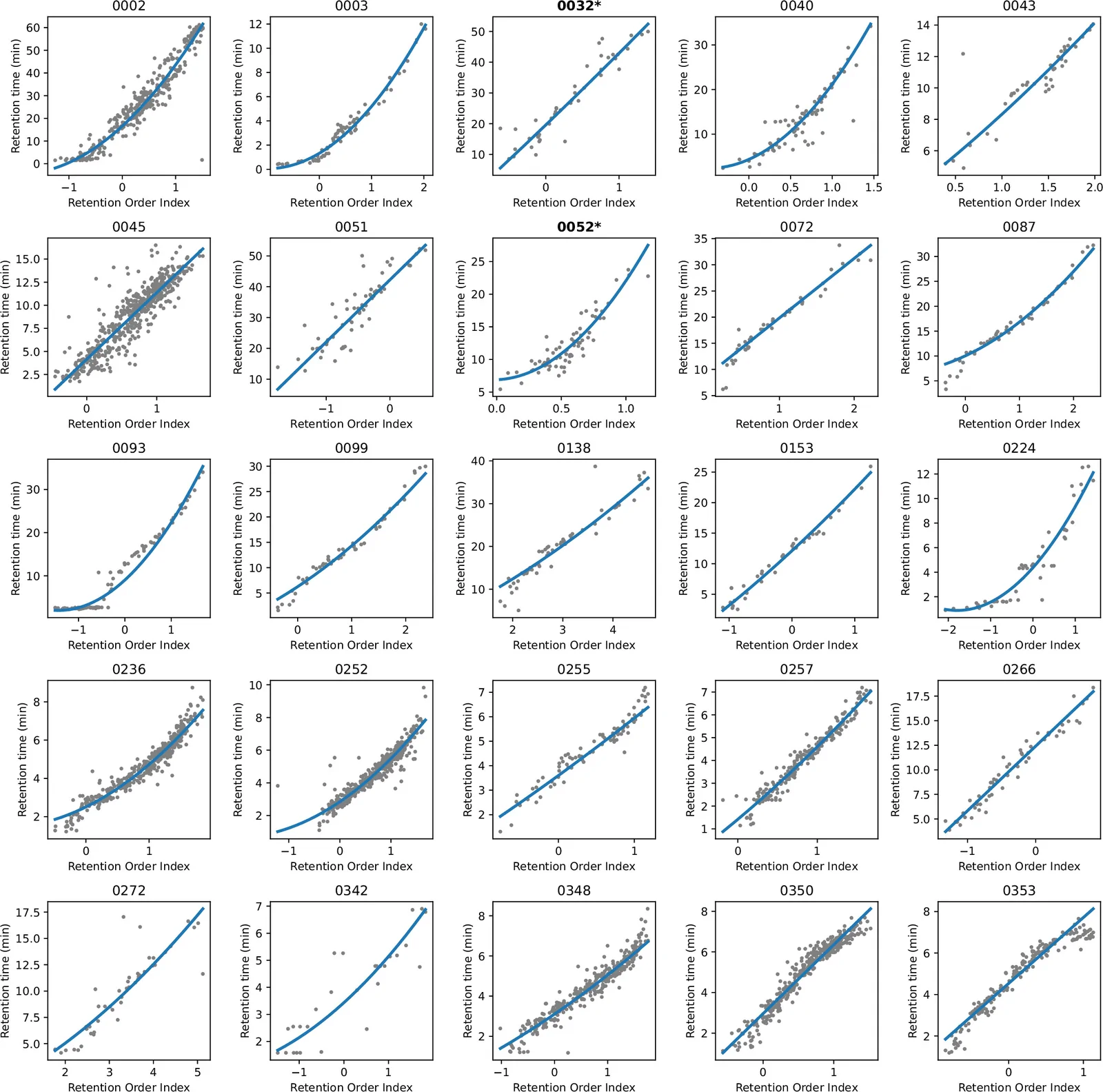
Mapping from Retention Order Indices to retention time for unknown chromatographic setups. For each fold of the regular chromatographic condition-disjoint cross-validation, five datasets were selected at random. Datasets with more than 50% of compounds eluting in the void volume were excluded beforehand. The mapping between ROI and RT was established using 80% randomly selected data points as anchors. Compounds in the void volume were excluded, for dataset 0051 the void volume threshold was chosen manually. Notably, for two datasets, the employed column was not part of the training data (marked with *), see Supplementary Fig. 3 for mappings of all datasets where this was the case. Source data

We found that we can robustly map ROIs to retention times using a polynomial of degree two (Fig. 3). To make our method robust against outliers such as falsely annotated anchors, we use median regression to exclude outliers, followed by a simple fit using ordinary least squares to establish the final mapping. We found that more intricate functions for establishing the mapping, such as generalized additive models (GAMs)^40^, performed worse (Fig. 3a–c). Notably, this highly restricted form of the mapping, with only three parameters, allows us to reliably map ROI to retention time even past the ‘latest’ anchor (Fig. 3d–f). Extended Data Fig. 4 shows 25 more datasets where the ROI model was trained setup-disjoint, and for two datasets even column-disjoint (Supplementary Fig. 3). Supplementary Fig. 4 shows datasets where mapping improves considerably when using the chromatographic condition-aware model.

In our final evaluation, we evaluate our 2-step method against best-in-class models for direct retention time prediction. At present, the best-performing methods for this task follow the pretrain/fine-tune paradigm^9–17^. Among the top-performing methods are DeepGCN-RT^15^ and RT-Transformer^16^ (refs. ^15,16^ provide a comparison against numerous other methods). DeepGCN-RT learns embeddings of molecules via a graph convolutional neural network with a high number of layers to predict retention time. RT-Transformer uses a similar architecture, but combines a graph attention network with a transformer. Both methods were pretrained on SMRT^41^, a large dataset of retention times (80,037 compounds), measured on one chromatographic setup. Both methods cannot carry out transferable retention time predictions; instead, models have to be fine-tuned (trained) on the actual target dataset. In evaluations, this is conducted using cross-validation. We evaluated 2-step against pretrain/fine-tune methods as 2-step (a method for transferable retention time prediction) has no directly comparable methods.

We concentrate on six datasets that have been repeatedly used for this type of evaluation. Reported errors were dominated by the gradient length of the respective target dataset, ranging from 6 min (dataset ‘LIFE old’) to 60 min (dataset ‘FEM long’). To make results comparable between datasets, we report errors for normalized retention time, with the gradient length being 100%. First, we retrained DeepGCN-RT and RT-Transformer using the SMRT dataset^41^; results were slightly worse than those reported elsewhere^15,16^. We attribute this to us removing duplicates (multiple identical entries) and not using void volume entries in the evaluation. Next, we used RepoRT to pretrain the both DeepGCN-RT and RT-Transformer with two strategies (single pretrain/fine-tune versus repeated pretrain/fine-tune). Of note, despite the extremely heterogeneous composition of RepoRT, this further improved results (Supplementary Table 2).

**Fig. 4:**
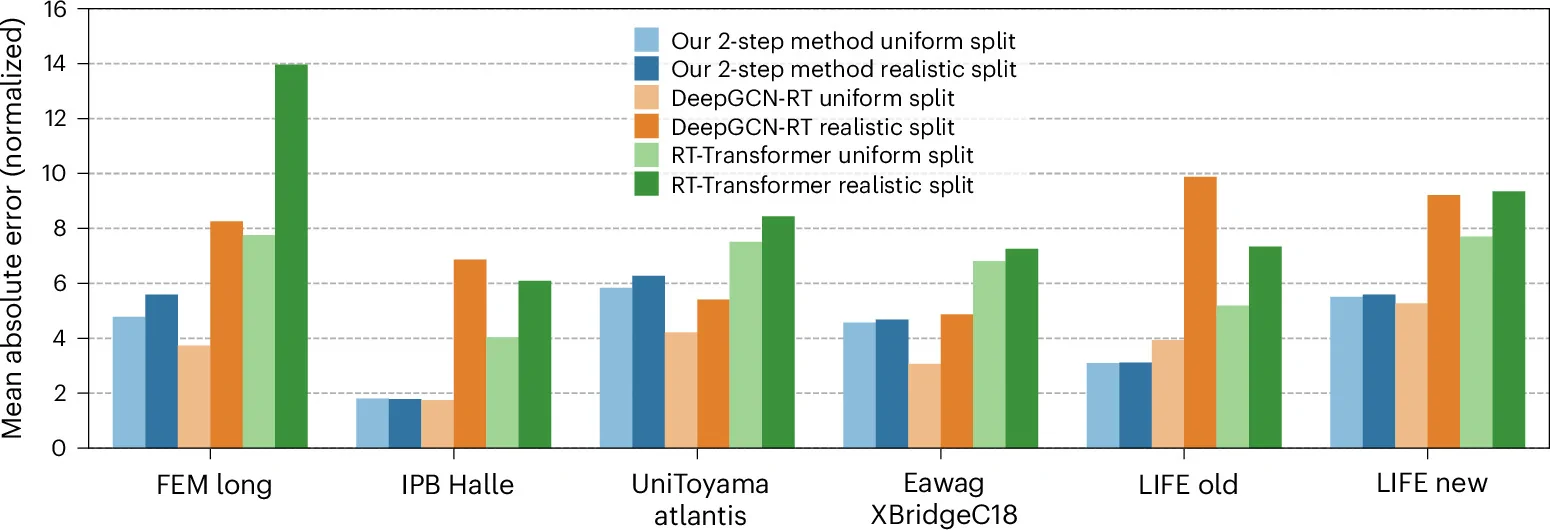
Comparison of retention time prediction performance for uniform versus realistic split. Mean absolute errors for the prediction of normalized RTs (in percentage points) are shown for the uniform and the realistic split of each target dataset. DeepGCN-RT and RT-Transformer were pretrained on RepoRT (repeated mode), as this resulted in overall best performance (Supplementary Table 3); models were fine-tuned and evaluated using cross-validation on the target dataset. Training of the ROI prediction model is performed without the target datasets, and anchors are established by cross-validation on the uniform or realistic split (‘2-step’).

For our 2-step method, we trained the ROI model excluding all target datasets from the training data, and evaluated the mapping from ROI to retention time using the above cross-validation split for selecting anchors. Performance of the 2-step method is basically on par with DeepGCN-RT and RT-Transformer for the uniform split (Fig. 4 and Supplementary Table 2). Recall that our machine-learning model has not seen the target dataset, and that anchors can be established using high-confidence spectral library hits.

**Extended Data Fig. 5:**
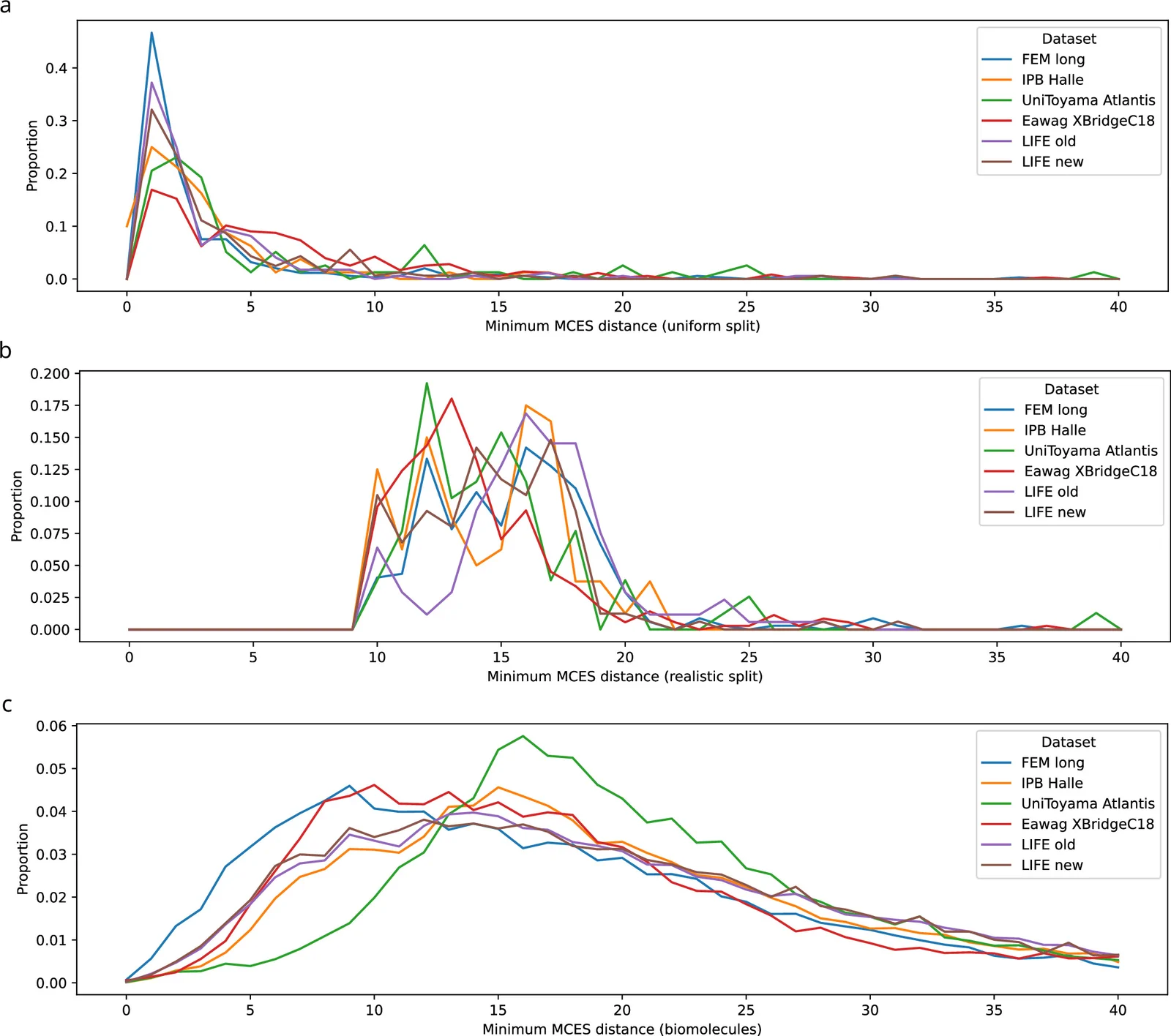
Maximum Common Edge Subgraph (MCES) distances for different splits. We consider fine-tune datasets FEM long, IPB Halle, UniToyama Atlantis, Eawag XBridgeC18, LIFE old, and LIFE new. We report the distribution of minimum MCES distances for any molecular structure against the training data. Assume molecular structure *x* is part of fold *i* of the split. In cross-validation, the performance on *x* is evaluated using a model trained on all folds except *i*. We find the smallest distance between *x* and all molecular structures *y* from all folds except fold *i*. The well-founded assumption is that a highly similar molecular structure in the training data, will make it easier for the model to do a prediction for molecular structure *x*. We compute myopic MCES distances^45^ with distance threshold *T* = 50, meaning that all distances reported here are exact. MCES values are rounded down to integer values. (**a**) Minimum MCES distances for a uniform (random) split. For the vast majority of molecular structures, the training data contains a highly similar molecular structure with distance well below 10. (**b**) Minimum MCES distances for a realistic split from^44^ with distance parameter 10. As intended, this split pushes minimum distances to larger values. (**c**) Minimum MCES distances of biomolecular structures against all of the respective fine-tune datasets. We use a uniform subsample of 20k molecular structures from a collection of biomolecular structure databases, see^45^ for details. This collection of databases is a realistic set of structures for which we would want to apply retention time prediction; for example, this set is used when searching with CSI:FingerID^68^. Whereas there are few compounds with small minimum distances (MCES up to 10 for 6.7% to 29.1% of a dataset), we observe far more compounds with large distances (MCES 20 and above for 38.6% to 52.4% of a dataset), substantially exceeding what we have observed for the realistic split. Source data

It is well known that a uniform splitting of small molecules, as performed in previous evaluations^9–16^, will result in a substantial overestimation of the power of a machine-learning model^42^. Notably, even with scaffold splits we tend to overestimate model performance^43^. A model will perform well if there is a sample in the training data highly similar to the sample for which we want to make a prediction. We repeated the above evaluation using a more realistic cross-validation split introduced elsewhere^44^, which explicitly excludes such cases. This realistic split is based on myopic maximum common edge subgraph distances, which basically count the number of edit operations (reactions) needed to transform one molecular structure into another^45^, and agrees well with chemical intuition. The cross-validation split ensures that structures in different folds are never structurally highly similar (Extended Data Fig. 5ab). We refer to this split as ‘realistic’ as it better captures the domain of compounds for which we do predictions^45^. In metabolomics applications (Supplementary Note 1), methods must do predictions for all biomolecules of interest. Of note, even this realistic split is too optimistic. A large fraction of biomolecules displays a much higher distance to the training data than what we observe in the realistic split (Extended Data Fig. 5bc).

For the realistic split, we observed a large drop in performance of DeepGCN-RT and RT-Transformer for most target datasets (Fig. 4). In comparison, our 2-step method exhibits no, or a negligible, drop in performance. The 2-step method outperformed the other methods for five of six datasets, and performed roughly on par for the last one (Table 1). Notably, the 2-step method outperformed the best available pretrain/fine-tune models without having seen the target datasets. For other statistical measures, we observe basically identical trends (Supplementary Table 3). Different from our ROI model, pretrain/fine-tune methods do not improve in performance when more data are available for pretraining (Supplementary Fig. 5).

**Table 1:** Comparison of retention time prediction performance for the realistic split

Method	Pretrain	FEM long	IPB Halle	UniToyama Atlantis	Eawag XBridgeC18	LIFE old	LIFE new
DeepGCN-RT		10.51 ± 2.81	11.37 ± 5.48	6.46 ± 2.66	5.15 ± 0.74	38.54 ± 16.56	29.75 ± 11.70
RT-Transformer	SMRT	15.17 ± 4.80	6.19 ± 4.43	7.54 ± 2.80	4.82 ± 0.98	6.88 ± 1.36	9.93 ± 2.57
DeepGCN-RT	single	11.49 ± 3.67	4.87 ± 2.78	10.38 ± 4.74	5.35 ± 0.95	6.37 ± 2.05	7.92 ± 5.43
RT-Transformer	RepoRT	12.78 ± 1.66	6.12 ± 1.75	6.76 ± 3.07	6.52 ± 0.82	6.64 ± 3.47	11.12 ± 8.16
DeepGCN-RT	repeated	8.26 ± 2.57	6.87 ± 2.81	***5.41*** ± 1.99	4.87 ± 1.18	9.87 ± 3.37	9.21 ± 2.34
RT-Transformer	RepoRT	13.96 ± 4.62	6.09 ± 3.77	8.43 ± 3.63	7.26 ± 1.42	7.34 ± 2.31	9.34 ± 2.41
Our 2-step method		***5.59*** ± 1.03	***1.78*** ± 0.45	6.28 ± 2.73	***4.68*** ± 4.79	***3.12*** ± 0.77	***5.59*** ± 1.36

Mean absolute errors and s.d. (over cross-validation folds) for the prediction of normalized retention times (in percentage points) are shown. Pretrain/fine-tune methods were trained and evaluated by cross-validation, using the realistic split of the target dataset. Either SMRT or RepoRT were used for pretraining, the latter either using single pretrain or repeated pretrain. In contrast, training of the ROI prediction model is performed without the target datasets (‘our 2-step method’). The lowest errors per dataset are highlighted in bold.

Finally, we performed ablation studies, removing training data of the ROI model. As above, the target dataset was never part of the training data. First, we removed all datasets that have a similar chromatographic setup (same column and similar pH) as the target dataset. Second, we removed all compounds that are present in the target dataset, including stereoisomers. Third, we performed a ‘maximum challenge’ evaluation, removing both compounds and similar datasets. This variant has neither seen the chromatographic setup nor the compounds it is evaluated on. In all three cases, we observe only a slight drop in performance. Notably, even with the ‘maximum challenge’ evaluation the 2-step method outperformed the pretrain/fine-tune methods for the realistic split. Fourth, we reduced the number of anchors for mapping ROI to retention time; here, we use the original model for predicting ROI. Notably, as few as 15 anchors are sufficient to establish the mapping (Supplementary Tables 3 and 4). For completeness, non-normalized errors for all ablation studies are shown in Supplementary Table 5.

**Extended Data Fig. 6:**
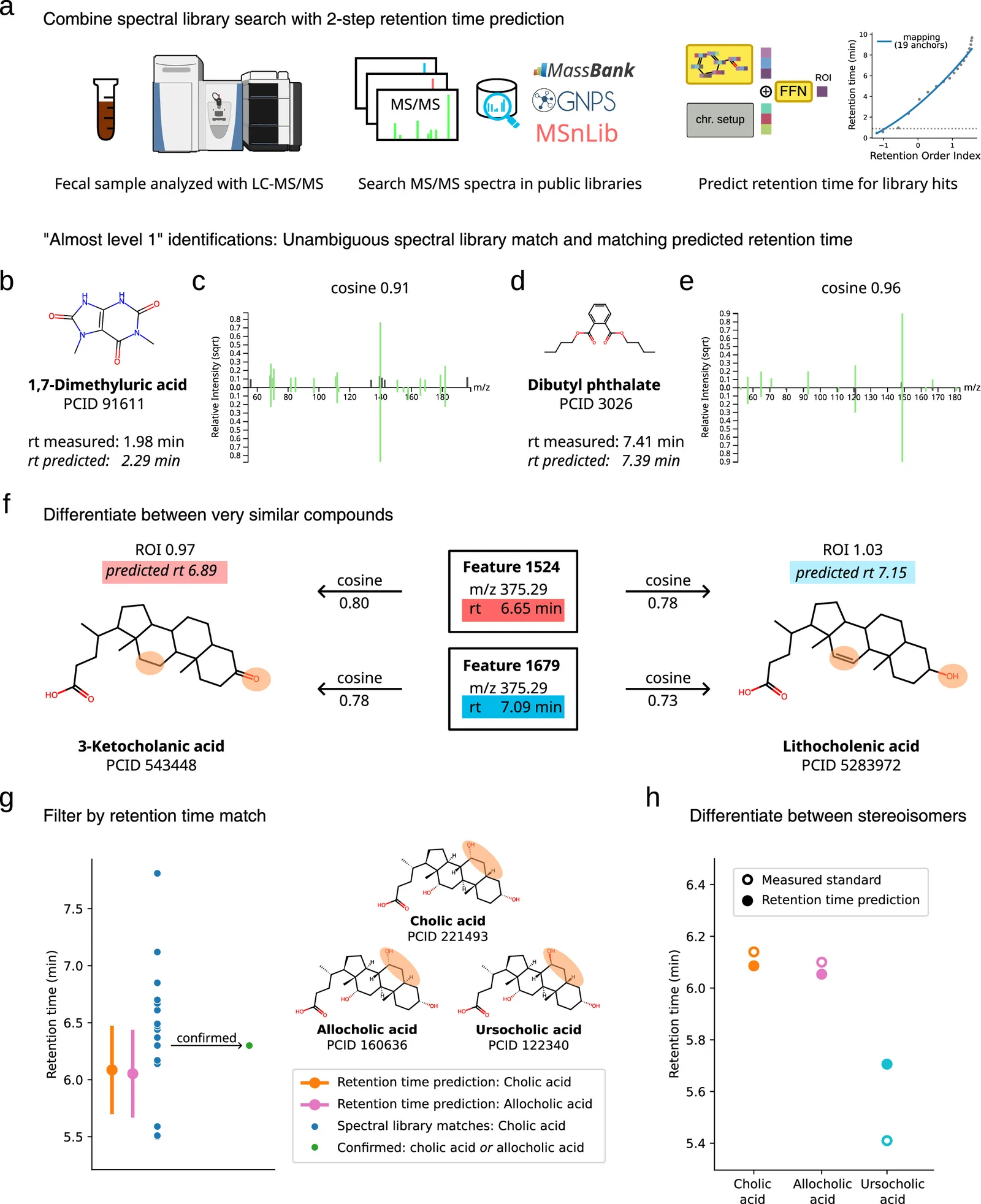
Applications for 2-step in an analyzed fecal sample. Compare to Supplementary Note 1. (**a**–**g**) A LC–MS/MS run from a fecal sample was analyzed combining spectral library search and 2-step retention time prediction. Public spectral libraries MassBank, MassBank of North America (MoNA), GNPS, and MSnLib were searched. Mapping ROI to retention time using 19 N-Alkylpyridinium 3-sulfonate standards. Retention time was predicted for all spectral library compounds. (**b**–**e**) Two query spectra with excellent spectral matches and matching retention times, resulting in “almost level 1” identifications, see Supplementary Table 6 for additional hits. (**f**) Predicted retention times allow us to differentiate between highly similar compounds with ambiguous spectral library matches. Two query spectra resulted in good spectral library matches (cosine above 0.73) to two different compounds (3-ketocholanic acid and lithocholenic acid). Notably, differentiating between those compounds is possible using (predicted) retention times. (**g**) For cholic acid, retention time prediction helped filtering features with spectral library hits. One feature in the predicted retention time range was confirmed with an internal standard as either cholic acid or allocholic acid; these diastereomers can not be differentiated with the used experimental setup. Bars indicate the applied retention time threshold of 0.40 min. (**h**) Predicted retention times for diastereomers of cholic acid can differ substantially, and match excellently with measured retention times. The same experimental setup as in (**a**–**g**) was used. Note the varying y axis.

To showcase practical applications of the 2-step method, we analyzed a LC–MS/MS dataset of human fecal samples. Of note, retention times of only 19 authentic standards (*N*-alkylpyridinium 3-sulfonate) were known for this dataset. Consequently, both the very small number of measurements that could be used as training data, as well as the high structural similarity of the authentic standards make it impossible to use any pretrain/fine-tune methods (including DeepGCN-RT and RT-Transformer) or direct prediction methods for this dataset. Notably, this situation is the rule and not the exception for metabolomics research (Supplementary Note 2). Using transferable retention time prediction we identified several compounds at ‘almost level 1’, resolved ambiguities of spectral library search for bile acids, showcase how to resolve stereoisomer annotations (Extended Data Fig. 6), and also demonstrate how in silico annotations can be improved (Supplementary Fig. 6).

### Influence of chromatographic conditions on retention order

We now analyze what metadata about chromatographic conditions are required for the analysis of small-molecule retention in a dataset. Given that retention times vary even for nominally identical conditions (Extended Data Fig. 1), we concentrate on the retention order using reversed-phase chromatography. Naturally, we are interested in a minimum set of parameters. The fewer the fields to fill in, the more likely it is that scientists will include these metadata when uploading a dataset to a repository.

We observed above that the minimum information unit to describe a change in retention order is a conflicting pair. These are two compounds A and B that elute in a different order in two different datasets (A before B in dataset 1 versus B before A in dataset 2). Conflicting pairs are not restricted to pairs of compounds with highly similar retention time. In fact, if the number of conflicting pairs between two datasets is large, this implies that there must exist large global differences in retention order between the two datasets (Extended Data Fig. 2). For ‘local changes’ or if only a few compounds are ‘causing conflicts’ (Extended Data Fig. 3), this number is small.

First, we analyzed the fraction of conflicting pairs in RepoRT (Supplementary Table 7). We find that 2.0% of compound pairs are in conflict for 10 s retention time threshold, 0.7% for 30 s and 0.3% for 60 s retention time difference. In fact, the situation is more severe than what these numbers indicate: We may observe zero conflicting pairs between two datasets simply because the compound pairs shared between them will never change order, see the discussion on monotonous pairs versus informatory pairs. Restricting our analysis to informatory pairs, 23.8% are in conflict for 10 s, 20.3% for 30 s and 18.0% for 60 s retention time difference. We conclude that changes in retention order are a common phenomenon for small-molecule reversed-phase chromatography.

**Extended Data Fig. 7:**
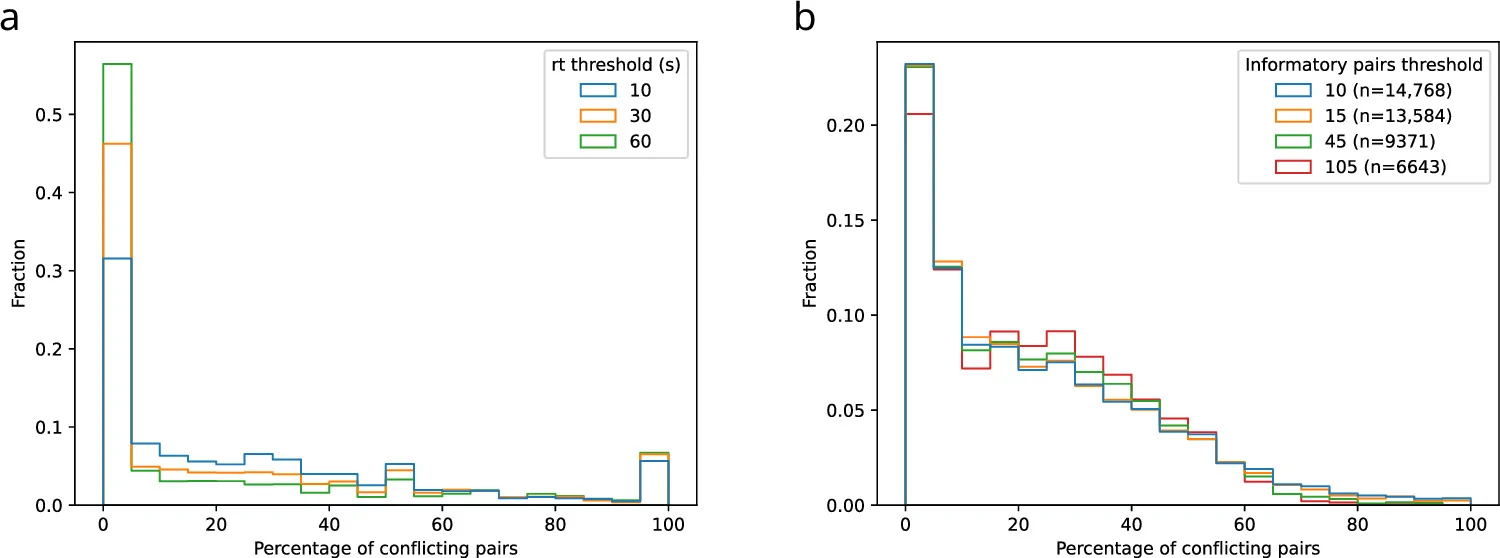
Conflicting pairs as a measure of retention order change. (**a,b**) Empirical distributions of dataset pairs when varying the fraction of conflicting pairs. Despite the unavoidable differences, we basically observe identical trends. (**a**) Three thresholds for retention time difference of a *compound pair* to be considered informatory, not using a threshold on the number of informatory pairs. (**b**) With the retention time threshold kept at 10 s, the threshold for the number of informatory compound pairs per *dataset pair* is varied. Number *n* of remaining dataset pairs when varying the threshold. Note the varying y axis. Source data

The above statistics average over all datasets in RepoRT, but hide the extent to which any two datasets can be in conflict. Consequently, for any two datasets, we now consider the fraction of informatory compound pairs in conflict; we refer to this number as the fraction of conflicting pairs. We study the empirical distribution of dataset pairs with a particular fraction of conflicting pairs. Clearly, this fraction depends on the chosen retention time threshold, but trends are similar for different thresholds (Extended Data Fig. 7a). Unfortunately, two artifacts dominate the shown empirical distributions. If two datasets share only a small number of compounds that can be in conflict, then we often observe that 0% or 100% of those compounds are in conflict. We found that 37.4% of dataset pairs do not share a single informatory compound pair, and 59.3% have fewer than $$15=\left(\begin{array}{c}6\\ 2\end{array}\right)$$ informatory pairs, roughly corresponding to six shared compounds. Hence, we introduce a threshold on the number of informatory pairs for a dataset pair to be included in our statistics. The variation in the fraction of conflicting pairs for different threshold values is shown in Extended Data Fig. 7b. We observe similar trends for different thresholds, indicating that a large fraction of those compounds that can vary, do vary between datasets and, hence, chromatographic conditions. In the following, we only consider the 13,584 dataset pairs with at least 15 informatory pairs, unless stated otherwise. For nominally identical chromatographic conditions, retention order is indeed conserved, with an average of 4.6% conflicting pairs as opposed to 22.7% when comparing all dataset pairs with shared and different chromatographic setups (Supplementary Fig. 7). We now study chromatography conditions that influence retention and selectivity in LC, including stationary phase, mobile phase composition, temperature and flow rate.

**Fig. 5:**
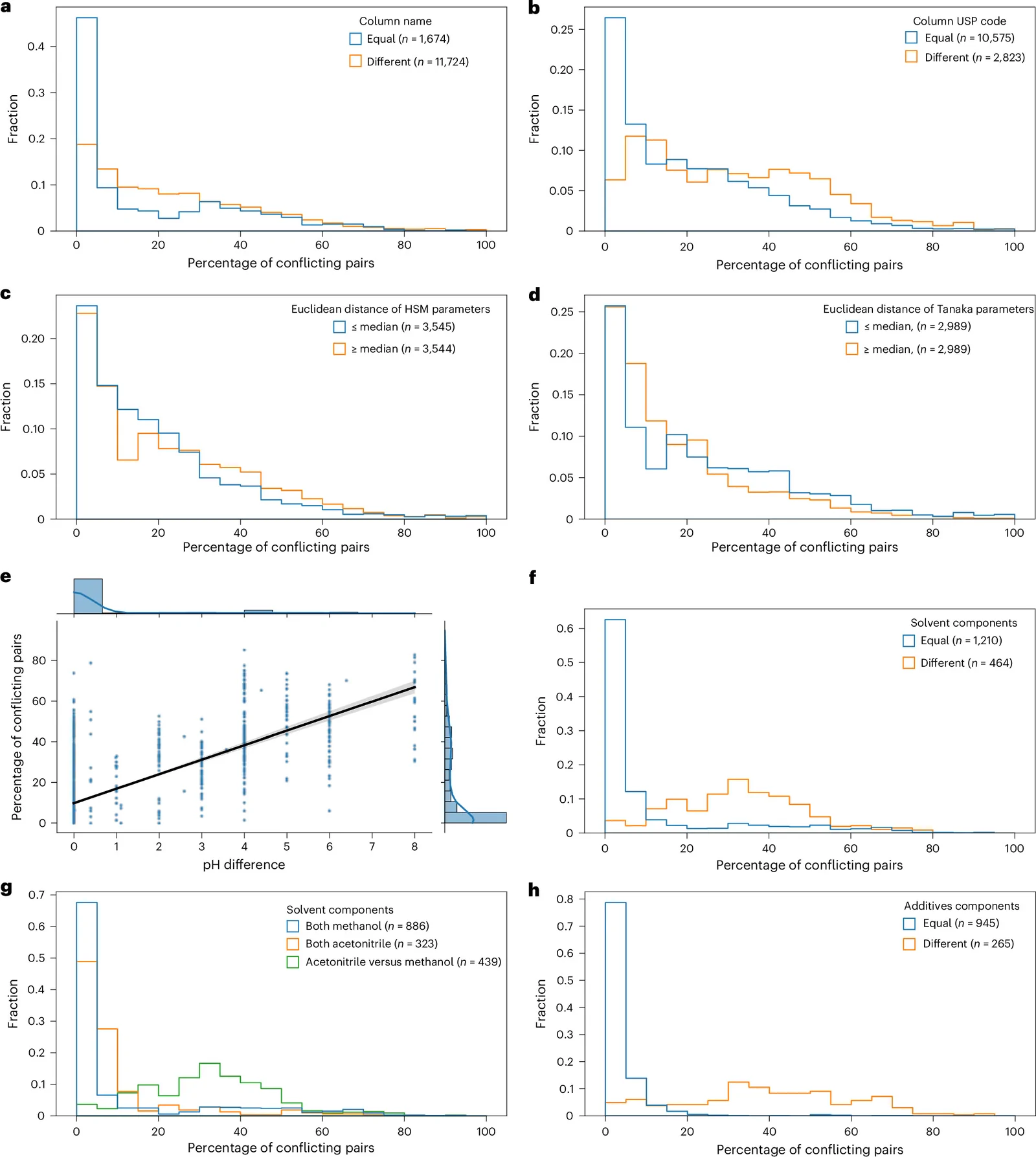
Influence of stationary and mobile phase on retention order. **a**–**d**, Influence of stationary phase: Histogram plots of conflicting pairs percentages for pairs of datasets, comparing distributions for equal versus unequal column name (**a**) and USP code (**b**), similar versus dissimilar HSM (**c**) and Tanaka parameters (**d**). Dataset pairs with nominally identical setup excluded (**a**,**b**). Datasets pairs with the same column are excluded (**c**,**d**). Similarity measured by Euclidean distances, and split into two sub-distributions using the median distances. **e**–**h**, Influence of mobile phase. Only dataset pairs with the same column are considered, whereas dataset pairs with nominally identical setup are excluded. Influence of pH value difference with regression line and marginal distributions (**e**). The translucent band around the regression line represents the confidence interval of 95%. Estimated pH values and differences are mainly integers. Histogram plots of conflicting pairs percentages for pairs of datasets, comparing solvent components (**f**,**g**) and additive components (**h**). Only dataset pairs with equal solvent components were considered (**h**). Number *n* of dataset pairs for each distribution. Note the varying *y* axis. Source data

Arguably, the largest effect on retention is due to the employed chromatographic column (stationary phase). Indeed, datasets with different columns have a substantially larger fraction of conflicting pairs than those with identical columns (Fig. 5a). Similarly, columns with different United States Pharmacopeia (USP) codes have a higher fraction of conflicting pairs than those with identical USP code (Fig. 5b). As one may expect, differences are less pronounced than for column names. To measure differences between columns, we use the Euclidean distance of the columns’ HSM and Tanaka parameters, as two parameter sets describing column chemistry. We find that both for HSM and Tanaka parameters, column pairs with small distance show a lower fraction of conflicting pairs than those with large distances (Fig. 5c,d). The effect is more pronounced for HSM parameters, but substantially smaller than when directly comparing column names. Notably, there exist several dataset clusters in RepoRT where a fixed set of small molecules is measured on the same column, varying other individual chromatographic parameters.

**Extended Data Fig. 8:**
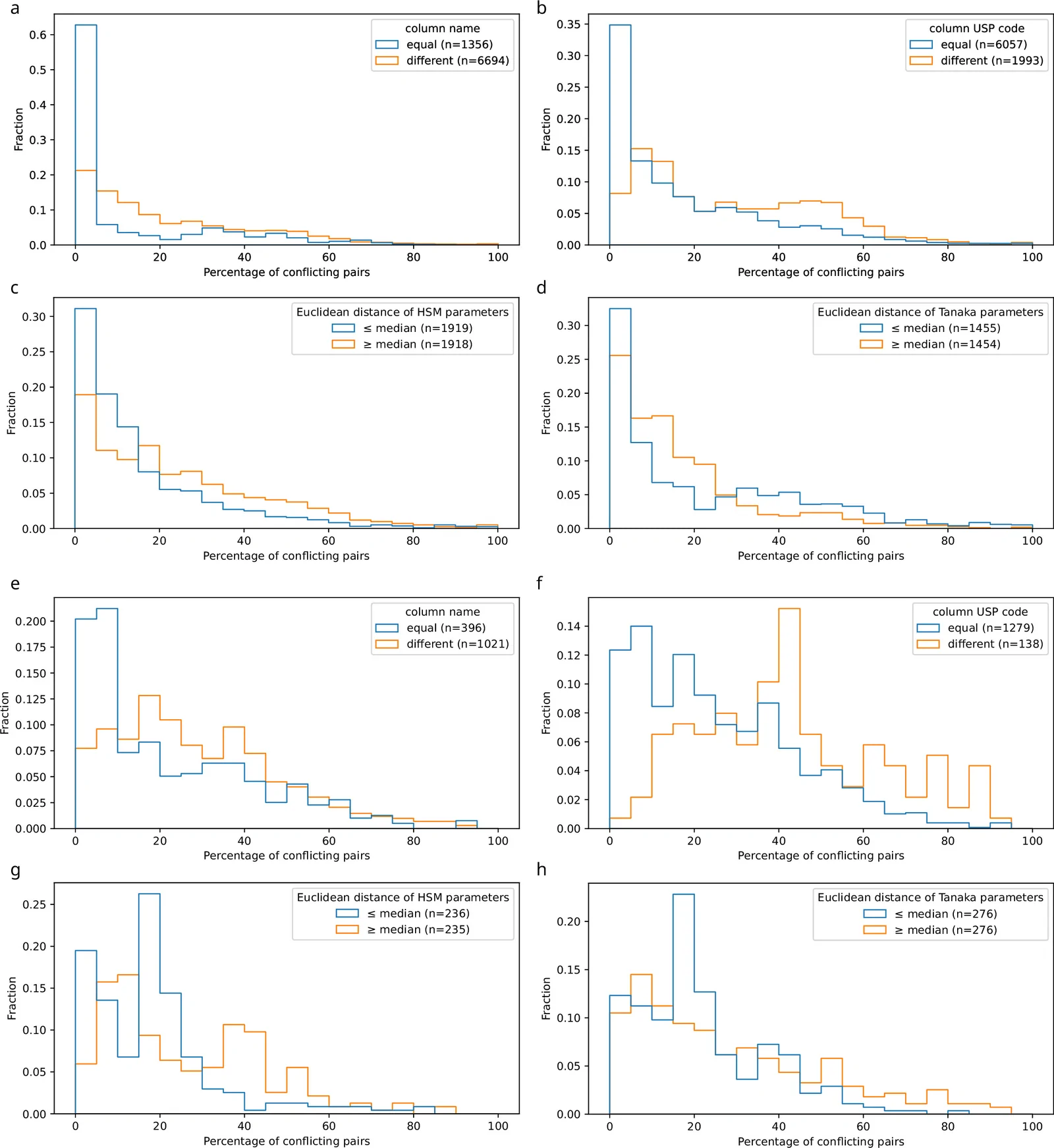
Influence of column choice on retention order for dataset clusters. Analogous to Fig. 5**a**–**d**, histogram plots of conflicting pairs percentages for pairs of datasets are shown, comparing distributions for equal versus unequal column name (**a**,**e**) and USP code (**b**,**f**), similar versus dissimilar HSM (**c**,**g**) and Tanaka parameters (**d**,**h**). Here, the two largest dataset clusters of RepoRT are examined separately: Subfigures a–d show conflicting pairs percentages of only the 143 datasets in these clusters. These datasets are excluded for subfigures **e**–**h**, leaving 92 datasets. Datasets pairs with the same column are excluded in **c**,**d** and **g**,**h**, respectively. The two largest clusters are datasets measured exclusively for RepoRT34, where column, eluents and temperature were systematically varied, and datasets of ^69^, where varying combinations of column, pH, injection volumes, eluents, and additives were compared. Number *n* of dataset pairs for each distribution. Note the varying y axis. Source data

As shown in Extended Data Fig. 8, the trends observed for the whole of RepoRT are also present when examining these dataset clusters separately or excluding them from statistics.

After the chromatographic column, we studied in a second step the influence of eluent composition (mobile phase) (Fig. 5e–h). For datasets with the same column, a clear relationship between eluent pH and conflicting pairs is visible. Unsurprisingly, dataset pairs sharing the same solvent components have a higher degree of retention order agreement. Focusing only on datasets whose solvents are based on either methanol or acetonitrile, again, dataset pairs sharing the same solvent exhibit higher conservation, while the conservation seems to be stronger for methanol-based solvents. Finally, differences are also clearly visible when comparing different additive compositions.

**Extended Data Fig. 9:**
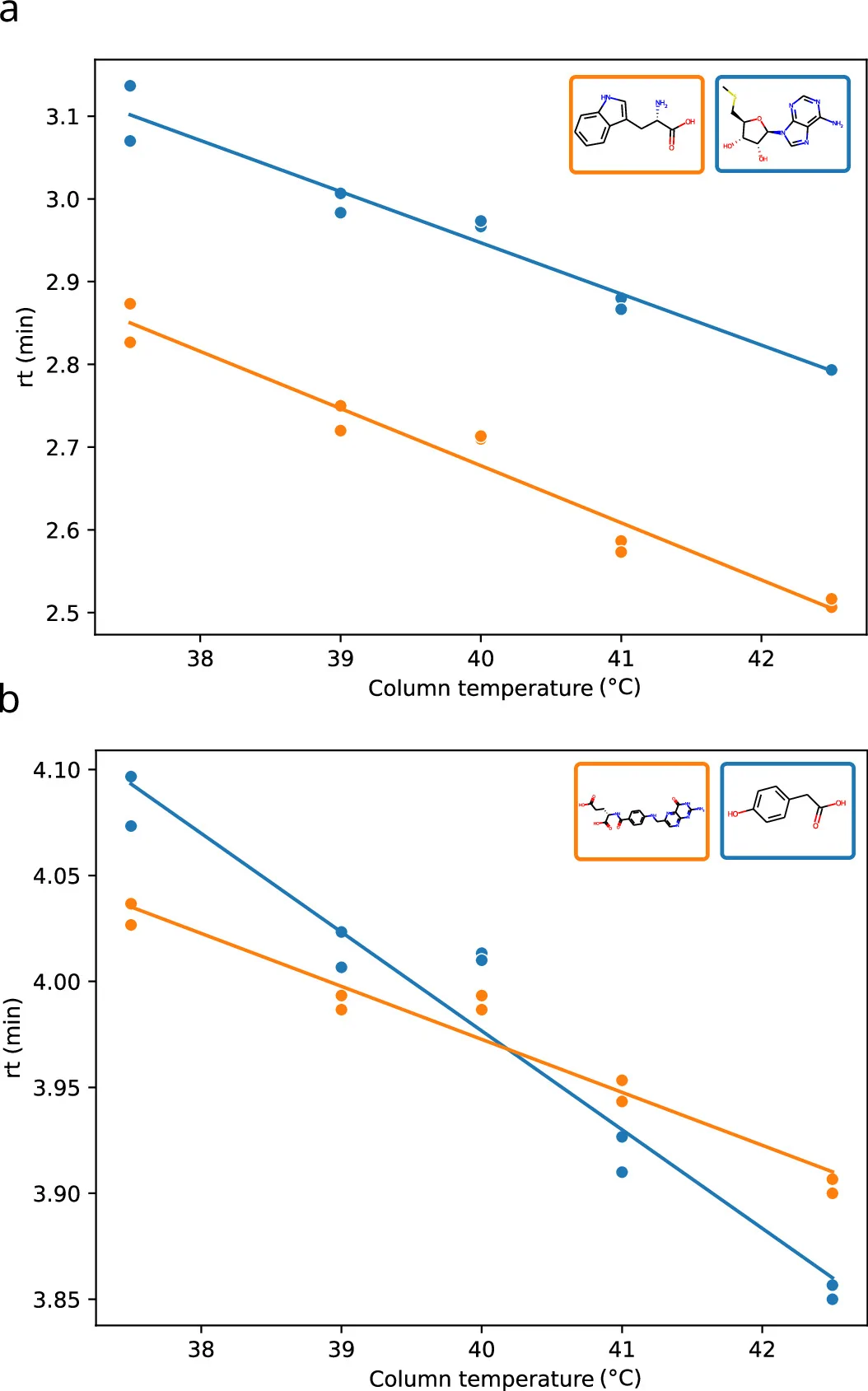
Influence of temperature on retention is compound-dependent. The variation of the column temperature does not influence the retention behavior of all compounds equally. With all other chromatographic settings kept constant, the retention order of two compounds over a series of temperature variations can stay the same (**a**) or flip (**b**). In these examples, the values of two datasets per temperature setting are shown. The displayed compounds are (**a**) L-tryptophan (orange) and methylthioadenosine (blue), (**b**) folate (orange) and p- hydroxyphenylacetate (blue); both examples are from RepoRT datasets 0187,0188,0197-0204. Source data

Third, we studied the influence of temperature on retention order. Only some compounds show temperature-induced flips in retention order (Extended Data Fig. 9). We investigated ten clusters of datasets where only the column temperature was varied. In all but one cluster, dataset pairs with a higher difference in column temperature exhibit a higher number of conflicting pairs (Supplementary Fig. 8); however, temperature-induced flips can only be observed for very low thresholds of retention time difference. Fourth, we found that changes in flow rate did not result in substantial retention order variations (Supplementary Fig. 9).

## Discussion

Retention order is well preserved for nominally similar conditions, whereas retention times may vary substantially. Consequently, the task of directly predicting retention times is somewhat ill-posed. Here, we suggest to instead, concentrate on the prediction of retention order indices. Doing so ‘frees’ the machine-learning models from predicting the nonlinear, yet comparatively ‘uninteresting’ warping of the retention time domain.

The combination of ROI prediction and retention time mapping via our 2-step approach, finally enables transferable prediction of retention times. The mapping can be achieved using low-order polynomials and using few anchors, making the method highly robust. In particular, ROI and retention time prediction is possible even for novel columns absent from the training data. We demonstrated that including chromatographic parameters considerably improved the model’s accuracy. Prediction accuracy is better than state-of-the-art pretrain/fine-tune methods, but does not require measuring references on the target system (Supplementary Note 2). The integration of 2-step with methods for spectral library search, in silico methods and methods for de novo structure generation constitutes relevant and promising future work.

For the benefit of the community, we ask that reference LC–MS datasets are publicly provided, including the abovementioned metadata on chromatographic conditions^46^. Models may use chromatographic features beyond column chemistry and pH when more training data become available. Transferable prediction requires HSM or Tanaka parameters of the column; we ask column vendors to make these parameters available. In our experiments, regression beyond polynomials did not improve predictions results, but this clearly depends on the target datasets. Better ROI prediction accuracy will enable retention time prediction even for exceptional chromatographic gradients using more intricate methods for mapping ROI to retention time (Fig. 3). Consequently, we conjecture that huge improvements in transferable retention time prediction will be possible in the near future. HILIC separation mechanisms are even more diverse than for reversed-phase, and no generally agreed parameters exist to describe column selectivity, making transferable retention time prediction for HILIC the next big challenge.

We found that certain chromatographic parameters influence retention order: Namely, column, mobile phase, gradient, temperature, and flow rate. In the future, these parameters should be recorded when datasets are stored in repositories, to allow reuse of retention times in such datasets. Notably, our findings suggest that recording these parameters may be sufficient; no further parameters are required as minimum information about an LC experiment. Retention times can be recovered from ROIs using our 2-step approach. Clearly, we cannot rule out that other chromatographic parameters are important for certain experimental settings (Supplementary Fig. 7); yet, given the number of analyzed datasets, we argue that the influence should usually be small.

## Methods

Throughout this paper, we say that a model for retention time is transferable if it allows us to predict retention times for a chromatographic setting that was not part of the training data.

To estimate the number of publications that deal with LC for analyzing metabolites and other small molecules, we used (‘small molecules’ OR ‘metabolite’ OR ‘metabolites’) ‘liquid chromatography’ as a query for Google scholar. On 20 Apr 2026 this resulted in 2.36 million hits. To estimate the number of publications that deal with retention time prediction of metabolites and other small molecules, we used (‘retention time prediction’ OR ‘predict retention times’ OR ‘predict retention time’) (‘small molecules’ OR ‘metabolite’ OR ‘metabolites’) as a query for Google scholar. On 20 Apr 2026 this resulted in 2,430 hits. Similarly, searching Google scholar for ‘quantitative structure-retention relationship’ resulted in 1,960 hits. It is understood that these numbers are only proxies of the actual number of studies.

### Curation of training and evaluation data

Training and evaluation of our methods are based on 297 reversed-phase datasets from RepoRT^34^, v.94f43c1b from August 2024. As suggested elsewhere^34^, we excluded the SMRT dataset^41^ from our analysis, as it differs substantially from all other datasets and tends to dominate statistics due to its size. For brevity, we refer to the remaining 297 reversed-phase datasets as the RepoRT repository.

For training and evaluating retention order prediction models, we discarded 15 datasets (0022, 0046, 0205, 0207, 0214, 0215, 0265, 0325, 0341 and 0357–0362) for which no more than 20 compounds were measured. We manually discarded 23 datasets for which retention time data was only recorded for a small section of the gradient (0006, 0008, 0023, 0059, 0123, 0128, 0130–0133, 0136, 0137, 0139, 0143, 0145, 0148, 0149, 0151, 0152 and 0154–0157). We manually discarded two datasets (0056 and 0057) that were measured under nominally identical setup, but showed a suspiciously high number of conflicting pairs (Supplementary Fig. 7). One dataset (0024) uses a step-wise gradient, unlike the ramp gradient of all other datasets. We manually discarded this dataset. Notably, its retention order is substantially different from all other datasets, with 94.2% conflicting pairs on average.

HSM parameters^37^ and Tanaka parameters^38^ have to be determined experimentally, using specific authentic standards and analytical conditions to characterize the properties of the column. Unfortunately, these experiments are somewhat challenging to perform, as the required conditions are not compatible with LC–MS setups used in metabolomics. Consequently, these parameters are not available for all columns. For the 256 remaining datasets, either pH, HSM or Tanaka parameters were missing for 85 datasets. We excluded these datasets. The remaining 171 datasets are referred to as the RepoRT training data.

The void volume is the volume of the column and the capillaries in the chromatographic system. Not retained compounds will elute at the leading tail of the injection plug and pass through at the dead time. This time can be estimated if the void volume and flow rate are known. As the actual void volume of the datasets is not known, we used twice the estimated value provided in RepoRT as the void volume threshold and calculated the dead time accordingly. We find that 22.1% of entries elute within the void volume of their respective dataset. Recall that compounds pairs where only one compound is eluting in the void volume, are used for training and evaluation of the ROI models. Annotations for void volume are missing for 37 datasets. For these datasets, we assume a dead time of 0, corresponding to no compounds eluting in the void volume. We added information to RepoRT on what datasets should not be used to train and evaluate models (column ‘model.flags’).

To evaluate prediction of retention times, we resorted to six datasets frequently used in the field for this purpose, all of which are contained in RepoRT: FEM long (RepoRT dataset 0002) with 399 compounds and gradient length 60 min^47^, IPB Halle (0003) with 80 compounds and gradient length 20 min^48^, UniToyama Atlantis (0018) with 78 compounds and gradient length 40 min^49^, Eawag XBridgeC18 (0019) with 364 compounds and gradient length 30 min^50,51^, LIFE old (0054) with 172 compounds and gradient length 6 min^52^, and LIFE new (0055) with 162 compounds and gradient length 7 min^52^. Notably, we used the curated versions of these datasets as contained in RepoRT, with standardized SMILES. The unprocessed datasets contain duplicate entries (identical retention of a compound reported more than once) and doublets (multiple retention times for the same compound). For duplicate entries, only a single copy was kept; doublets were removed completely for evaluation, following^34^. No gradient length was recorded for UniToyama Atlantis; we estimated the gradient length to be 40 min, based on the available retention time data. Compounds eluting in the void volume were not considered for evaluation as well, again using twice the estimated value provided in RepoRT as threshold.

For the evaluation of retention time prediction, we removed all target datasets from the training data, training one ROI prediction model on all remaining RepoRT datasets. For the evaluation ‘without similar datasets’, we excluded the test datasets, as well as all datasets with the same column and similar mobile phase pH value (difference less than 1, see above) from the training data of the ROI model. For FEM long, IPB Halle and LIFE new, 19 similar datasets were excluded. For LIFE old, 39 similar datasets were excluded. For UniToyama Atlantis, and Eawag XBridgeC18 there are no such datasets; therefore, only the test datasets were excluded. We perform this evaluation for each dataset individually, meaning we train one ROI prediction model per dataset, removing similar datasets only for the current target dataset. For the ‘without target compounds’ evaluation, we remove all compounds and stereoisomers (first block of the InChI key) present in the target dataset from the training data, again on a per-dataset basis. The ‘maximum challenge’ evaluation combines both scenarios, removing similar datasets as well as compounds and stereoisomers of the test datasets.

### Curation of retention order statistics data

To avoid that our somewhat subjective estimates on dataset quality impedes the statistical analysis of the data, we decided to perform this analysis without discarding the abovementioned datasets from the RepoRT repository. We only discarded datasets from our analysis when relevant metadata were missing^46^, including datasets with missing annotation for the void volume (Supplementary Table 1).

Column names in RepoRT were standardized^34^, to avoid that one column may be stored under different names. Columns are characterized according to their ligand using the USP classification scheme. For example, the widely used C18 columns are classified as L1 in USP coding. Notably, two columns with the same USP code may show substantial differences in selectivity, for example due to differences in base particle material, end-capping or silanol activity. Column names as well as USP codes are known for 290 (97.6%) datasets.

HSM and Tanaka parameters are known for 78.5% and 76.7% of the datasets, respectively. For 223 datasets (75.1%) both HSM and Tanaka parameters are available. For Tanaka parameters, we ignore particle size, as the difference in parameters is negligible^34^. There are 113 unique combinations of column Tanaka values and 126 unique combinations for HSM parameters. The pH of the mobile phase is annotated for 91.9% of the datasets. In RepoRT, components of the mobile phase alongside their concentration are annotated for all except one dataset (99.7%). In our analysis, we focused only on the components and ignored concentrations.

Temperature influences the thermodynamics of the interaction of compounds with the stationary and mobile phase and therefore, the retention time. As this effect is dependent on the compound, retention order may change with temperature. Temperature is annotated for 61.3% of the datasets. Temperature-dependent changes in retention order are substantially harder to monitor than those of stationary and mobile phases. To this end, we concentrated on ten clusters of datasets where only the column temperature was varied. For each cluster, we considered only compound pairs informatory between all dataset pairs. For clusters with multiple datasets per column temperature, we excluded compound pairs with different retention orders for equal conditions. Finally, for the resulting ten clusters of a total of 70 datasets, we removed seven datasets that substantially reduced the number of informatory compound pairs (0255, 0317, 0325, 0326, 0341, 0342 and 0357).

### Architecture and training of the retention order index prediction models

We use a modified directed message-passing neural network (D-MPNN)^35,36^ to predict retention order indices. For encoding the compounds from their molecular graphs to an embedding of size 512 we use the D-MPNN implementation included in the ‘chemprop’ Python package. The default atom and bond features are used for initializing atom and bond embeddings, namely atomic number, atomic mass, number of neighboring hydrogen atoms, number of connected bonds, formal charge, chirality, aromaticity and hybridization for atoms and bond type, bond stereochemistry, and whether it is part of a ring of bonds. Hydrogen atoms are encoded implicitly via the features of neighboring atoms. After embedding these initial features using the same embedding size of 512, a fixed number of message-passing steps is performed, updating the embeddings of atoms and bonds each time with information from neighboring atoms and bonds. We set the number of message-passing steps to 3. The final compound embedding is obtained by averaging over all atom embeddings. Standardized parameters of the chromatographic setup encoded by a 13-dimensional vector are concatenated to the molecule embedding. This vector contains the pH of the mobile phase as a single real-valued number and 12 values for the stationary phase: six HSM parameters (hydrophobic interaction, steric interaction, column hydrogen bond acidity, column hydrogen bond basicity, column cation exchange activity at pH 7.0 and pH 2.8) and six Tanaka parameters (hydrophobicity, hydrophobic selectivity, steric selectivity, hydrogen bonding capacity, ion exchange capacity at pH 7.6 and at pH 2.7)^37,38^. From the concatenation of molecule and system embedding, an embedding describing the relationship between chromatographic setup and molecular structure is learned, using two feed-forward layers of size 256 each. After two additional feed-forward layers for dimensionality reduction with sizes of 256 and 64, respectively, a single neuron is the output for the ROI. For all layers except this last one, a rectified linear unit activation function is employed. For training the model, we use a Siamese setup, where two instances of our model with identical weights predict the retention order indices of the two compounds.

To determine the number of message-passing steps of the D-MPNN, the embedding size and the number of layers and neurons in the feed-forward layers, we performed a grid search on a hold-out portion of 32 RepoRT datasets in fivefold cross-validation (Supplementary Table 8). Observing robust performance for all configurations, we reintegrated these datasets into the training and evaluation data.

Models that use either no or a subset of chromatographic features, employ essentially the same architecture, with the exception that the concatenation step is missing. Doing so, we ensure that all models have basically the same number of learnable parameters, being 950,913 for models with, and 947,585 for models without chromatographic features.

Training data were pre-processed as follows. For each dataset, we kept those compound pairs for which at most one compound elutes in the void volume. Doublets (compounds with more than one retention time annotated per dataset), were not filtered out. Instead, the retention time range was used to filter out invalid compound pairs, removing for each doublet all pairs inside that range. We assigned weights to all compound pairs, determined by the difference in retention time. Doing so, pairs with a small difference in elution time and, hence, large uncertainty about the retention order have a smaller influence in training than those pairs where the retention order is clearer. We compute the weight using a sigmoid function

$$f(x)=\frac{1}{1+\exp \left(-20(x-0.75)\right)}$$

where *x* is the retention time difference in minutes. In practice, this results in pairs with up to 30-s retention time difference being weighed down substantially, and pairs with more than 60-s difference receiving basically identical (unit) weight. Furthermore, to account for the different sizes of datasets, we sampled compound pairs in each epoch by uniformly drawing a dataset, then uniformly drawing a compound pair from this dataset. In each training epoch, 500,000 compound pairs were sampled.

The model was trained for a maximum of ten epochs using the Adam optimizer^53^ with a fixed learning rate of 5 × 10^−4^ and a batch size of 512; a margin ranking loss with margin 0.1 was employed. We used this greater-than-zero margin for faster convergence, as correct predictions receive a loss ‘boost’ equal to the margin. The value of the margin was again established using a hyperparameter search (Supplementary Table 8). The model was saved after every epoch, and the model with the highest pair accuracy on the training set was used for evaluation. We use the manually determined number of ten epochs (maximum) for all models to counteract overfitting observed for high numbers of epochs (Supplementary Fig. 10). We used PyTorch^54^ for our implementation.

### Evaluation of retention order prediction

To evaluate how well the ROI model can transfer between setups, we used two different dataset splits in fivefold cross-validation. For the regular split, no dataset from the train part shares a (nominally) identical chromatographic setup with a dataset in the test part. For the challenging dataset split, this extends to the combinations of chromatographic column and estimated pH value: A dataset from the train part must not have the same column and a similar pH as a dataset in the test part. Here, we considered a pH difference of less than 1 to be ‘similar pH’. Recall that we considered only those compound pairs with retention time difference of at least 10 s in our evaluation, and that we ignore compound pairs for which both compounds elute in the void volume. Evaluation statistics are reported on average per dataset, as global statistics are dominated by the largest datasets: Namely, dataset 0390 with 7,333 compounds and dataset 0391 with 9,686 compounds. We report error rates, being one minus accuracy.

For comparing the performance of models trained with and without features for the chromatographic conditions, we focus on setup-characteristic pairs: For each compound pair, a consensus order was determined, being the retention order observed in the majority of datasets. Setup-characteristic pairs are pairs that deviate from the consensus order. As noted in the results, some compound pairs exhibit a different retention order in datasets with identical HSM parameters, Tanaka parameters, and pH value (0.70% of compound pairs, dataset average). It is important to understand that the majority of these compound pairs are not contradictory per se, but only because we decided to consider only column and pH in our model. The majority of such ‘contradictory’ pairs (86.5%) occur between datasets from the three biggest dataset clusters in the RepoRT training data. In these clusters, chromatographic parameters such as chromatographic temperature are comprehensively varied. We often observe ‘contradictory’ pairs when either temperature, flow rate and gradient are varied, or when the mobile phase composition is not identical, but the estimated pH is the same. For about a third (29.1%) of the contradictory pairs, the chromatographic setup is nominally identical. This might indicate chromatographic parameters beyond the ones considered here to be important for retention order, or incorrect compound annotations (Supplementary Figs. 1 and 2 show distributions of the different types of compound pairs for datasets of the two cross-validation scenarios). For evaluations on setup-characteristic pairs, we report accuracies instead of error rates.

We projected the training time of the RankSVM from ref. ^27^ to exceed one month for all datasets. To this end, we performed a fivefold cross-validation on 100 randomly selected datasets, explicitly excluding the two largest datasets 0390 and 0391 beforehand. For these 100 datasets, we evaluated the RankSVM performance using fivefold cross-validation; for all remaining datasets in RepoRT, we used a RankSVM trained on all 100 datasets. RankSVM training was performed as described for the scenario ‘Multiple systems, no target data’ in ref. ^27^ using the MinMax kernel and counting Molecular ACCess Systems fingerprints. Preparation of training data, including doublet handling and removal of void compounds, was conducted as described elsewhere^27^, namely removing compounds in the void volume and doublets with more than 5% relative difference in retention time. To further decrease running time, parameters ‘n_rep’ (internal repetitions) and ‘n_splits_cv’ (folds) were set to 1; compound structures in the test datasets were not removed from training data (parameter ‘excl_mol_by_struct_only’ equals ‘False’).

In addition to RankSVM, we trained and evaluated another setup-agnostic model for retention order prediction, using molecular descriptors calculated by RDKit (RDKit: open-source cheminformatics; https://www.rdkit.org) as features for compounds. We refer to this model as RankNet, it being a classical implementation of the RankNet idea^39^. We initially computed all 218 descriptors available in RDKit v.2022.09.5 (https://doi.org/10.5281/zenodo.7671152) and discarded descriptors with invalid values for at least one compound and descriptors with practically the same value for all compounds, using an s.d. cutoff of 0.01. For all models trained, 203–209 descriptors were used, depending on the cross-validation fold. We used one hidden layer of size 256, followed by the output neuron representing the ROI. Except for the output, we employed the rectified linear unit activation function. The model has approximately 70,000 learnable parameters and is trained exactly as our ROI prediction model, with batch size 512, learning rate 5 × 10^−4^, and a margin ranking loss with margin 0.1. We tested three configurations for the number of hidden layers and their size: one layer of size 16, one layer of size 256, and two layers with sizes 256 and 64. This is analogous to the grid search above, as the other hyperparameters explored there are not applicable here; the values tested for the hidden layers are the same. We selected the second one as best performing, using the same hold-out portion of 32 RepoRT datasets in fivefold cross-validation as used for the grid search above.

### Mapping retention order indices to retention times

For each molecular structure from the set of anchors, we used the GNN to predict a ROI using the specified chromatographic parameters. To deal with falsely annotated anchors, we established the mapping in two steps. In the first step, we computed an initial mapping using LAD (median regression) for a polynomial of degree two. The coefficients were constrained to be non-negative, ensuring a monotonously increasing fitting function. Second, we discarded all anchors where the error was more than twice the median error. Third, we computed a refined mapping by calculating the coefficients via OLS. If the refined mapping was not monotonously increasing, the LAD mapping was used instead. The initial constrained median regression was implemented as a Linear Program using the ‘pulp’ Python package as a generic solver interface. For OLS, the ‘statsmodels’ implementation was used^55^.

For comparison, we used three methods for computing robust fits, namely GAM, SCAM and COBS. We used the R package mgcv to compute GAM fits with univariate P-splines as basis, with otherwise default values of the ‘gam’ function.^40^. For SCAM, we used the scam R package and its function of the same name, constraining the fit to be monotone increasing, with otherwise default parameters^56^. For COBS models, we used the R package cobs and its function of the same name. Here again, we constrained fits to be monotone increasing. We set the penalty parameter lambda to be chosen automatically^57^.

### Evaluation of DeepGCN-RT and RT-Transformer

We evaluated our 2-step method against the best-in-class methods for direct retention time prediction, namely DeepGCN-RT^15^ and RT-Transformer^16^. Both methods follow the pretrain/fine-tune paradigm. For our evaluations, we pretrained both models using either the SMRT dataset^41^ used in the original publications, or the substantially larger RepoRT repository. Notably, RepoRT is a diverse collection of many different datasets and, hence, very different from the homogeneous SMRT dataset. For RepoRT datasets, the distribution of compounds follows much more closely that of known biomolecules than this is the case for SMRT^45^. To this end, we employed two different pretraining strategies: For single pretrain, we simply concatenated measurements from all datasets in RepoRT; for repeated pretrain, we repeatedly pretrained on each RepoRT datasets, then used the learned weights from the previous dataset as initial weights for the next one. For pretraining on RepoRT, we again removed compounds in the void volume and doublets with more than 5% relative retention time difference, analogous to the preparation of training data for the RankSVM. A single entry in RepoRT resulted in invalid descriptors for DeepGCN-RT and was removed from training data (RepoRT ID 0391_012923). For fine-tuning, we used the curated versions of the six target datasets without duplicates, doublets or compounds eluting in the void volume, see above. Solely for the training portions in cross-validation, we included compounds eluting in the void volume, observing a gain in performance. Notably, refs. ^16^ and ^15^ used noncurated datasets for fine-tuning. We used the code provided by the authors for training the models. Different from what is described elsewhere^16^, we found that the DeepGCN-RT code wrongly selects the model for which statistics are reported based on the test split instead of the validation split; we fixed this bug. For pretraining RT-Transformer on the SMRT dataset, we used the full SMRT dataset including compounds in the void volume, as ref. ^15^ reports better fine-tuning results for this variant. Notably, the RT-Transformer source code did not include a method for fine-tuning. To this end, we implemented this method anew, following the description in the publication. Evaluation results were obtained using tenfold cross-validation on the target datasets. Our evaluation results using SMRT for pretraining were similar to those reported elsewhere^15,16^. We observe a slight drop in performance, presumably due to fixing bugs in the code and in the evaluation.

### Evaluation statistics

For retention order we report the accuracy (fraction of correct pairs among all pairs) or the error rate (fraction of incorrect pairs among all pairs). For retention times we report the mean absolute error (MAE) of all predictions *y*_*i*_, being $1/n\cdot {\sum }_{i}\left\vert {x}_{i}-{y}_{i}\right\vert$ where *x*_*i*_ are the observed retention times, *i* = 1, …, *n*. Before computing errors, we normalize all retention times to the length of the gradient of the individual dataset. Normalizing by gradient length is more reasonable than reporting mean absolute percentage error or mean relative error. An error of 60 s is equally important at the beginning and at the end of the run.

Notably, the folds of the realistic split do not all have the same size. Consequently, the MAE of the dataset is usually not the mean of the mean absolute errors of the individual folds. Instead, we have to use the weighted mean to derive the MAE of the dataset from the MAE_*k*_ (MAE of fold *k*),

$${MAE}=\frac{1}{n}\cdot \mathop{\sum }\limits_{k=1}^{10}{w}_{k}\cdot {{MAE}}_{k}$$

for our tenfold cross-validation, where *w*_*k*_ is the number of samples in fold *k*, and ∑_*k*_*w*_*k*_ = *n*. This insight may seem superfluous, as we can directly calculate the MAE of the dataset, ignoring the folds. Yet, it becomes important when we want to report sample variance and s.d. over folds. Here, reporting the unweighted variant is highly misleading; instead, we must also compute the weighted variance as

$${s}^{2}=\frac{1}{n-1}\cdot \mathop{\sum }\limits_{k=1}^{10}{w}_{k}{\left({{MAE}}_{k}-{MAE}\right)}^{2}.$$

Note the Bessel’s correction for frequency weights to reach an unbiased estimator. We report *s* as an unbiased estimator of the s.d. over the folds.

For the coefficient of determination, *R*^2^, it is important to not use an average of the folds. Rather, *R*^2^ is computed for the complete dataset as

$${R}^{2}=1-\frac{{S}_{{\rm{res}}}}{{S}_{{\rm{tot}}}}\quad \,\mathrm{where}\,\quad {S}_{{\rm{res}}}=\mathop{\sum }\limits_{i=1}^{n}{({x}_{i}-{y}_{i})}^{2}\quad \,\mathrm{and}\,\quad {S}_{{\rm{tot}}}=\mathop{\sum }\limits_{i=1}^{n}{({x}_{i}-\bar{x})}^{2}\,.$$

Here, $\bar{x}=1/n\cdot {\sum }_{i}{x}_{i}$ is the mean of the observed retention times. In this form, *R*^2^ measures the goodness of fit; notably, *R*^2^ may be negative, in case the mean of the measured retention times is a better prediction for the observed retention times than those made by the predictor. In this case, the actual value is of no importance, as the predictor is basically useless.

### Analysis of fecal sample dataset

A public human fecal dataset from MetaboLights (accession MTBLS8356) was analyzed to showcase the impact of transferable retention time prediction. Human fecal samples purchased from BioIVT (Burgess Hill) were used for the fecal metabolome extraction. Homogenized samples were extracted as described previously^58^. Nontargeted analysis of extracts was performed using Sciex ZenoTOF 7600 with a Sciex ExionAD UHPLC as described elsewhere^59^. In brief, metabolites were separated using a Phenomenex Kinetex C18 column (100 × 2.1 mm ID, 1.7 μm particle size) and a linear gradient for eluent A (100% H_2_O + 0.1% formic acid) to eluent B (100% ACN + 0.1% formic acid) at a flow rate of 0.5 ml min^−1^ and a column temperature of 40^ ∘^C. *N*-alkylpyridinium 3-sulfonate (NAPS) authentic standards (Extended Data Fig. 3) and bile acid authentic standards were measured in independent samples under identical conditions. Processing of LC–MS data was performed using MZmine v.4.7.8, which included peak-picking, alignment and adduct grouping^60^. MS/MS data were exported to .mgf files, which were then imported into SIRIUS.

We performed spectral library search, and used our 2-step approach to predict retention times for all molecular structures in the spectral library. For ROI prediction, a model was trained on all 171 datasets of the RepoRT training data. The mapping from ROI to retention time was established using 19 NAPS molecules as anchors, which were measured in a separate run. This demonstrates that the mapping can be established using a very small number of anchors, and that anchors do not have to come from diverse compound classes for a robust prediction of retention times. We searched reference libraries of MassBank^49^, MassBank of North America (https://mona.fiehnlab.ucdavis.edu/), GNPS^61^ and MSnLib^62^. Spectral library search was performed using SIRIUS (v.6.4.0)^63^. In detail, precursor ions were removed before matching, and square root intensities were used before computing the cosine similarity (dot product of the normalized spectra). For in silico annotations using CSI:FingerID, we ran SIRIUS (v.6.4.0) with default settings for molecular formula identification and structure database search.

Using spectral library search, we found 68 query spectra for which the cosine similarity to a spectrum in the library was greater than 0.7, at least four peaks were shared between query and reference spectrum, and there exist no other spectra in the spectral library that met these criteria. Of these, nine compounds had an experimental retention time matching the predicted retention time within 0.40 min, making these ‘almost level 1’ identifications (Supplementary Table 6). The retention time threshold corresponds to the MAE from cross-validation on the anchor compounds. We did not posses authentic standards for these compounds and, consequently, were unable to verify the correctness of these annotations; for the same reasons, we were unable to verify which of the annotated compounds with larger retention time deviation are incorrect. To this end, we concentrated on the two hits with cosine above 0.9. These hits correspond to compounds 1,7-dimethyluric acid (PubChem ID 91611) and dibutyl phthalate (PubChem ID 3026). Both hits are biochemically and experimentally reasonable: 1,7-dimethyluric acid is a known metabolite of caffeine found previously in feces^64^ and dibutyl phthalate has been found even in human blood^65^. Two additional hits to putative lipids were discarded, as the exact molecular structure cannot be elucidated using collision-induced dissociation fragmentation. See Extended Data Fig. 6b–e.

Next, we found two query spectra with MassBank library matches to both 3-ketocholanic acid and lithocholenic acid, with cosine values between 0.73 and 0.80. These compounds share the same mass and differ only in the position of a double bond, which is either located in the C-ring or as keto instead of a hydroxy group at the A-ring (Extended Data Fig. 6f). Notably, the retention time of these two features differs substantially at 6.65 min and 7.09 min, respectively. The predicted retention times for 3-ketocholanic acid is 6.89 min and for lithocholenic acid 7.15 min. Consequently, one feature matches retention time closely with that of 3-ketocholanic acid, and one feature with that of lithocholenic acid. These are clearly no unambiguous identifications, but showcase how predicted retention times allow us to decide which feature is which compound (Extended Data Fig. 6f).

Third, we found 16 query spectra with spectral library matches to the bile acid cholic acid, ranging from 5.50 min to 7.81 min (Extended Data Fig. 6g). Although we also found one additional unambiguous spectral match (only matching to cholic acid and no other compound), this match was discarded because the detected ammonium adduct is unreasonable for the experimental settings. The predicted retention time of cholic acid is 6.09 min. Four of the 16 ambiguous spectral matches fall within the predicted retention time range. The feature with retention time 6.25 min was confirmed via an internal standard to be either cholic acid or allocholic acid; differentiating between these two compounds was not possible with the employed chromatographic method, see below (Extended Data Fig. 6g).

Fourth, we measured authentic standards from three bile acids (cholic acid, allocholic acid and ursocholic acid) using the same experimental setup as above. Notably, these compounds are diastereomers with identical connectivity (two-dimensional structure). Cholic acid (6.14 min) and allocholic acid (6.10 min) have highly similar experimental retention times, whereas ursocholic acid elutes earlier (5.41 min). Notably, the predicted retention times (6.09 min for cholic acid, 6.05 min for allocholic acid and 5.71 min for ursocholic acid) closely match the experimental values (Extended Data Fig. 6h).

Finally, Supplementary Fig. 6 shows how 2-step retention time prediction can be used together with the in silico method CSI:FingerID for structure elucidation, allowing filtering and reranking of candidate lists. In particular, we see how 2-step retention time prediction allows us to select the best annotation for this query, improving both upon the pure in silico annotation and the spectral library search.

### Measures of disagreement

Recall that a conflicting pair consists of two compounds A, B that elute in different order (A before B versus B before A) in two datasets. Notably, conflicting pairs can only be determined on the basis of pairs of datasets. A compound pair consists of two compounds A and B that are both part of two datasets. A more precise denomination for a ‘compound pair’ would be an appearance of a compound pair in a dataset pair. We used the shorter term ‘compound pair’ for the sake of readability. Now, any compound pair is either in conflict (a conflicting pair) or not in conflict, according to the two datasets it appears in.

We evaluated conflicting pairs at different retention time thresholds. It is understood that the higher the retention time threshold, the less likely it is that two compounds are in conflict. A threshold of 60 s implies compound A appears at least 1 min before B in the first dataset, whereas compound B appears at least 1 min before A in the second dataset. Every such compound pair is also a conflicting pair for retention times thresholds 30 s and 10 s.

A compound pair is monotonous if it is not in conflict for any pair of datasets in the repository. A compound pair is informatory in a dataset if it is not monotonous in the repository, if the difference of elution time is above the threshold, and if at most one of the compounds is in the void volume of the datasets. Given two datasets, we determined the intersection of compound pairs informatory in both datasets. Next, we determined the subset of conflicting pairs from the set of informatory pairs. As a single numerical measure for comparing the retention order of two datasets, we calculated the ratio between these two numbers, resulting in the fraction of conflicting pairs.

Conflicting pairs statistics are based on pairs of compounds; consequently, 5 compounds ideally correspond to 10 informatory pairs, 6 compounds to 15 pairs, and 10 compounds to 45 pairs. When considering the fraction of conflicting pairs, a low number of informatory pairs may result in misleading statistics. If there are only six pairs that are informatory between two datasets, three conflicting pairs caused by the retention time shift of a single compound already result in 50% conflicting pairs. Keeping in mind that wrong annotations of compounds are to be expected, we wanted to choose a threshold *T* to exclude dataset pairs with little information in regard to retention order conflicts. Throughout this paper, a threshold of *T* = 15 was chosen, meaning that dataset pairs with fewer than 15 informatory pairs were excluded from statistics on conflicting pairs, unless stated otherwise.

### Reporting summary

Further information on research design is available in the Nature Portfolio Reporting Summary linked to this article.

## Online content

Any methods, additional references, Nature Portfolio reporting summaries, source data, extended data, supplementary information, acknowledgements, peer review information; details of author contributions and competing interests; and statements of data and code availability are available at https://doi.org/10.1038/s41592-026-03243-2.

## Supplementary information

Supplementary Information Supplementary Notes 1–3, Supplementary Figs. 1–10 and Supplementary Tables 1–8.

Reporting Summary

Peer Review file

Supplementary Table 1 List of RepoRT datasets used for retention order statistics and model evaluation. All datasets from RepoRT are listed, detailing in which evaluation scenario each dataset is used. Information on which datasets are missing important metadata (HSM and Tanaka parameters, pH, void volume estimate, column temperature and flow rate) are also provided. Datasets removed from evaluation following manual curation are specified. This table is provided as a separate file.

## Source data

Source Data Figs. 2, 3 and 5, Extended Data Table 1 and Extended Data Figs. 4, 5 and 7–9. Statistical source data and data points.

## Figures and Tables

**Extended Data Table 1:**
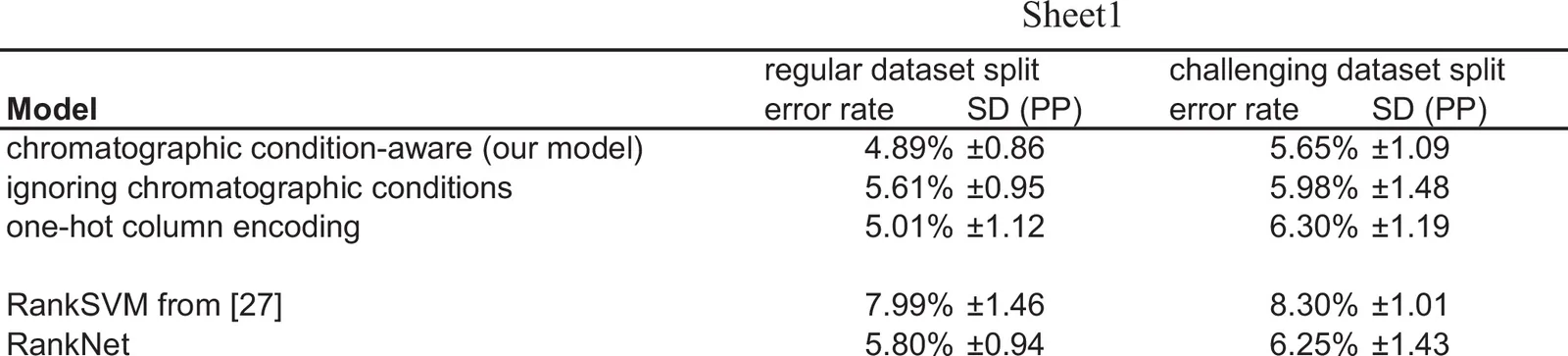
ROI prediction model error rates for regular and challenging dataset splits

*Regular dataset split* is the chromatographic condition-disjoint split described in the results section. For the *challenging dataset split*, test datasets do not share both the same column and a similar pH (difference less than 1) with any training dataset. The RankSVM from ^27^ was trained on 100 randomly selected datasets. Standard deviations (SD) over cross-validation folds in percentage points (PP).

Source data

## Data Availability

Retention time data is freely available from the GitHub repository of RepoRT at https://github.com/michaelwitting/RepoRT. The version used throughout the paper is also available via Zenodo at https://doi.org/10.5281/zenodo.16267767 (ref. ^66^). The fecal sample dataset is available on MetaboLights (accession MTBLS8356). Source data are provided with this paper.

## Code availability

All code is freely available from the dedicated GitHub repository at https://github.com/boecker-lab/2-step and from Zenodo at https://doi.org/10.5281/zenodo.22093097 (ref. ^67^). A web application for 2-step can be accessed from https://2-step.boeckerlab.uni-jena.de.
