# Supplementary material for: Times are changing but order matters: transferable prediction of small-molecule liquid chromatography retention times
Source: Nat Methods. 2026 Oct 1;23(11):2219–29. doi: 10.1038/s41592-026-03243-2 (PMC13736644; doi:10.1038/s41592-026-03243-2)
Supplement: Supplementary file 1 [file 41592_2026_3243_MOESM1_ESM.pdf]

nature methods

Supplementary information

https://doi.org/10.1038/s41592-026-03243-2

# Times are changing but order matters: transferable prediction of small-molecule liquid chromatography retention times

In the format provided by the authors and unedited

## Supplementary Notes

1 Applications of transferable retention time prediction . . . 2
2 Advantages of our 2-step ROI/map approach over pretrain/fine-tune . . . 5
3 How considering pairs of compounds results in small percentages . . . 8

## List of Figures

1 Composition of compound pairs types in the regular evaluation split . . . 9
2 Composition of compound pairs types in the challenging dataset split . . . 10
3 Mapping from Retention Order Indices to retention time for novel columns . . . 11
4 Mapping from Retention Order Indices to retention time improves for the chromatographic condition-aware model . . . 11
5 DeepGCN-RT performance does not improve with more pretraining data . . . 12
6 2-step retention time prediction combined with CSI:FingerID . . . 13
7 Conflicting pairs for datasets with nominally identical or different chromatographic setup . . 14
8 Influence of temperature on conflicting pairs . . . 15
9 Influence of column flow rate on conflicting pairs . . . 16
10 Training epochs for the ROI prediction model. . . . 17

## List of Tables

1 List of RepoRT datasets used for retention order statistics and model evaluation . . . 18
2 Comparison of retention time prediction performance for the uniform split . . . 18
3 Extended comparison of retention time prediction performance . . . 19
4 Ablation studies for retention time prediction performance . . . 20
5 Non-normalized retention time prediction errors . . . 20
6 Spectral library search combined with 2-step leads to nine “almost level 1” identifications . . 21
7 Conflicting pairs statistics . . . 21
8 Hyperparameters for the ROI prediction model explored in grid search . . . 22

## Supplementary Note 1

## Applications of transferable retention time prediction

As demonstrated by thousands of publications, prediction of retention time is generally considered of enormous importance. A machine learning model not restricted to a particular chromatographic condition or a particular set of small molecules, would have numerous applications in metabolomics, natural products research, environmental research, exposomics, toxicology, forensic chemistry, pharmacy, and drug degradation research, as well as all other areas of life science and biochemistry that use liquid chromatography-tandem mass spectrometry (LC-MS/MS) for the annotation and identification of small molecules. In the following, we name a few examples:

- **Boosting spectral library search.** The most obvious application of transferable retention time prediction is to boost the results of a spectral library search: Small molecules in a LC-MS/MS run can be annotated by searching in one or multiple spectral libraries, where MS/MS spectra are compared to those from authentic standards. Yet, retention times are only available when searching in-house libraries, where all compounds to be identified where measured upfront as authentic standards on the same experimental platform. Unfortunately, such in-house libraries are expensive and work-intense to generate, and nevertheless one or more orders of magnitude smaller than public spectral libraries such as MassBank[1], GNPS[2] and NIST (National Institute of Standards and Technology) libraries. When searching public libraries, retention times are ignored, as authentic standards were measured using a different chromatographic system, or even without chromatography (direct infusion). Here, we can use predicted retention times of the candidate molecular structures in the spectral library to up- or down-rank candidates, or to exclude certain candidates from consideration simply because the difference between measured and predicted retention time is too high.
- **Transferable retention time prediction enables "almost level 1" identifications when searching public spectral libraries.** According to different guidelines[3,4], high-confidence annotation ("level 1 identification") of a small molecule from spectral library search requires an orthogonal parameter beyond MS/MS. Retention time is such an orthogonal parameter, providing information on the polarity of the small molecule. Transferable retention time prediction allows us to predict the retention times of all compounds in the public spectral library on *our target system*, and would enable identifications with "almost level 1"[3,4]: Retention time prediction are not of the same quality as a measurement from an authentic standard, but a good match between predicted and measured retention time is nevertheless an excellent hint that an annotation is correct. In principle, predictions could also be done using pretrain/fine-tune models. Yet, this requires to measure hundreds of authentic standards on your target system, preferably from diverse compound classes, to curate the training data, and to train and evaluate your own machine learning model, see Supplementary Note 2. To the best of our knowledge, this is rarely done in application.
- **Deconvolution of chimeric spectra.** LC-MS/MS runs are usually measured using data-dependent acquisition (DDA), assuming that separation by mass and retention time is sufficient to ensure that only a single compound is fragmented and measured in every fragmentation spectrum. Yet, for complex biological samples, this assumption is sometimes violated, and *chimeric* spectra of two or more compounds are recorded. Searching in a spectral library, we must now consider all (for the simplest case, cardinality two) combinations of reference spectra with matching precursor mass, to explain the chimeric spectrum. Yet, many precursor masses will result in ten or more candidates from the library. Even when restricting ourselves to pairs of candidates, the number of pairs to consider is *quadratic* in the number of candidates: Ten

candidates already result in 45 pairs of references, and it will be extremely challenging to pinpoint the two reference compounds that best explain the measured spectrum. If we assume — rather conservatively — that transferable retention time prediction allows us to reduce the number of candidates 3-fold, then this would result in a 9-fold decrease of pairs we have to consider. With the above example, if for only 3 out of 10 candidates the predicted retention time matches the measured time, then we also have to consider only $\frac{3 \cdot 2}{2} = 3$ combinations of spectra to explain the chimeric spectrum. Similar considerations hold for the analysis of data-independent acquisition (DIA) analysis of small molecules.

- **Boosting *in silico* searches.** Searching in spectral libraries can only annotate a small fraction of the putative compounds in a LC-MS/MS run[5]. So-called *in silico* methods such as MetFrag[6] and CSI:FingerID[7], allow us to search tandem MS (MS/MS) spectra of small molecules in molecular structure databases, which are orders of magnitude larger than any available spectral library. Clearly, a method for accurate retention time prediction would be of enormous value for *in silico* methods, both to boost search results (down-rank candidate molecular structures with measured retention time deviating from the predicted retention time)[6] and to assign confidence to the best-scoring hit structure[8]. Given the shortcomings of current methods for retention time prediction (Supplementary Note 2), it is not surprising that after 25 years of method development, not a single *in silico* method uses a machine learning model to predict retention time. MetFrag can use measured retention times for annotations since version 2.2, but deliberately uses a very simple "prediction model" based on the (estimated) logP of candidates[6]. Notably, the few studies that use retention time prediction to boost compound annotations report only moderate improvements[6,9–13]. Clearly, for a deep integration of retention time prediction, the method must be capable of predicting retention times for any chromatographic setup; developers of *in silico* methods cannot enforce one particular chromatographic setup and ignore all others, or risk to loose the vast majority (potentially, all) of their users.
- **Generative artificial intelligence models.** *In silico* methods are used to search in an existing database of molecular structures. A recent trend is to replace this database by a generative artificial intelligence model, to generate a set of molecular structures that agree well with the measured MS/MS data[14–17]. Again, a method that can help to rank candidates, or to assign confidence to the best-scoring candidate, would be highly appreciated. A method for transferable retention time prediction could achieve this. We may even want to deeply integrate the retention time model into the model for generating candidates, focusing the generated molecular structures towards those that have the observed retention time.
- **Resolving stereochemistry.** MS/MS is typically not capable of differentiating stereoisomers. Whereas separation of enantiomers requires very specific chromatographic setups that are generally not used in non-targeted analysis, diastereomers and cis/trans-isomers can be separated. Retention time prediction will help to correctly assign different isomeric structures when used in addition to the MS/MS analysis.
- **Re-analyzing public data.** A method for transferable retention time prediction would allow us to also use retention times when reanalyzing datasets[18,19]. Repositories such as GNPS[2] contain hundreds of thousands of LC-MS/MS runs and many millions of small molecule MS/MS[20], of which only a tiny fraction have been structurally annotated. At present, retention times are ignored when re-analyzing these datasets[8,18–20]. Our 2-step method allows to predict retention time for datasets from repositories, too: We need to annotate about 30 compounds with high confidence, to be used as anchors. Beyond that, we need very little information about the chromatographic setup: The model presented here only requires to know the column and mobile phase pH. This can be considered the bare minimum of information about the chromatographic system. Today, many datasets in repositories have incomplete or even missing metadata on the

applied chromatographic setup[21]. Yet, our analysis on conflicting pairs demonstrated that only few chromatographic parameters must be recorded for predicting retention order indices. In fact, the driving force behind this analysis, was to *find a minimum set of chromatographic parameters* that enabled re-analysis and reuse; the fewer metadata have to be entered with a dataset upload, the more likely it is that users will actually do that. We assume that in the near future, these chromatographic parameters will regularly be recorded in repositories, to allow for a re-analysis of the data including retention times.

– **Filtering candidates for MS1 annotations and exact mass search.** Although is generally agreed that high-confidence annotations and identifications must be based on MS/MS spectra plus orthogonal parameters such as retention time, not all features detected in an experiment will have an associated MS/MS spectrum. For these cases, provisional annotations may be made by searching exact $m/z$ values or inferred molecular formulas in structure databases. Obviously, doing so will result numerous incorrect annotations, in particular when searching large structural databases. Retention time prediction can help to substantially reduce the number of candidate structures, e.g., to buy authentic standards that enable the unambiguous identification of a feature.
– **Optimizing chromatographic parameters.** In numerous applications, we know beforehand that we are interested in a list of, say, 500 small molecules. How can we best separate these molecules using liquid chromatography? Here, a method for transferable retention time prediction will allow us to test hundreds of combinations of column, mobile phase and other parameters, to decide which chromatographic parameter will allow for good separation of the molecules of interest. Notably, our proposed method does not predict retention times but retention order indices; yet, a cluttering of ROIs will inevitably also result in numerous compounds with almost identical retention times. Clearly, numerous papers show how to *experimentally* optimize chromatographic parameters for a given set of compounds; our method allows to pre-select promising parameters (including the used columns) for hundreds of parameter combinations, for free.
– **Measuring MS/MS libraries.** Construction of MS/MS libraries is the cornerstone for high-confidence metabolite annotation and identification. Extending existing and measuring new spectral libraries serves the community in two ways: First, more compounds can be annotated in untargeted metabolomics experiments; second, these spectral libraries provide additional training data for machine learning methods. Libraries are generated by measuring authentic chemical reference standards. Using LC-MS/MS to generate a spectral library can substantially simplify this process, as we can pool the authentic standards, which are predicted to have different retention times. This is beyond the current experimental design for such pools, avoiding mixing isomeric and isobaric substances. Notably, we use transferable retention time prediction here as a matter of experimental design, not to analyze a biological dataset. Unfortunately, background signals may overlap with the $m/z$ of a standard, rendering it impossible to use solely $m/z$ to identify it in the LC-MS run. The problem is further aggravated by the existence of different ion forms and adducts of any standard. Clearly, we want to avoid that some $m/z$ is explainable as two different compound adducts or ion forms present in the multiplexed mixture. Here, transferable prediction of retention times will help us to unambiguously decide which feature corresponds to which compound. In fact, we can decide which authentic standards can be pooled together, based on predicted ROI and/or retention times. We can do so before the first experiment is conducted, making it easier, faster and safer (in the sense of fewer mislabeled reference spectra) to generate spectral libraries.

## Supplementary Note 2

## Advantages of our 2-step ROI/map approach over pretrain/fine-tune

To appreciate the advantages of our 2-step ROI/map approach, one has to first understand what is actually required to use any existing models for retention time prediction. Notably, methods without pretraining suffer from the same issues, but perform substantially worse than pretrain/fine-tune methods in practice. First, we have to decide on our chromatographic setup. We have to keep the chromatographic setup fixed throughout the coming years, as changing the setup in any way will make it necessary to start "from scratch". Then, we have to acquire reference compounds; these are usually commercial standards that we have to buy. Existing datasets regularly used for evaluation purposes contain between 80 and 400 compounds; to reach a similar performance, our set of compounds should be of similar size. In practice, even more compounds are needed; for example, some compounds do not ionize in positive/negative mode, or elute in the void volume. Next, these compounds have to be mixed and measured; this is a non-trivial task, as we want to unambiguously identify each compound using its $m/z$ value, see Supplementary Note 1. Finally, all mixtures have to be measured, analyzed, curated and cleaned; misannotations and false retention time values may have a devastating effect on the trained model. Next, we have to train our actual machine learning model. This is not a problem for users skilled in programming or at least scripting, but poses a huge difficulty for those that are not. We mention in passing the problem of *standardizing* molecular structures; using different standardizations can have a strong negative effect on predicted retention times. Notably, both the molecular structures in the training data, and all molecular structures for which we want to predict retention times have to be standardized in the same fashion; from our own experience, we can say that this is a highly non-trivial problem. RT-Pred[22] automatizes the last steps of this workflow, by "outsourcing" the training of the model as well as predictions to a web service. This comes with the usual advantages (say, updates and fixes of the code without user interaction) and disadvantages (say, user has to be online; user is restricted to the model architecture that the web service provider supports; long-term support is unclear). It does not free the user from any of the other burdens mentioned above; for instance, RT-Pred does not offer standardization of molecular structures.

We end up with a model for predicting retention times on our chromatographic system; the model cannot be used by anybody else, since no two chromatographic setups are ever completely identical. (This may explain the huge number of published models for retention time prediction.) The model allows us to predict retention times, but we cannot feed such predictions into, say, a tool for *in silico* compound annotation such as CSI:FingerID. Instead, we will have to export annotation lists from this program, then script a method that combines these results with our own predictions of retention time. In particular, we will not be able to use any existing graphical user interfaces to view the results.

Our approach does not suffer from any of these issues; zero data from the user's chromatographic system is required to train the ROI prediction model. Only to map ROI to RT we need to know any retention times from the target dataset. The performance of our method is on par or better than what has be reported in the literature for pretrain/fine-tune models[23–30]. Yet, these reported performances crucially depend on a uniform cross-validation split of the target dataset. It is well-known that uniform splits overestimate the power of machine learning models[31]. A realistic cross-validation split resulted in substantially worse evaluation statistics for pretrain/fine-tune models than those reported in the literature (Supplementary Fig. 4). In contrast, our model does not require any "refine" data and is fully agnostic of the target dataset. In particular, its performance is independent of the anchor compounds used for establishing the mapping from ROI to RT.

We argue that our 2-step approach has many fundamental advantageous over pretrain/fine-tune methods:

- We detailed above that pretrain/fine-tune methods require a reference dataset measured under identical chromatographic conditions for training the refinement step. Such an *in-house library* can also be used as anchors for the second step of our method. Yet, the existence of an in-house library is in no way obligatory to apply our method. Instead, high-quality annotations from searching in public libraries (annotations that have an excellent cosine score above, say, 0.85, have a sufficiently large number of matching fragments and are in accordance with, say, biological expectations on the sample content) may be used for this purpose. Even under highly pessimistic assumptions[5] we may assume that 30+ compounds can be annotated in this fashion. Our method for establishing the mapping between ROI and RT is very robust to outliers, as it is minimizing the median deviation, and will not be mislead even by a large number of incorrect annotations.
- As few as 15 anchors are sufficient to establish a mapping from ROI to RT (Fig. 3, Supplementary Table 4). In contrast, it is clearly impossible to fine-tune a pretrained model using a target dataset with only 15 compounds. Even assuming that many anchors were wrongly annotated, we argue that 30 putative annotations are sufficient to establish the mapping for all practical considerations. We repeat that the method for establishing the mapping is highly robust to incorrect annotations.
- Recall that the performance of our method is basically independent of the compounds that are used for establishing the mapping. In particular, all of these compounds may belong to the same compounds class, and the in-house library may exclusively consist of, say, amino acids. This will not result in a drop in accuracy for predicting other compounds classes, as the identified compounds are only needed as anchors for mapping ROIs to RTs; the ROI model is not aware of the compounds that were used to establish the mapping. Measuring an arbitrary set of reference molecules to be used as anchors, frees the user from the need to have potentially expensive chemicals showing similarity to the targets interested in, and instead use cheap and readily available substances.

  Furthermore, labs are often interested in certain compound classes, as these are of particular importance for, say, the researched biological system (terpenoids or alkaloids for plants, mycosporine-like amino acids for organisms living in sunny marine environments, and so on). Users can use such "biased" in-house libraries to establish anchors, without fear of performance drop. We have demonstrated this for the fecal dataset (Extended Data Fig. 6 and Supplementary Fig 6) were we used 19 N-Alkylpyridinium 3-sulfonates to establish the mapping. Notably, the authentic standards do not have to be present in the experimental dataset. In contrast, a pretrained model that is fine-tuned using data exclusively from amino acids, will not perform well for other compound classes (Supplementary Fig. 5); it is impossible to fine-tune a model using measurements from 19 N-Alkylpyridinium 3-sulfonates.
- The performance of ROI models will substantially improve in the future, when more training datasets become available. We demonstrated this by using only a fraction of RepoRT to train the model, see Fig. 2d. Predictions particularly benefit from training datasets that have similar chromatographic conditions as the target dataset. A back-of-the-envelope calculation suggests that doubling the size of RepoRT may decrease the ROI prediction error to 2.97 % (regular dataset split). This is clearly overly optimistic, but the trend is also rather obvious. Importantly, this does not require data from "fancy" and expensive authentic standards. Rather, any dataset with a novel combination of chromatographic parameters and compounds will allow the model to improve its performance, even if all compounds are already part of the repository.

Clearly, a similar performance gain must not be expected for pretrain/fine-tune methods, see Supplementary Fig. 5. We observe that RepoRT may already be "too large" for pretraining these method, and conjecture that zero performance gain will be achieved for pretrain/fine-tune methods in the future, no matter how much pretraining data become available.

- Our 2-step approach requires to train a machine learning model only once, which can then be applied to any target dataset. Finding the optimal mapping from ROI to RT is a simple optimization task and can be executed in fractions of a second. In contrast, pretrain/fine-tune will require to execute machine learning, to fine-tune the model for any given target chromatographic conditions. Fine-tuning a pretrain/fine-tune model can be done in a matter of minutes, but will usually require that the user installs and starts a machine learning framework such as PyTorch.
- Our 2-step approach can be used when re-analyzing public data. For none of these datasets, runs from authentic standards are available. Consequently, pretrain/fine-tune and other methods for the direct prediction of retention time are not applicable. To apply the method presented here, basic chromatographic parameters (column and mobile phase, eventually also gradient, temperature, flow rate) must be recorded in the database for the analyzed dataset. At present, this is often not the case; but we have good hope that this will change, as soon as retention time prediction is offering an advantage to the person doing the original analysis. Besides that, we only need the abovementioned 30 high-confidence annotations to establish the mapping.
- Our 2-step approach can be deeply integrated into *in silico* methods for searching molecular structure databases. As there is only one model, this can be shipped with the *in silico* tool or provided, say, as a web service. Not only does this allow us to present predicted retention times in the graphical user interface of the *in silico* tool; even more importantly, it allows us to use deviations between observed and predicted retention time to rerank candidate molecular structure, or to assess the confidence of a hit. We conjecture that all maintained *in silico* tools will integrate transferable retention time prediction via the 2-step approach in the near future. As noted above, this deep integration is practically impossible for pretrain/fine-tune methods.
- Our model for ROI prediction can be fully integrated into other machine learning models, such as generative models for structural elucidation from MS/MS data. In contrast, no such deep integration is possible for pretrain/fine-tune models, as these are trained individually for every target dataset.
- For reproducible science, we must store the model that was used to predict retention times if it is used as part of a publication. This is straightforward for 2-step, but challenging for pretrain/fine-tune, see the RT-Pred web server[22].

## Supplementary Note 3
## How considering pairs of compounds results in small percentages

Here, we will show that considering *pairs of compounds* results in surprisingly small fractions of pairs that have a particular property. We will consider the case that a retention order differs from the consensus retention order; the same arguments hold when computing the fraction of conflicts between two datasets.

Consider a large number of compounds that elute over some gradient length $T$ in the consensus retention order. To simplify our statistic calculations, we assume that compound retention times are uniformly distributed over the full gradient length. Now, consider a dataset for which retention times differ from the consensus: A subset of 10 % of the compounds show a shift of retention time by $+T/10$, whereas all other (90 %) compounds elute at the same time as for the consensus. This is unquestionable noticeable and relevant change in retention times (for a gradient length of 15 min this corresponds to a change of retention time by 90 s for 10 % of the compounds) and also retention order. How many compound pairs have changed, in comparison to the consensus? Ignoring the fact that some compounds now elute later than $T$, we can easily upper-bound this number; the true number is slightly smaller. A compound pair $x, y$ for which $x$ elutes before $y$ in the consensus order, can only change order in case $x$ was shifted and $y$ was not. Shifting the retention time of $x$ by $+T/10$ will change order for 1/10 of the un-shifted compounds $y$. Hence, the pair $x, y$ will swap order with probability $1/10 \cdot 9/10 \cdot 1/10 = 0.009$. In other words, despite the rather dramatic change in retention time and also retention order, we will only observe 0.9 % setup-characteristic pairs for this dataset.

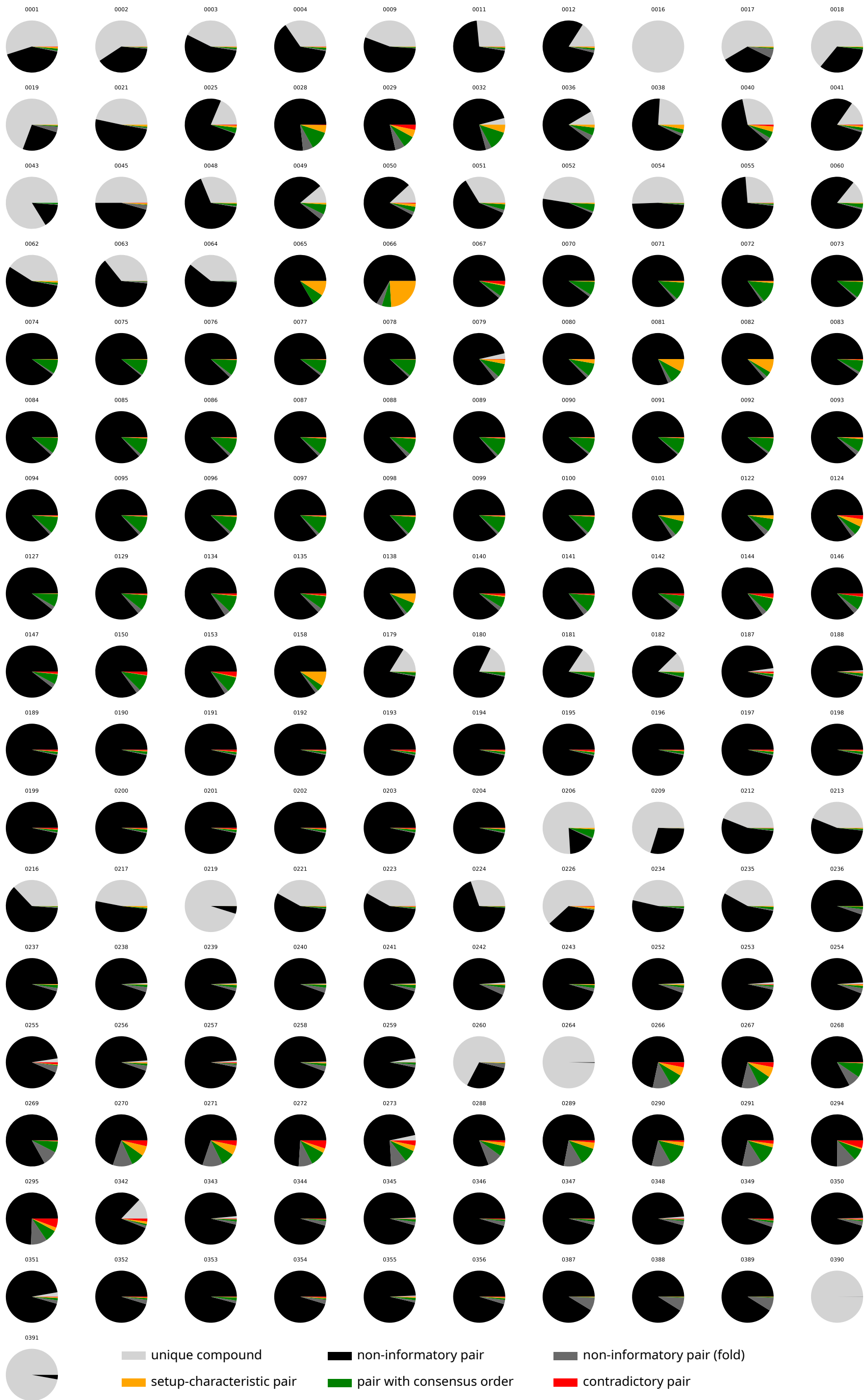


**Supplementary Figure 1. Composition of compound pairs types in the regular evaluation split.** Each dataset is represented as pie chart, showing the proportions of different kinds of compound pairs. Compound pairs labeled "unique compound" have at least one compound that does not occur in any other dataset in this cross-validation fold. "Non-informatory" pairs (see main text for the definition of informatory pairs) are divided between those that are non-informatory only for the respective cross-validation fold, and those non-informatory for all dataset pairs. For each compound pair, a consensus order is determined. The setup is characterized by the HSM parameters, Tanaka parameters, and pH estimations. "Setup-characteristic pairs" are pairs that deviate from the consensus. "Contradictory pairs" are compound pairs where different retention orders are observed for the same features for the chromatographic model (HSM parameters, Tanaka parameters, and pH value).

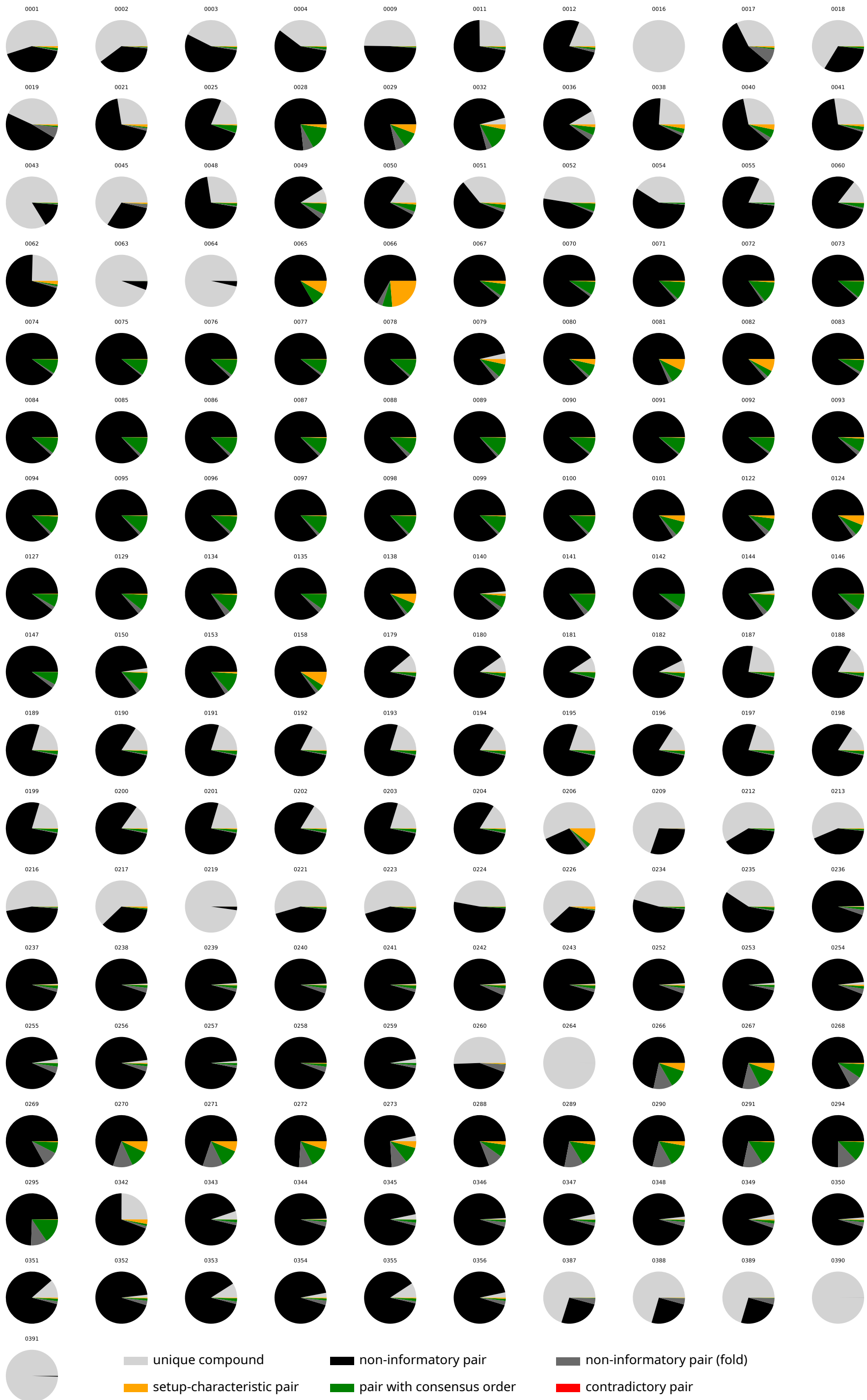


**Supplementary Figure 2. Composition of compound pairs types in the challenging dataset split.** Distribution analysis of compound pair types was repeated for the more challenging dataset split, where test datasets don't share both column and a similar pH with any training dataset. Each dataset is represented as pie chart, showing the proportions of different kinds of compound pairs. For descriptions of the different types, see Supplementary Fig. 1.

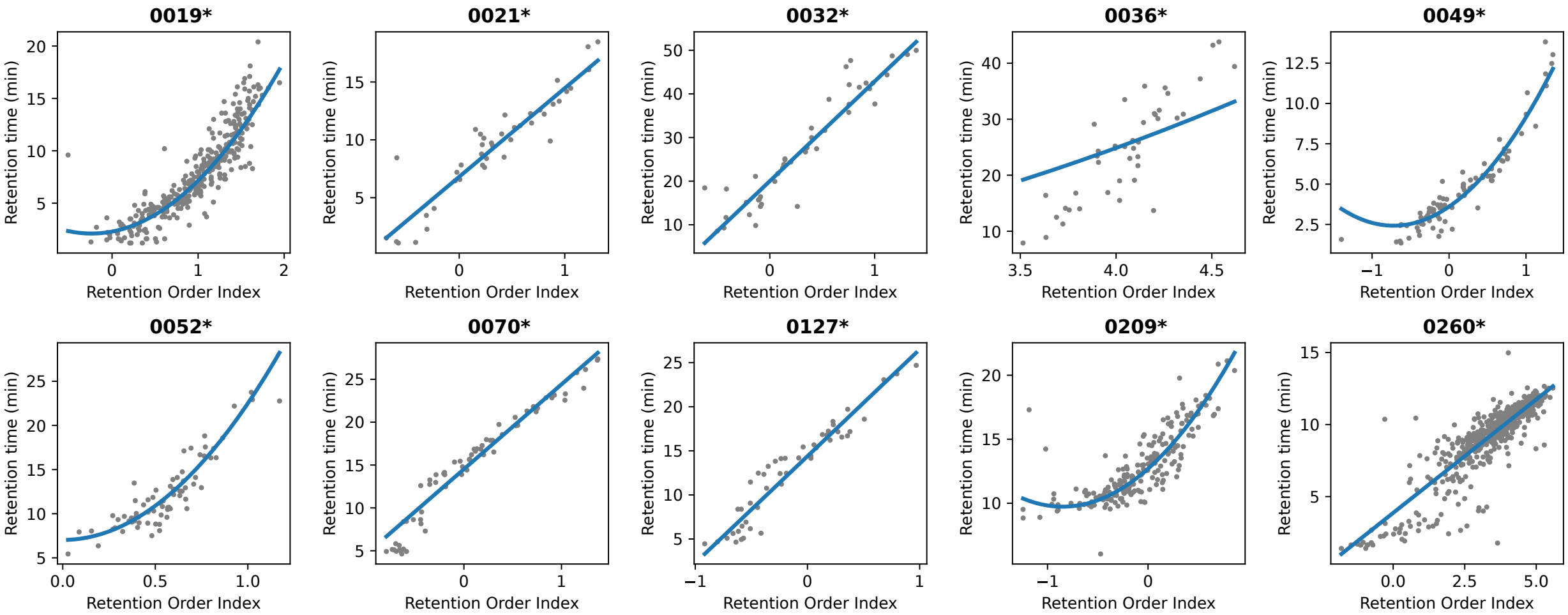


**Supplementary Figure 3. Mapping from Retention Order Indices to retention time for novel columns.** Mappings between ROI and RT are shown for all datasets in the regular cross-validation split, where the employed column was not part of the training data (marked with *). The mapping between ROI and RT was established using 80 % randomly selected data points as anchors. Compounds in the void volume were excluded, for dataset 0209 the void volume threshold was chosen manually.

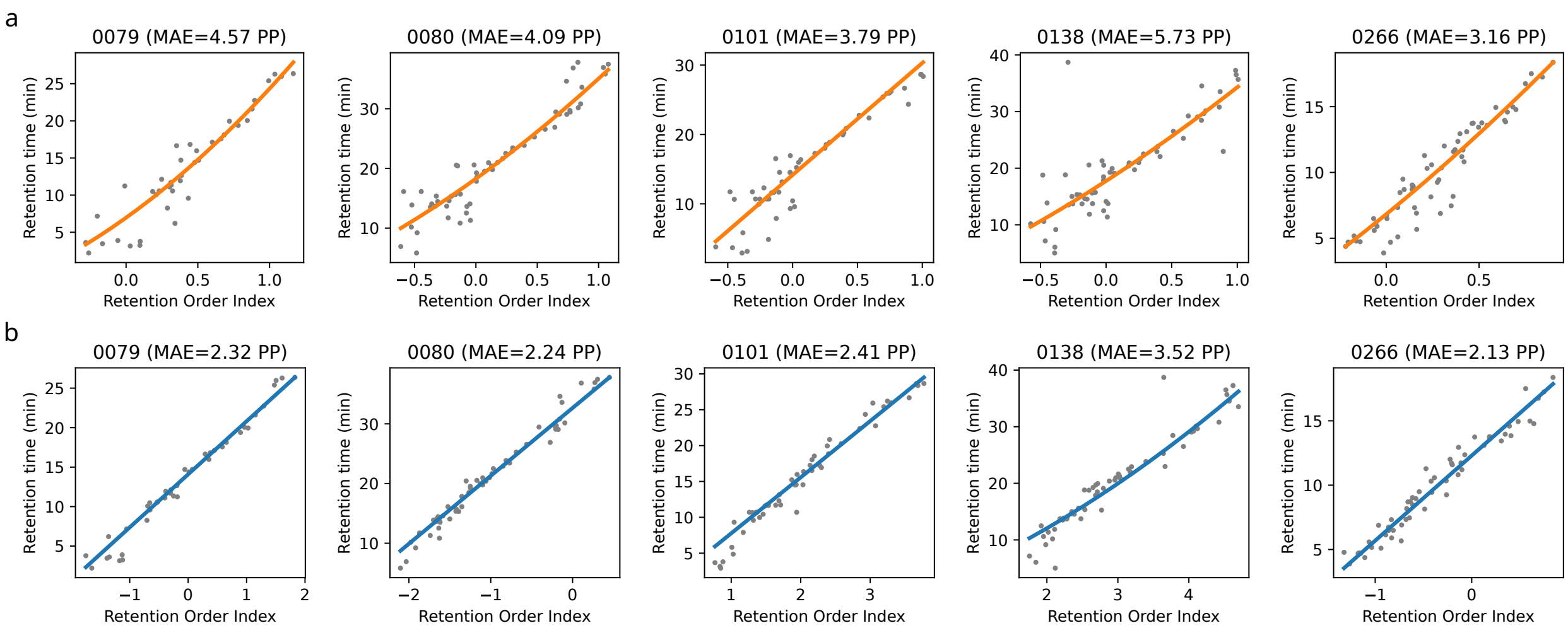


**Supplementary Figure 4. Mapping from Retention Order Indices to retention time improves for the chromatographic condition-aware model.** For five datasets, mappings are shown for the ROI prediction model ignoring chromatographic conditions (a) and the model using these as features (b). Analogous to Extended Data Fig. 4 and Supplementary Fig. 3, mappings between ROI and RT were established using 80 % randomly selected data points as anchors. Mean absolute error (MAE) values using normalized retention times (in percentage points, PP) were estimated for the remaining 20 %. Compounds in the void volume were excluded.

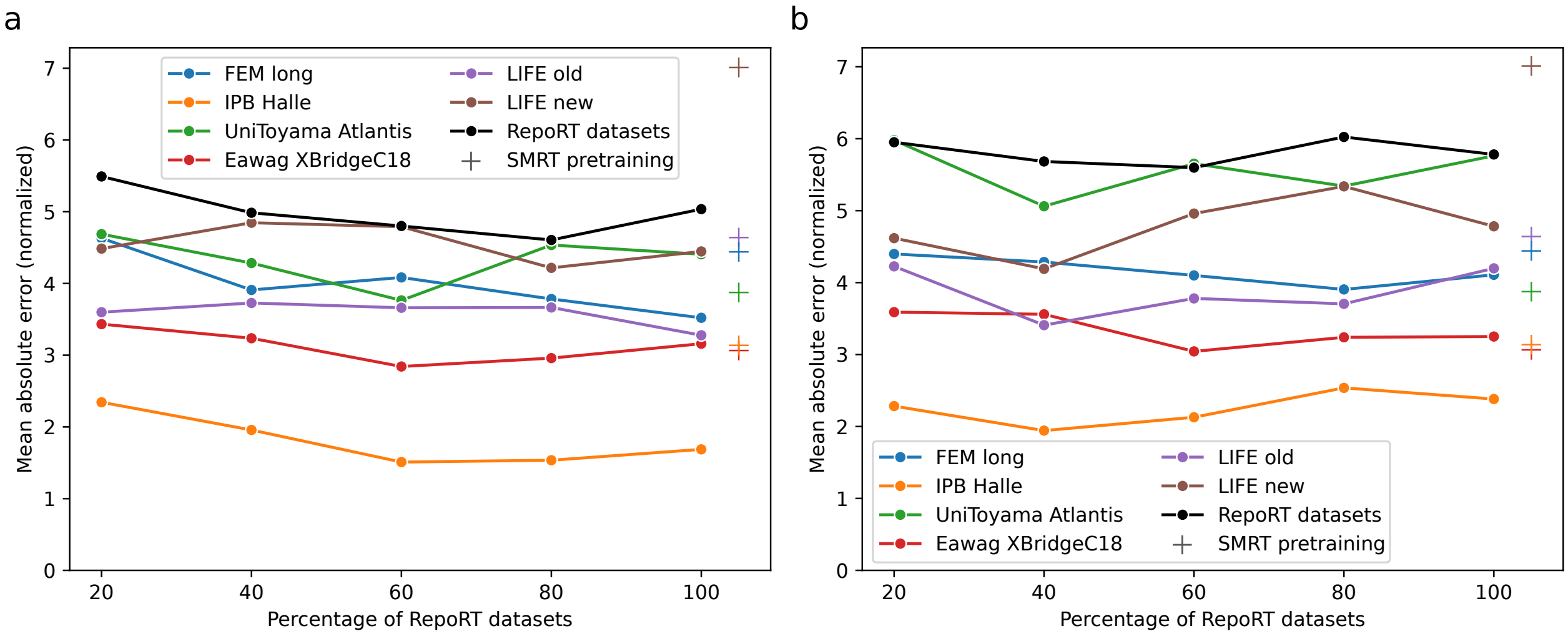


**Supplementary Figure 5. DeepGCN-RT performance does not improve with more pretraining data.** We evaluated the performance of DeepGCN-RT when only a part of the RepoRT data was used for pretraining. Mean absolute errors for the prediction of normalized retention times (in percentage points) are shown, with uniform splitting for fine-tuning. We used the same folds as in Fig. 2. We show results for repeated (a) and single (b) pretraining on RepoRT. We observe no clear trend, as errors increase, decrease or remain constant without any evident patters. Values for pretraining on SMRT are shown for comparison. For 11 out of 35 RepoRT datasets the gradient length was unknown and estimated as the highest retention time measured in the respective dataset in order to normalize retention times.

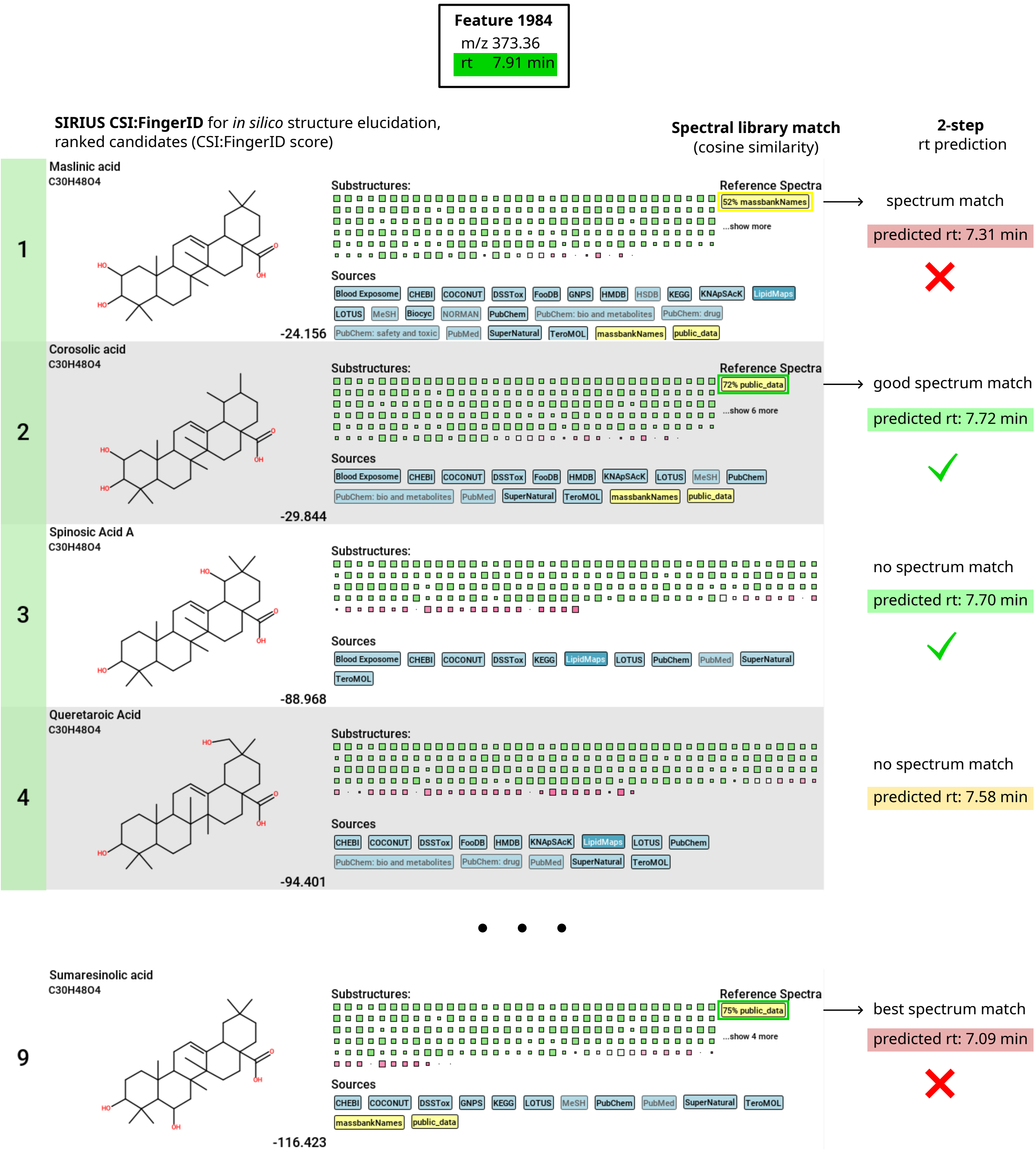


**Supplementary Figure 6. 2-step retention time prediction combined with SIRIUS CSI:FingerID.** For the human fecal sample we used CSI:FingerID for *in silico* structure annotation. In addition, we performed spectral library search in MassBank, MoNA, GNPS and MSnLib. We predicted retention times for structure candidates with our 2-step approach. Notably, for feature 1984 ($m/z$ 473.36, retention time 7.91 min), the spectral library search resulted in an annotation that disagrees with both the CSI:FingerID annotation and the predicted retention time: The best match to a reference spectrum was found for the structure candidate ranked at position 9 (sumaresinolic acid, PubChem ID 12443148, reference spectrum ID CCMSLIB00010112303) with cosine similarity 0.75 (shown in percentage in the figure). Applying our 2-step retention time prediction, the predicted retention time (7.09 min) deviates substantially from the observed value. In contrast, the highest-ranked candidate of CSI:FingerID is maslinic acid (PubChem ID 73659). Here, the predicted retention (7.31 min) also deviated from the observed retention time, albeit not as much. We argue that the best putative annotations are in fact the structure candidates ranked at positions 2 and 3, respectively: Corosolic acid (ID 6918774) and spinosic acid A (ID 16722139) show the best agreement between predicted (7.72 min and 7.70 min) and observed retention time. For corosolic acid, a good spectral match was found as well (cosine 0.72, CCMSLIB00000579782). Notably, the first four shown structure candidates (highlighted in green on the left) have high structural similarity (MCES distance at most 2).

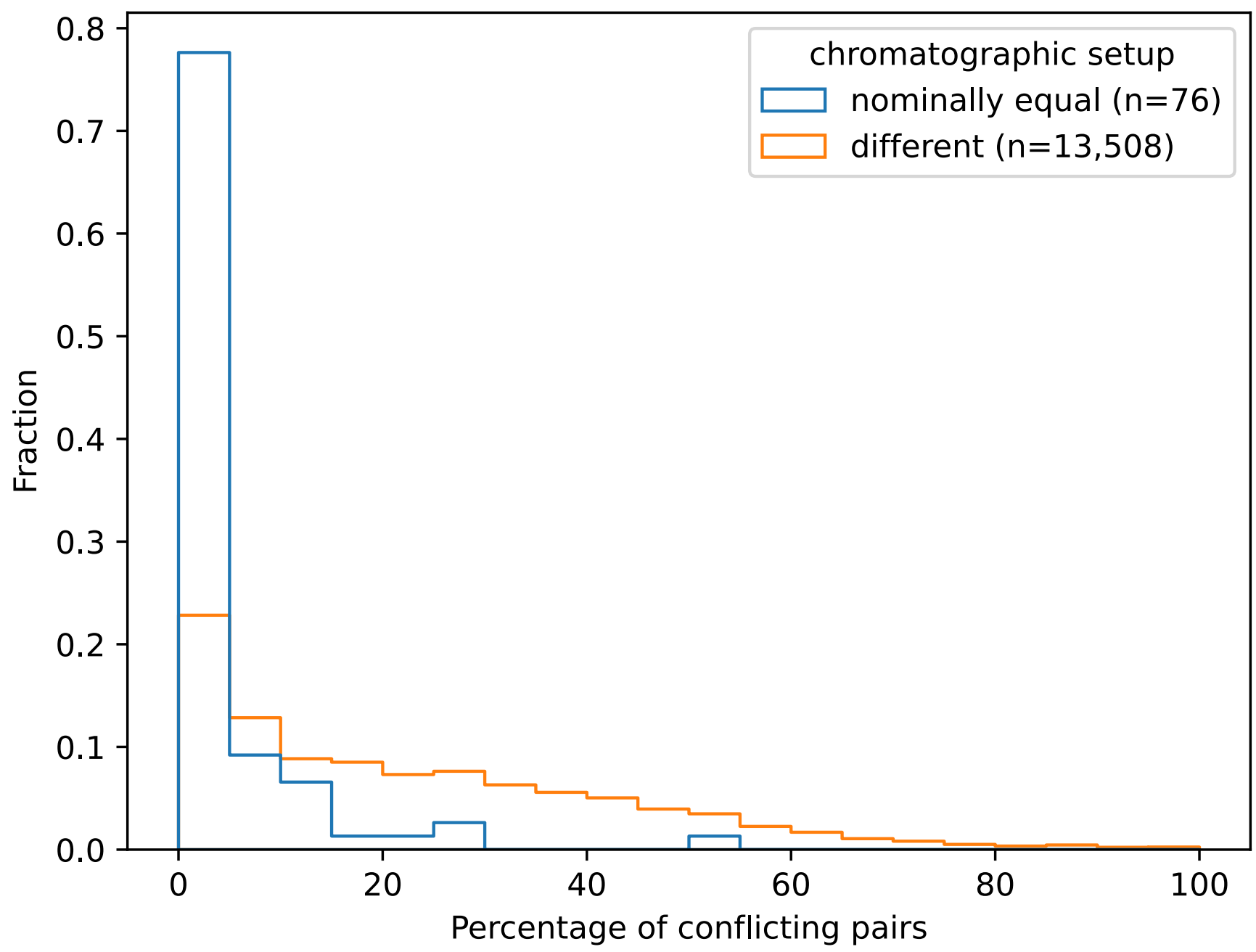


**Supplementary Figure 7. Conflicting pairs for datasets with nominally identical or different chromatographic setup.** Dataset pairs are considered sharing nominally the same chromatographic setup, when the following features are shared, as annotated in RepoRT: Column, flow rate, temperature, column diameter, column particle size, mobile phase composition and gradient. Here, only datasets are considered where all information is annotated. Notably, one pair of datasets with nominally identical chromatographic conditions (0056 and 0057) shows a suspiciously high percentage of conflicting pairs, visible as the outlier at approximately 50 % in the plot. Both datasets were marked as suspicious in RepoRT, and not used for training and evaluation of the methods.

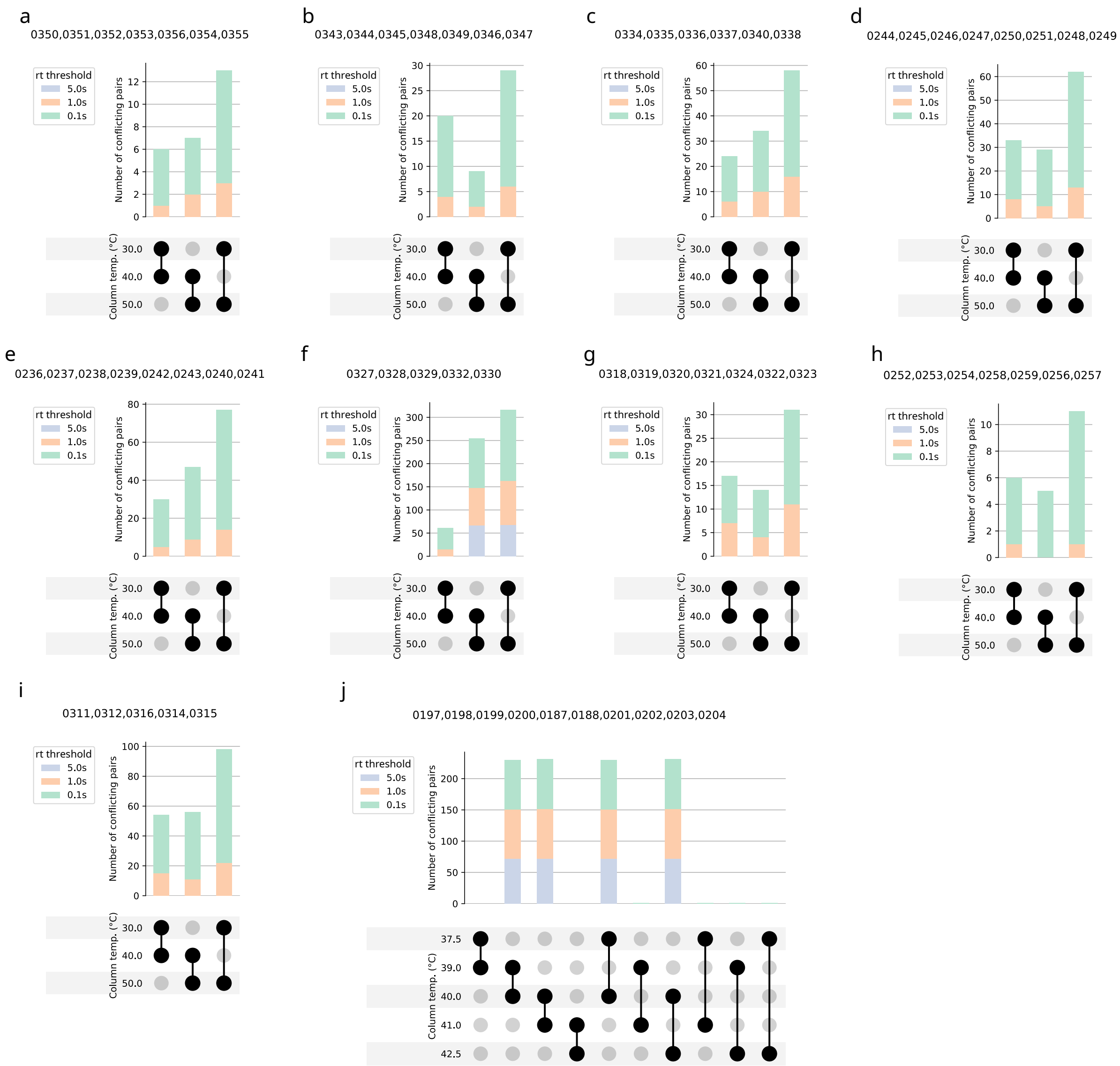


**Supplementary Figure 8. Influence of temperature on conflicting pairs.** Potentially temperature-induced conflicting pairs can only be observed when substantially lowering the time threshold for informatory pairs (RT threshold), as shown for ten examples of dataset clusters (a–j). Here, the absolute number of conflicting pairs is shown for dataset pairs of different temperature settings; note the varying y axis. Titles list the datasets IDs in RepoRT.

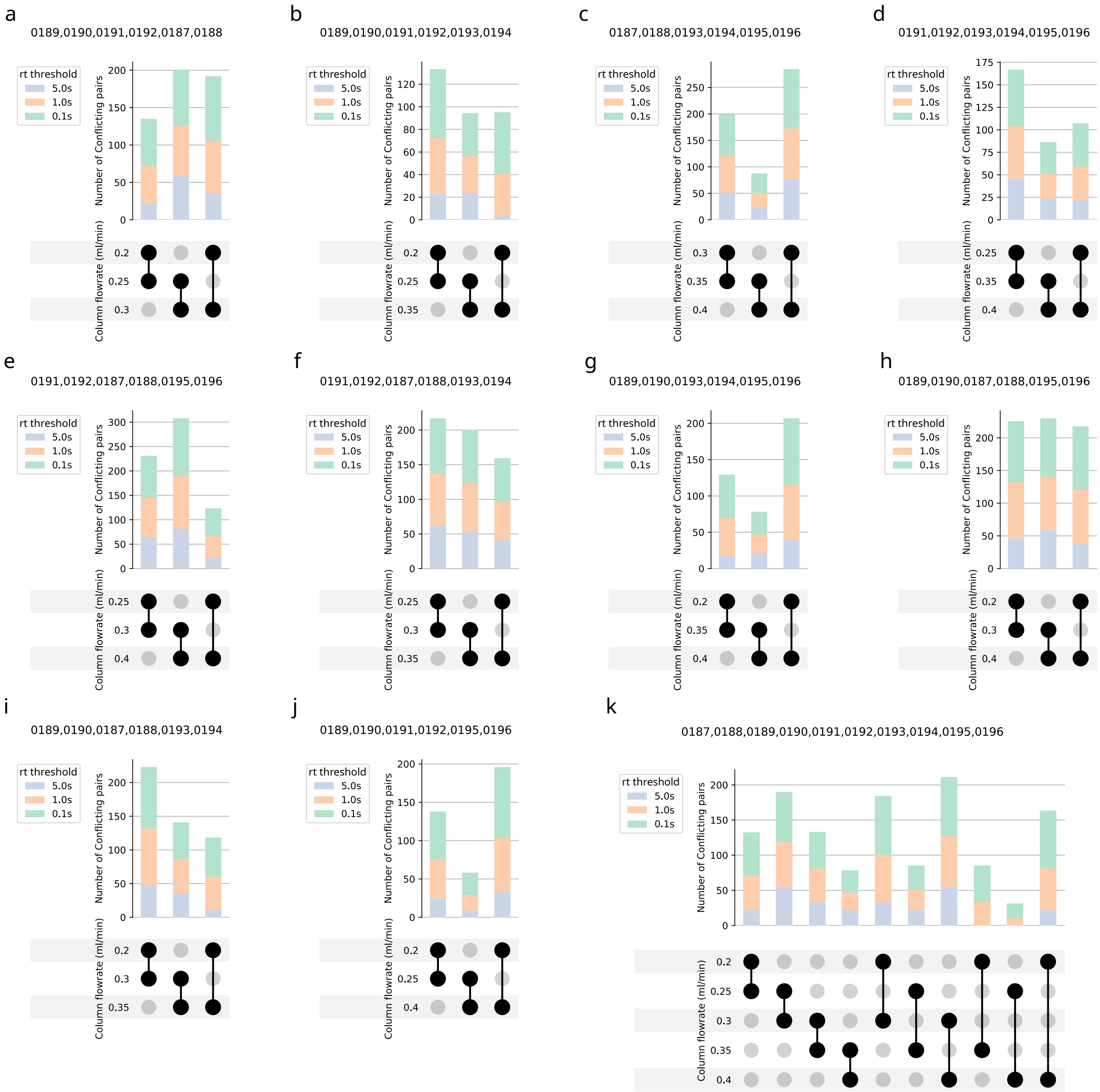


**Supplementary Figure 9. Influence of column flow rate on conflicting pairs.** Analogous to Supplementary Fig. 8, clusters of datasets where only the flow rate differs in the chromatographic setup are examined, the number of conflicting pairs in relation to the flow rate is shown. Note that RepoRT (as of August 2024) contains only one such cluster of datasets (k), although with five different settings for the flow rate. For the sake of better comparability to Supplementary Fig. 8, all combinations of three settings of flow rates are shown (a–j).

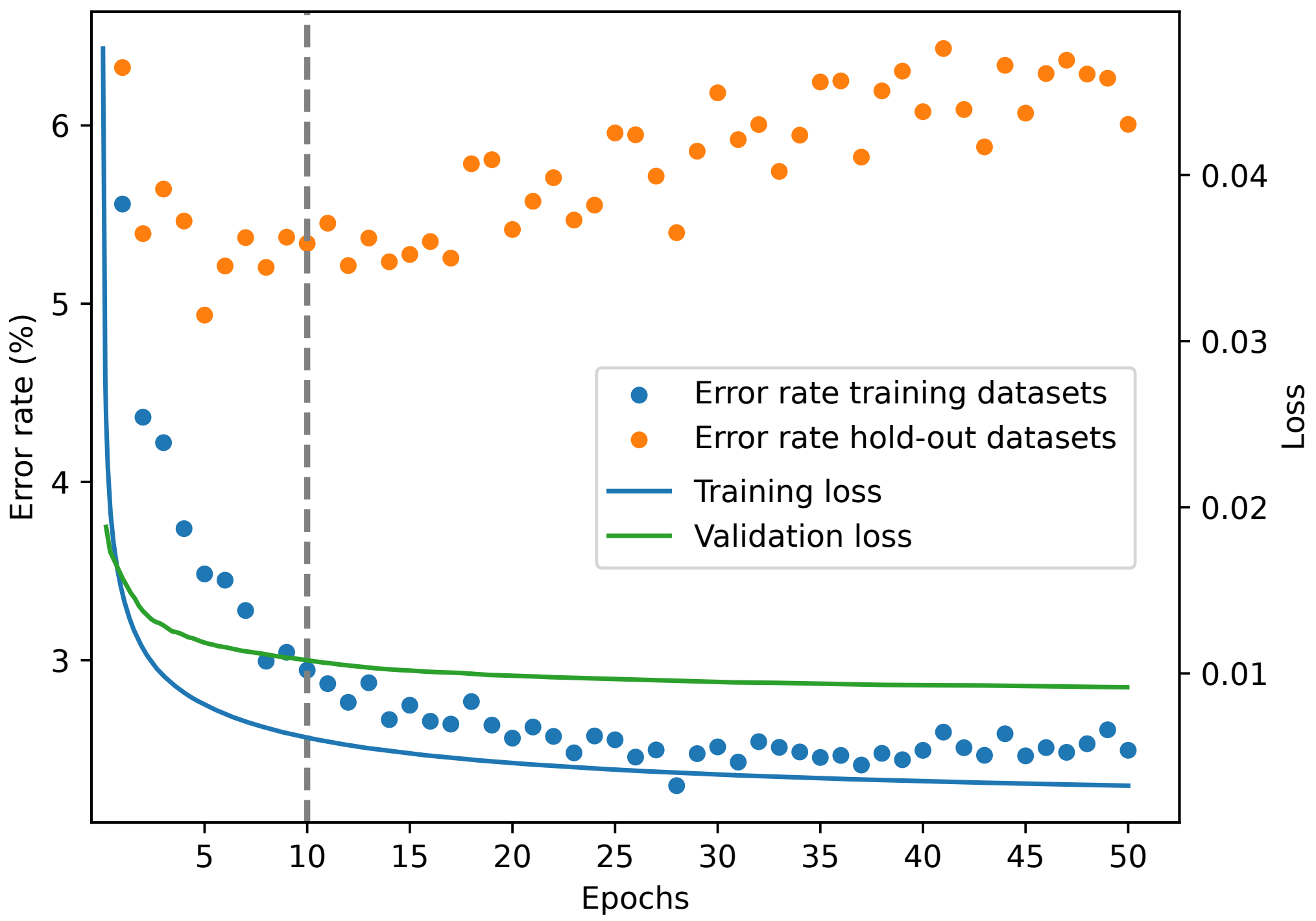


**Supplementary Figure 10. Training epochs for the ROI prediction model.** Shown are losses and error rates for an exemplary training process of our ROI prediction model, here trained for 50 epochs. Overfitting on the training data is only observable on hold-out datasets, resulting there in an increased error rate. Both the training loss and the validation loss keep decreasing, with the validation set here constituting compound pairs not part of the training data, but part of the same datasets. We decided against excluding whole datasets only for the purpose of establishing the optimal number of training epochs, and instead use a somewhat arbitrary fixed maximum number of ten epochs for all evaluations. A lower value is used when the error rate for training datasets decreases. We found this to work well in practice to counteract overfitting.

**Supplementary Table 1. List of RepoRT datasets used for retention order statistics and model evaluation.** All datasets from RepoRT are listed, detailing in which evaluation scenario each dataset is used. Information on which datasets are missing important metadata (HSM and Tanaka parameters, pH, void volume estimate, column temperature, flow rate) are also provided. Datasets removed from evaluation following manual curation are specified. This table is provided as a separate file.

**Supplementary Table 2. Comparison of retention time prediction performance for the uniform split.** Analogous to Table 1 we show mean absolute errors and standard deviations (over cross-validation folds) for the prediction of normalized retention times (in percentage points). Here we use cross-validation with a uniform split of the target dataset. The ROI prediction model is as always trained without the target datasets ("our 2-step method").

| **Method** | Pretrain | FEM long | IPB Halle | UniToyama Atlantis | Eawag XBridgeC18 | LIFE old | LIFE new |
|---|---|---|---|---|---|---|---|
| DeepGCN-RT | SMRT | 4.44 ± 1.18 | 3.14 ± 0.64 | **3.87** ± 1.96 | **3.07** ± 0.63 | 4.64 ± 1.56 | 7.01 ± 1.94 |
| RT-Transformer | SMRT | 8.35 ± 1.04 | 5.26 ± 1.39 | 7.45 ± 2.21 | 4.56 ± 0.68 | 4.12 ± 1.20 | 6.48 ± 1.93 |
| DeepGCN-RT | single RepoRT | 4.01 ± 1.00 | 2.94 ± 1.06 | 5.39 ± 1.97 | 3.10 ± 0.45 | 3.71 ± 0.71 | **4.61** ± 1.76 |
| RT-Transformer | single RepoRT | 10.86 ± 1.47 | 4.70 ± 0.77 | 6.34 ± 2.28 | 5.85 ± 0.87 | 5.92 ± 1.09 | 8.53 ± 2.16 |
| DeepGCN-RT | repeated RepoRT | **3.73** ± 1.07 | **1.75** ± 0.44 | 4.22 ± 1.66 | **3.07** ± 0.32 | 3.94 ± 1.22 | 5.27 ± 1.62 |
| RT-Transformer | repeated RepoRT | 7.76 ± 1.32 | 4.04 ± 0.75 | 7.51 ± 2.62 | 6.81 ± 0.94 | 5.19 ± 1.31 | 7.71 ± 1.86 |
| our 2-step method | | 4.78 ± 1.06 | 1.81 ± 0.50 | 5.84 ± 2.61 | 4.57 ± 0.96 | **3.10** ± 0.64 | 5.50 ± 0.80 |

**Supplementary Table 3. Extended comparison of retention time prediction performance.** We report mean absolute error (MAE), median absolute error (medAE) and coefficient of determination ($R^2$) for both the uniform split and the realistic split. We report average statistics over the six datasets FEM long, IPB Halle, UniToyama Atlantis, Eawag XBridgeC18, LIFE old, and LIFE new. To avoid that gradient length dominates reported errors, we normalized retention times so that the gradient length corresponds to 100 %. Reporting the mean relative error is unnecessary, because we already normalized using gradient length. For the pretrain/fine-tune methods, we report the dataset used to fine-tune the model; "no fine-tune" corresponds to the model being applied without fine-tuning with single pretraining on RepoRT. A negative coefficient of determination ('< 0') indicates that simply reporting the mean of the observed retention times, would outperform the actual predictions. See Supplementary Table 4 for the different ablation studies of our method ("w/o similar datasets", "w/o target compounds", "maximum challenge", "15 anchors'").

| | **Method** | Pretrain | MAE | medAE | $R^2$ |
|---|---|---|---|---|---|
| | DeepGCN-RT | R. single | 21.48 | 16.52 | < 0 |
| | RT-Transformer | no fine-tune | 21.29 | 15.82 | < 0 |
| uniform split | DeepGCN-RT | SMRT | 4.36 | 2.92 | 0.865 |
| uniform split | RT-Transformer | SMRT | 6.04 | 4.11 | 0.725 |
| uniform split | DeepGCN-RT | RepoRT single | 3.96 | 2.63 | 0.864 |
| uniform split | RT-Transformer | RepoRT single | 7.03 | 5.12 | 0.680 |
| uniform split | DeepGCN-RT | RepoRT repeated | **3.66** | **2.60** | **0.894** |
| uniform split | RT-Transformer | RepoRT repeated | 6.50 | 4.50 | 0.697 |
| uniform split | our 2-step method | | 4.27 | 2.92 | 0.830 |
| uniform split | w/o similar datasets | | 4.65 | 3.39 | 0.817 |
| uniform split | w/o target compounds | | 4.84 | 3.35 | 0.812 |
| uniform split | maximum challenge | | 5.01 | 3.66 | 0.799 |
| uniform split | 15 anchors | | 4.86 | 3.49 | 0.797 |
| realistic split | DeepGCN-RT | SMRT | 16.96 | 15.85 | < 0 |
| realistic split | RT-Transformer | SMRT | 8.42 | 6.16 | 0.590 |
| realistic split | DeepGCN-RT | RepoRT single | 7.73 | 5.94 | 0.638 |
| realistic split | RT-Transformer | RepoRT single | 8.32 | 6.76 | 0.623 |
| realistic split | DeepGCN-RT | RepoRT repeated | 7.42 | 5.98 | 0.697 |
| realistic split | RT-Transformer | RepoRT repeated | 8.74 | 7.01 | 0.570 |
| realistic split | our 2-step method | | **4.51** | **3.27** | **0.821** |
| realistic split | w/o similar datasets | | 5.32 | 4.11 | 0.795 |
| realistic split | w/o target compounds | | 5.41 | 4.07 | 0.792 |
| realistic split | maximum challenge | | 5.70 | 4.60 | 0.776 |
| realistic split | 15 anchors | | 4.86 | 3.49 | 0.797 |

**Supplementary Table 4. Ablation studies for retention time prediction performance.** Mean absolute errors and standard deviations for the prediction (over cross-validation folds) of normalized retention times (in percentage points) are shown for the realistic split. Results of DeepGCN-RT and RT-Transformer shown for comparison (repeated pretrain on RepoRT). Training of the ROI prediction model is performed without the target datasets ("our 2-step method"). Next, we removed certain parts of the training data when training the ROI model: First, we removed all datasets sharing the same combination of column and pH as the respective target datasets ("w/o similar datasets"). Second, we removed all compounds found in the target dataset from the training data ("w/o target compounds"). Consequently, if the model is evaluated on tryptophan, then it has not seen any data from tryptophan during training. For the "maximum challenge", both compounds and similar datasets are excluded. Anchors were established by ten-fold cross-validation. The "15 anchors" evaluation is different: Here, we again use the original "our 2-step" model for predicting ROI, but use only 15 randomly selected anchors to establish the mapping.

| **Method** | FEM long | IPB Halle | UniToyama Atlantis | Eawag XBridgeC18 | LIFE old | LIFE new |
|---|---|---|---|---|---|---|
| DeepGCN-RT | $8.26 \pm 2.57$ | $6.87 \pm 2.81$ | $5.41 \pm 1.99$ | $4.87 \pm 1.18$ | $9.87 \pm 3.37$ | $9.21 \pm 2.34$ |
| RT-Transformer | $13.96 \pm 4.62$ | $6.09 \pm 3.77$ | $8.43 \pm 3.63$ | $7.26 \pm 1.42$ | $7.34 \pm 2.31$ | $9.34 \pm 2.41$ |
| our 2-step method | $5.59 \pm 1.03$ | $1.78 \pm 0.45$ | $6.28 \pm 2.73$ | $4.68 \pm 4.79$ | $3.12 \pm 0.77$ | $5.59 \pm 1.36$ |
| w/o similar datasets | $5.87 \pm 0.85$ | $2.23 \pm 1.42$ | $5.78 \pm 2.39$ | $5.03 \pm 4.89$ | $5.94 \pm 1.45$ | $7.04 \pm 1.90$ |
| w/o target compounds | $5.91 \pm 0.70$ | $3.34 \pm 1.48$ | $5.67 \pm 2.88$ | $5.15 \pm 4.85$ | $5.82 \pm 2.21$ | $6.60 \pm 1.46$ |
| maximum challenge | $6.35 \pm 0.85$ | $3.65 \pm 0.83$ | $6.12 \pm 3.42$ | $5.57 \pm 4.89$ | $4.86 \pm 2.68$ | $7.63 \pm 1.86$ |
| 15 anchors | $5.39 \pm 0.53$ | $2.25 \pm 0.51$ | $6.49 \pm 0.63$ | $4.87 \pm 0.16$ | $3.63 \pm 0.70$ | $6.56 \pm 1.37$ |

**Supplementary Table 5. Non-normalized retention time prediction errors.** Mean absolute errors and standard deviations for the prediction (over cross-validation folds) of non-normalized retention times in minutes are shown for both uniform and realistic split. For a description of the ablation studies performed, see Supplementary Table 4. Results of DeepGCN-RT and RT-Transformer (repeated pretrain on RepoRT) are shown for comparison.

| | **Method** | FEM long | IPB Halle | UniToyama Atlantis | Eawag XBridgeC18 | LIFE old | LIFE new |
|---|---|---|---|---|---|---|---|
| uniform split | DeepGCN-RT | $2.24 \pm 0.64$ | $0.35 \pm 0.09$ | $1.69 \pm 0.66$ | $0.92 \pm 0.10$ | $0.24 \pm 0.07$ | $0.37 \pm 0.11$ |
| | RT-Transformer | $4.66 \pm 0.79$ | $0.81 \pm 0.15$ | $3.00 \pm 1.05$ | $2.04 \pm 0.28$ | $0.31 \pm 0.08$ | $0.54 \pm 0.13$ |
| | our 2-step method | $2.87 \pm 0.63$ | $0.36 \pm 0.10$ | $2.34 \pm 1.04$ | $1.37 \pm 0.29$ | $0.19 \pm 0.04$ | $0.39 \pm 0.06$ |
| | w/o similar datasets | $2.94 \pm 0.67$ | $0.42 \pm 0.08$ | $2.26 \pm 0.82$ | $1.45 \pm 0.30$ | $0.22 \pm 0.04$ | $0.48 \pm 0.08$ |
| | w/o target compounds | $3.13 \pm 0.63$ | $0.52 \pm 0.13$ | $2.11 \pm 0.86$ | $1.53 \pm 0.17$ | $0.29 \pm 0.05$ | $0.42 \pm 0.10$ |
| | maximum challenge | $3.25 \pm 0.74$ | $0.51 \pm 0.14$ | $2.47 \pm 0.69$ | $1.62 \pm 0.38$ | $0.23 \pm 0.05$ | $0.46 \pm 0.10$ |
| | 15 anchors | $3.23 \pm 0.32$ | $0.45 \pm 0.10$ | $2.60 \pm 0.25$ | $1.46 \pm 0.05$ | $0.22 \pm 0.04$ | $0.46 \pm 0.10$ |
| realistic split | DeepGCN-RT | $4.96 \pm 1.54$ | $1.37 \pm 0.56$ | $2.16 \pm 0.79$ | $1.46 \pm 0.36$ | $0.59 \pm 0.20$ | $0.64 \pm 0.16$ |
| | RT-Transformer | $8.38 \pm 2.77$ | $1.22 \pm 0.75$ | $3.37 \pm 1.45$ | $2.18 \pm 0.43$ | $0.44 \pm 0.14$ | $0.65 \pm 0.17$ |
| | our 2-step method | $3.35 \pm 0.62$ | $0.36 \pm 0.09$ | $2.51 \pm 1.09$ | $1.40 \pm 1.44$ | $0.19 \pm 0.05$ | $0.39 \pm 0.10$ |
| | w/o similar datasets | $3.52 \pm 0.51$ | $0.45 \pm 0.28$ | $2.31 \pm 0.96$ | $1.51 \pm 1.47$ | $0.36 \pm 0.09$ | $0.49 \pm 0.13$ |
| | w/o target compounds | $3.55 \pm 0.42$ | $0.67 \pm 0.30$ | $2.27 \pm 1.15$ | $1.55 \pm 1.46$ | $0.35 \pm 0.13$ | $0.46 \pm 0.10$ |
| | maximum challenge | $3.81 \pm 0.51$ | $0.73 \pm 0.17$ | $2.45 \pm 1.37$ | $1.67 \pm 1.47$ | $0.29 \pm 0.16$ | $0.53 \pm 0.13$ |
| | 15 anchors | $3.23 \pm 0.32$ | $0.45 \pm 0.10$ | $2.60 \pm 0.25$ | $1.46 \pm 0.05$ | $0.22 \pm 0.04$ | $0.46 \pm 0.10$ |

**Supplementary Table 6. Spectral library search combined with 2-step leads to nine "almost level 1" identifications.** Spectral library search was performed for MS/MS features in a LC-MS/MS dataset of fecal samples. After establishing a mapping from ROI to retention time from identified N-Alkylpyridinium 3-sulfonate measurements, we predicted retention time for detected spectral library matches (cosine similarity greater than 0.7, at least 4 shared peaks). For nine features, the match could be confirmed by matching predicted retention time (difference below 0.40, corresponding to the mean absolute error from cross-validation on the identified N-Alkylpyridinium 3-sulfonate measurements). The match to cholic acid was later manually discarded, as the associated adduct (ammonium) is unreasonable. ID refers to the internal feature ID (MetaboLights study accession MTBLS8356). PubChem compound IDs (PCID) are specified for each compound; IDs for reference spectra come from MassBank ("MSBNK-"), GNPS ("CCMSLIB"), or MSnLib ("BRUNGS_MCEBIO_"). 'SP', shared peaks.

| ID | **Compound name** | PCID | Cosine | SP | Reference ID | RT measured | RT predicted |
|---|---|---|---|---|---|---|---|
| 368 | 1,7-Dimethyluric acid | 91611 | 0.91 | 12 | MSBNK-Eawag-EQ01078704 | 1.98 | 2.29 |
| 500 | 1,5-isoquinolinediol | 1340 | 0.85 | 13 | BRUNGS_MCEBIO_04142 | 3.00 | 3.28 |
| 898 | Tributylamine | 7622 | 0.85 | 4 | MSBNK-Eawag-EQ315003 | 4.43 | 4.63 |
| 1008 | Pentapropylene glycol | 9836244 | 0.88 | 4 | CCMSLIB00003136844 | 4.62 | 4.55 |
| 1337 | Cholic acid | 221493 | 0.87 | 12 | CCMSLIB00006686258 | 6.14 | 6.09 |
| 1441 | Asiatic acid | 296191 | 0.72 | 27 | CCMSLIB00010113911 | 6.42 | 6.65 |
| 1456 | Tris(2-chloroisopropyl) phosphate | 26176 | 0.78 | 4 | MSBNK-Antwerp_Univ-AN117113 | 6.52 | 6.21 |
| 1786 | Dibutyl phthalate | 3026 | 0.96 | 6 | MSBNK-Eawag-EQ367004 | 7.41 | 7.39 |
| 1947 | Betulonic acid | 122844 | 0.74 | 39 | CCMSLIB00011427899 | 7.84 | 7.61 |

**Supplementary Table 7. Conflicting pairs statistics.** Number of compound pairs, informatory compound pairs, and conflicting pairs for different thresholds for minimum retention time difference. We consider only compound pairs with retention time difference above the threshold and with at most one of the compounds eluting in the void volume. Informatory compound pairs are compound pairs that change order ("conflict") for at least one dataset in RepoRT.

| **Threshold** | Compound pairs | Informatory compound pairs | Conflicting compound pairs |
|---|---|---|---|
| 10 $s$ | $86,554,044$ | $7,271,643$ | $1,730,993$ |
| 30 $s$ | $77,312,839$ | $2,582,316$ | $523,368$ |
| 60 $s$ | $65,528,382$ | $1,134,238$ | $204,477$ |

**Supplementary Table 8. Hyperparameters for the ROI prediction model explored in grid search.** We select the best configuration of hyperparameters from a grid search on a hold-out portion of 32 RepoRT datasets in five-fold cross-validation. Settings for the best-performing configuration are marked in bold. The values for hidden layer sizes refer to the embedding layers ("FFN 1") and dimensionality reduction layers ("FFN 2") with up to two layers each. Different margin values for the margin ranking loss were not explored in grid search, but evaluated only on the best configuration of all other hyperparameters.

| D-MPNN embedding size | message-passing steps | sizes FFN 1 | sizes FFN 2 | margin | error rate |
|---|---|---|---|---|---|
| 256 | 2 | 16 | 16 | 0.1 | 7.34% |
| | | 256 | 256 | | 7.95% |
| | | 256, 256 | 256, 64 | | 8.11% |
| | 3 | 16 | 16 | | 7.12% |
| | | 256 | 256 | | 7.16% |
| | | 256, 256 | 256, 64 | | 7.62% |
| | 4 | 16 | 16 | | 7.28% |
| | | 256 | 256 | | 7.50% |
| | | 256, 256 | 256, 64 | | 7.51% |
| **512** | 2 | 16 | 16 | | 7.20% |
| | | 256 | 256 | | 7.32% |
| | | 256, 256 | 256, 64 | | 7.97% |
| | **3** | 16 | 16 | | 6.91% |
| | | 256 | 256 | | 7.19% |
| | | **256, 256** | **256, 64** | | 6.88% |
| | 4 | 16 | 16 | | 7.06% |
| | | 256 | 256 | | 6.93% |
| | | 256, 256 | 256, 64 | | 6.89% |
| **512** | **3** | **256, 256** | **256, 64** | 0.001 | 7.06% |
| | | | | 0.01 | 7.46% |
| | | | | **0.1** | 6.88% |
| | | | | 0.2 | 7.44% |
