# Supplementary material for: Times are changing but order matters: transferable prediction of small-molecule liquid chromatography retention times
Source: Nat Methods. 2026 Oct 1;23(11):2219–29. doi: 10.1038/s41592-026-03243-2 (PMC13736644; doi:10.1038/s41592-026-03243-2)
Supplement: Supplementary file 3 [file 41592_2026_3243_MOESM3_ESM.pdf]

nature portfolio

Peer Review File

Times are changing but order matters: Transferable prediction of small molecule liquid chromatography retention times

Corresponding Author: Professor Sebastian Böcker

This file contains all editorial decision letters in order by version, followed by all author rebuttals in order by version.

A version of this paper was originally rejected for publication by Nature Methods, however that decision was reconsidered after appeal by the authors.

Version 0:

Decision Letter:

2nd Apr 2025

Dear Sebastian,

Your Article entitled "Times are changing but order matters: Transferable prediction of small molecule liquid chromatography retention times" has now been seen by 2 reviewers, whose comments are attached. In the light of their advice we have decided that we cannot offer to publish your manuscript in Nature Methods.

You will see that, while they find your work of some potential interest, the reviewers raise several serious concerns about the advance your methodological approach represents over available methods and about its broad applicability at this stage. They also question the novelty and evaluation of the approach as presented in the paper. We think that these criticisms are sufficiently important as to prevent publication of your work in Nature Methods.

Although we cannot publish your paper, it may be appropriate for another journal in the Nature Portfolio. If you wish to explore the journals and transfer your manuscript please use our manuscript transfer portal. You will not have to re-supply manuscript metadata and files, unless you wish to make modifications. For more information, please see our manuscript transfer FAQ page.

I am sorry that we cannot be more positive on this occasion but hope that you find the reviewers' comments helpful when preparing your paper for submission elsewhere.

Sincerely,
Arunima

Arunima Singh, Ph.D.
Senior Editor
Nature Methods

Reviewer Comments:

Reviewer #1 (Remarks to the Author):

The authors present a method to predict retention time of molecules on different chromatography setups. They present a 2-stage solution where a GNN model is trained to predict a retention order index and then a model to predict the retention time using the predicted retention order index.

Detailed description of how prior works relate to the contributions of current works needs to be clearly stated in the introduction.

The novelty needs to be highlighted to better place the work in context. The results also need to be put in context of other works.

Include in methods an explanation why a non-zero margin in the Margin Ranking Loss makes sense

Run a grid search for hyperparameter search (currently manually selected)

Include in methods an explanation for why you used a sigmoid in the final layer, why does ROI need to be a value between 0-1?

Please provide a better description of the siamese network and how it is used in this work so readers can understand this model

Describe in methods how Tanaka and HSM vectors are obtained

Describe in the paper what data is contained within RepoRT

“We also evaluated numerous modifications of model architecture and features, but doing so did not result in substantial improvements
of prediction performance (data not shown)” – please explain in more detail

Make sure DeepGCN-RT,RT-Transformer and your own models are trained on the same data

Explain in the paper why retention order prediction is a meaningful problem even if only ~7% of data are informative pairs

Explain in the paper why an accuracy below 60% on Setup-characteristic cases is good compared to 50% accuracy expected with random selection

Explain in the paper why people should use your model if the retention time predictions have lower accuracy than DeepGCN-RT

Include other retention time prediction evaluation metrics prior works (DeepGCN-RT, RT-Transformer) evaluated on including R^2, MedAE, etc.

In the paper, add a description for how DeepGCN-RT and RT-Transformer work

In the paper, add a description for how prior models were fine tuned for the target dataset, what data was used for fine tuning vs testing

Minor Comments
Larger fonts across all figures
The number of compound pairs under different thresholds and the number of conflicting pairs should be placed in a table
STable 3 should be moved to the main text
Explicitly state where the weight function f(x) is used

Reviewer #2 (Remarks to the Author):

The primary contribution of this paper is to develop a Graphical Neural Networking with message passing that is able generalise retention time prediction across samples analysed under different conditions. The method appears relatively sound and there is some analysis to show why it might be necessary. I am not completely convinced by the novelty improvements offered by the work. No comparison is done for the actual situation where this method would be used, so its unclear how much it is better than existing non-transferable methods, although presumably these results would be positive. I am also not sure its contribution compared to say ref18, or other competitor methods mention, as the GNN+MP is not novel in itself. My conclusion is that the method is probably nice, but I am not convinced that the contribution is enough for this level of paper.

Grammer and language wise, the paper is fine. However, beyond that I find it poorly written, lacking consistent motivation for the reader throughout the paper, and lacking adequate comparison to existing methods. On second read it becomes clearer, but it would need a substantial rewrite in my opinion even if the scientific contribution is judged to be sufficient. It took be to read 10 pages and 2 pages of comments and questions before I fully understood what the method was and why it was useful. Tables and figures generally need improving as well.

Despite some of these comments, I could see a possibility of accepting the paper, but it would need a substantial rewrite, more analysis with motivated explanation, and more links to the existing literature

Below are more specific comments.

Comments by Section

Introduction – the introduction gives a nice introduction to some of the background (and is well written), but strangely (at least in my eyes) does not mention the work in this paper, or more crucially give the reader any idea how this work fits with the existing research

Figure 1a – there is not enough explanation here to do more than guess what is going on. Would suggest making it bigger with a more detailed caption (e.g. move side titles to top titles, double width and height of images, add an explanation for each image). Maybe need to make arrows thicker as well. Images are great if bigger and with explanations

Results
- 'see below' – completely unclear what this means
- 'dataset boundaries' – completely unclear what this means
- I don't see how 'chromographic conditions to be "nominally identical"' leads to 'Accordingly… is futile' – you define something, then that leads to something being futile, doesn't really make sense.
- 'highly restricted compound' – hard to follow this, but probably relates to previous comment
- 'such deviations' – what deviations?
- Is this second paragraph meant to be an introduction to the results section? Why is it given a summary of results we have seen yet of studies we don't know what they? How is the reader meant to have any idea what is going on? It needs to be written in a way that explains what you want to do. Maybe it would make sense if there was a summary saying what the paper was about in the introduction but currently it makes no sense.
- Be more precise than 'machine learning model', this would be equivalently vague to describing all the data as being from a 'chemistry experiment'

Conflicting pairs as a measure of disagreement.
- vs. to versus
- This section is lacking some motivation for the reader, probably as its missing an introduction type chunk of text at the start to give context
- The final conclusions is essentially a list of numbers. Presumably this motivates the results of the remaining sections, but again the reader is not told that and has to guess. I am not convinced a list of numbers with little motivation is Nature Methods worthy, sorry!

Influence of chromatographic conditions on retention order
- Same comment as before, needs clear introduction and motivation, and needs linking to your actual methods
- So far both results sections are essentially summary statistics and results, not particularly inspiring
- Figure 2 – quality leaves a bit to be desired. Figures are already very small, then there is small writing and thin lines. Its acceptable at 200% zoom on my computer, but only just.

Predicting retention order indices
- Please explain how the graph structure is set up for these predictions, and why intuitively that might be helpful. Is it the graph structure in Fig 3a, e.g. the metabolite, if so then intuitively how is this represented? – there is more information in the methods, but a little more is needed here
- 'To predict the retention order, the two ROIs are subtracted' – why is this necessary? Is one number being bigger/smaller than another not sufficient for this?
- Is the key advance here compared to existing methods that you combine the experimental conditions with the graph representation? I think this section could do with explaining more why you are different to existing methods. The introduction to the paper, did a nice overview of methods which appear to do the same task as what you describe. You compare to one, presumably much simpler, method from 7 years ago, surely there are simpler NN / GNN approaches that would be more modern, speedier, and comparable performance wise.
- What is the relationship of your work in this section to ref18? They must be doing a similar thing given the title?

Mapping retention order indices to retention times
- You mention two competitor methods (RTTransformer and DeepGCN-RT). Based on the name alone, these must be relatively similar concept at the basic ML level. Please give some intuition around what the similarities and differences are to your method. What is the intuitive reason why your method performs better and are these methods designed for the same task or for a fixed experimental setup?
- Table 1 – I struggled to work out which your methods even were. I have concluded its all but the top two? Then it's a load of different scenarios? My understanding is then that they have partially seen target data and you have not and that is why your methods are worse? If so, then essentially the results aren't comparable between the first two rows and the rest? Is there some form of fair comparison that you can do? What would happen if you naively used the two existing methods on data they had not seen before, or is the point that this would just not work at all? There must be some methods that you can compare to in a fair way, where both methods have not seen the target data. Apologies if I have misunderstood here, I have struggled with the writing style and organisation of your paper.
- Having read further I now understand that matching performance would be a very good thing. This needs to be made clearer to the reader throughout, I think you are lacking the motivation in the writing, which I think is actually very sound, just not explained. An additional comparison that shows the other methods are not transferable between datasets would be beneficial, even if it shows that your results are much better
- least squares optimization – least squares is the solution method and therefore does not need optimising (instead calculating). You could optimise the loss of an MSE which would be essentially the same.

Training retention order index prediction models

- is 10 epochs enough for any form of convergence, seems unlikely. Have you explored what the effects of changing this is?

Reviewer #2 (Remarks on code availability):

Code looks sensible and well organised

** For Nature Portfolio general information and news for authors, see http://npg.nature.com/authors.

Version 1:

Decision Letter:

23rd Jun 2025

Dear Sebastian,

Thank you for your letter asking us to reconsider our decision on your Article, "Times are changing but order matters: Transferable prediction of small molecule liquid chromatography retention times". After careful consideration we have decided that we are willing to consider a revised version of your manuscript. The revision plan sounds reasonable and I'm happy you are willing to include the analysis to resolve stereoisomers as a practical application of your work.

When revising your paper:

* include a point-by-point response to our referees and to any editorial suggestions

* please underline/highlight any additions to the text or areas with other significant changes to facilitate review of the revised manuscript

* address the points listed described below to conform to our open science requirements

* ensure it complies with our general format requirements as set out in our guide to authors at www.nature.com/naturemethods

* resubmit all the necessary files electronically by using the link below to access your home page

Link Redacted

Note: This URL links to your confidential home page and associated information about manuscripts you may have submitted, or that you are reviewing for us. If you wish to forward this email to co-authors, please delete the link to your homepage.

We hope to receive your revised paper within 6 weeks. If you cannot send it within this time, please let us know. In this event, we will still be happy to reconsider your paper at a later date so long as nothing similar has been accepted for publication at Nature Methods or published elsewhere.

OPEN SCIENCE REQUIREMENTS

REPORTING SUMMARY
When revising your manuscript, please submit a reporting summary.

Reporting summary: https://www.nature.com/documents/nr-reporting-summary.zip

If your paper includes custom software, we also ask you to complete a supplemental reporting summary.

Software supplement: https://www.nature.com/documents/nr-software-policy.pdf

Please submit these with your revised manuscript. They will be available to reviewers to aid in their evaluation if the paper is re-reviewed. If you have any questions about the checklist, please see http://www.nature.com/authors/policies/availability.html or contact me.

Please note that these forms are dynamic ‘smart pdfs’ and must therefore be downloaded and completed in Adobe Reader. We will then flatten them for ease of use by the reviewers. If you would like to reference the guidance text as you complete the template, please access these flattened versions at http://www.nature.com/authors/policies/availability.html.

For any revision that includes light microscopy data, we ask our authors to please include a completed light microscopy reporting table [https://www.nature.com/documents/Light_microscopy_reporting_table.xlsx] to ensure the methods are described thoroughly. The table will be available to reviewers and ultimately published should the manuscript be accepted at the journal.

DATA AVAILABILITY
Please include a “Data availability” subsection in the Online Methods. This section should inform readers about the availability of the data used to support the conclusions of your study, including accession codes to public repositories, references to source data that may be published alongside the paper, unique identifiers such as URLs to data repository entries, or data set DOIs, and any other statement about data availability. At a minimum, you should include the following statement: “The data that support the findings of this study are available from the corresponding author upon request”, describing which data is available upon request and mentioning any restrictions on availability. If DOIs are provided, please include these in the Reference list (authors, title, publisher (repository name), identifier, year). For more guidance on how to write this section please see: http://www.nature.com/authors/policies/data/data-availability-statements-data-citations.pdf

CODE AVAILABILITY
Please include a “Code Availability” subsection in the Online Methods which details how your custom code is made available. Only in rare cases (where code is not central to the main conclusions of the paper) is the statement “available upon request” allowed (and reasons should be specified).

We request that you deposit code in a DOI-minting repository such as Zenodo, Gigantum or Code Ocean and cite the DOI in the Reference list. We also request that you use code versioning and provide a license.

For more information on our code sharing policy and requirements, please see: https://www.nature.com/nature-research/editorial-policies/reporting-standards#availability-of-computer-code

MATERIALS AVAILABILITY
As a condition of publication in Nature Methods, authors are required to make unique materials promptly available to others without undue qualifications.

Authors reporting new chemical compounds must provide chemical structure, synthesis and characterization details. Authors reporting mutant strains and cell lines are strongly encouraged to use established public repositories.

More details about our materials availability policy can be found at https://www.nature.com/nature-portfolio/editorial-policies/reporting-standards#availability-of-materials

SUPPLEMENTARY PROTOCOL
To help facilitate reproducibility and uptake of your method, we ask you to prepare a step-by-step Supplementary Protocol for the method described in this paper. We encourage authors to share their step-by-step experimental protocols on a protocol sharing platform of their choice and report the protocol DOI in the reference list. Nature Portfolio's protocols.io is a free-to-use and open resource for protocols; protocols deposited onto protocols.io are citable and can be linked from the published article. More details can found at protocols.io.

ORCID
Nature Methods is committed to improving transparency in authorship. As part of our efforts in this direction, we are now requesting that all authors identified as ‘corresponding author’ on published papers create and link their Open Researcher and Contributor Identifier (ORCID) with their account on the Manuscript Tracking System (MTS), prior to acceptance. This applies to primary research papers only. ORCID helps the scientific community achieve unambiguous attribution of all scholarly contributions. You can create and link your ORCID from the home page of the MTS by clicking on ‘Modify my Springer Nature account’. For more information please visit please visit www.springernature.com/orcid.

We look forward to hearing from you soon.

Sincerely,
Arunima

Arunima Singh, Ph.D.
Senior Editor
Nature Methods

Version 2:

Decision Letter:

26th Nov 2025

Dear Professor Böcker,

I am writing to you on behalf of your editor Dr. Arunima Singh, who is out of the office this week.

Thank you for your letter detailing how you would respond to the reviewer concerns regarding your Article, "Times are changing but order matters: Transferable prediction of small molecule liquid chromatography retention times". I understand that Arunima sent you a follow-up email, but had not heard back from you. She will be happy to continue the discussion of how to revise the manuscript with you when she is back in the office next week.

For now, I am sending you the decision letter with a link to resubmit when ready. Please do not hesitate to contact us if you have any questions or would like to discuss these revisions further.

When revising your paper:

* include a point-by-point response to the reviewers and to any editorial suggestions

* please underline/highlight any additions to the text or areas with other significant changes to facilitate review of the revised manuscript

* address the points listed described below to conform to our open science requirements

* ensure it complies with our general format requirements as set out in our guide to authors at www.nature.com/naturemethods

* resubmit all the necessary files by using the link below to access your home page

Link Redacted

Note: This URL links to your confidential account and associated information about manuscripts you may have submitted, or that you are reviewing for us. If you wish to forward this email to co-authors, please delete this link.

We hope to receive your revised paper within 2 months. If you are substantially delayed, please let us know. In this event, we will still be happy to reconsider your paper at a later date so long as nothing similar has been accepted for publication at Nature Methods or published elsewhere.

OPEN SCIENCE REQUIREMENTS

REPORTING SUMMARY
When revising your manuscript, please update your reporting summary.

Reporting summary: https://www.nature.com/documents/nr-reporting-summary.zip

If your paper includes custom software, we also ask you to complete a supplemental reporting summary.

Software supplement: https://www.nature.com/documents/nr-software-policy.pdf

Please submit these with your revised manuscript. They will be available to reviewers to aid in their evaluation if the paper is re-reviewed. If you have any questions about the checklist, please see http://www.nature.com/authors/policies/availability.html or contact me.

Please note that these forms are dynamic ‘smart pdfs’ and must therefore be downloaded and completed in Adobe Reader. We will then flatten them for ease of use by the reviewers. If you would like to reference the guidance text as you complete the template, please access these flattened versions at http://www.nature.com/authors/policies/availability.html.

For any revision that includes light microscopy data, we ask our authors to please include a completed light microscopy reporting table [https://www.nature.com/documents/Light_microscopy_reporting_table.xlsx] to ensure the methods are described thoroughly. The table will be available to reviewers and ultimately published should the manuscript be accepted at the journal.

EXTENDED DATA FIGURES

When re-submitting your manuscript, please ensure that any supplementary figures and tables that are crucial to the manuscript's conclusions are converted into Extended Data figures and tables to increase visibility of these data. Extended Data figures and tables are online-only (present in the online PDF and full-text HTML versions of the paper), peer-reviewed display items that provide essential background to the article but are not included in the main article due to space constraints. A maximum of ten Extended Data display items (figures and tables) is permitted.

DATA AVAILABILITY
Please include a "Data availability" subsection in the Online Methods. This section should inform readers about the availability of the data used to support the conclusions of your study, including accession codes to public repositories, references to source data that may be published alongside the paper, unique identifiers such as URLs to data repository entries, or data set DOIs, and any other statement about data availability. At a minimum, you should include the following statement: "The data that support the findings of this study are available from the corresponding author upon request", describing which data is available upon request and mentioning any restrictions on availability. If DOIs are provided, please include these in the Reference list (authors, title, publisher (repository name), identifier, year). For more guidance on how to write this section please see:
http://www.nature.com/authors/policies/data/data-availability-statements-data-citations.pdf

For papers containing bioimaging data, we strongly recommend depositing the data to Bioimage Archive (https://www.ebi.ac.uk/bioimage-archive/). Associated accession codes and hyperlinks should be provided in the "Data Availability" section.

CODE AVAILABILITY
Please include a "Code Availability" subsection in the Online Methods which details how your custom code is made available. Only in rare cases (where code is not central to the main conclusions of the paper) is the statement "available upon request" allowed (and reasons should be specified).

We request that you deposit code in a DOI-minting repository such as Zenodo, Gigantum or Code Ocean and cite the DOI in the Reference list. We also request that you use code versioning and provide a license.

For more information on our code sharing policy and requirements, please see:
https://www.nature.com/nature-research/editorial-policies/reporting-standards#availability-of-computer-code

MATERIALS AVAILABILITY
As a condition of publication in Nature Methods, authors are required to make unique materials promptly available to others without undue qualifications.

Authors reporting new chemical compounds must provide chemical structure, synthesis and characterization details. Authors reporting mutant strains and cell lines are strongly encouraged to use established public repositories.

More details about our materials availability policy can be found at https://www.nature.com/nature-portfolio/editorial-policies/reporting-standards#availability-of-materials

ORCID
Nature Methods is committed to improving transparency in authorship. As part of our efforts in this direction, we are now requesting that all authors identified as 'corresponding author' on published papers create and link their Open Researcher and Contributor Identifier (ORCID) with their account on the Manuscript Tracking System (MTS), prior to acceptance. This applies to primary research papers only. ORCID helps the scientific community achieve unambiguous attribution of all scholarly contributions. You can create and link your ORCID from the home page of the MTS by clicking on 'Modify my Springer Nature account'. For more information please visit please visit www.springernature.com/orcid.

Please do not hesitate to contact us if you have any questions or would like to discuss these revisions further. We look forward to seeing the revised manuscript and thank you for the opportunity to consider your work.

Best regards,
Allison

Allison Doerr, Ph.D.
Chief Editor
Nature Methods

Reviewers' Comments:

Reviewer #1 (Remarks to the Author):

overall great improvement in the clarity of the paper. Because this was a major rewrite and the authors chose not to highlight their changes, I reviewed it afresh. Here are the comments.

1. Biggest concern is the wordiness of the paper. Please try to re-write for conciseness. Some examples are listed below.
2. Justification of the importance of the retention problem is not clearly articulated. Please provide better explanation why this problem is important and how it helps with elucidation and how it offers a new paradigm different than the existing one. The # of hits in google is not sufficient justification that it is an important problem. Unfortunately, the application does not show until the supplementary. Please move parts or some into the main text to stress the importance of this technique
3. Please provide in the manuscript an intuitive explanation of "Retention order is known to be much better conserved than retention time2"
4. This sentence is not clear: "This differs from predicting retention indices mentioned above, where we first choose a particular index type, then try to predict it." Please re-write this sentence and others for clarity (not sure what is mentioned above, retention times?)
5. Please re-phrase: "and this is extremely unlikely to happen in the future:" – just seems unnecessarily pessimistic considering it is not the main focus of the paper.
6. You introduce a new term, "Transferable retention time prediction", but it is not defined anywhere
7. In the manuscript, add a description of the SMRT dataset, p. 9, and put in context for the reader – I just now know it is larger than all others.
8. There is a "challenging split" and a "realistic split". Please add a couple of sentences to justify the use of these labels – used in prior publications? I like the "maximum challenge" terminology!
9. Paragraph, "Finally, we performed ablation studies..". Please add a sentence or two summary to explain the results (instead of having to look into the supplementary).
10. Why not use grid search for Ranknet?
11. Fixing the number of training epochs: Why not stop on validation loss as is standard in practice. Even some models can overfit with 10 epochs.
12. There is a lot of repetition on what was removed: in the void volume, doublets, compound pair, etc.. Search for these key words and see if you can consolidate the description please to reduce redundancy.
13. The evaluation is sometimes reported as accuracy and sometimes as error rate. Is there a method or pattern? Please explain or use the same metric.
14. Not clear how the "Measuring MS/MS libraries" section is substantially different from "Deconvolution of chimeric spectra". Please re-write to clarify, and if related combine.
15. Supplementary Note 2 seems redundant, and its tone is inappropriate for publication. Please remove or re-write, or potentially add in a table format for emphasizing the point, if not covered in the paper?
16. Supplementary Figure 18 is confusing. You are assuming you are not doing library search first? Why does the reader need to see candidates 3, 4, and 9? Maybe re-write to clarify what step is happening first, and how the cosine similarities from the library are being used. There is no clear methodology on how to combine SIRIUS or other annotation techniques and retention time.
17. Please use newer references for de novo generation.
18. Supplementary Figure 9 panels are not provided. Not clear what the peaks are showing
19. Add a limitation that it is not clear how to incorporate retention time prediction with generation or annotation. Lots of research to be done!

Minor (no need to respond - just fix at discretion
1. what is "For setup-characteristic compound pairs" ? and which two models are you referring to? P. 8 , first full paragraph
- "been repeatedly been", p. 9
- explain LIFE old and FEM long, p. 9
- don't use method and model interchangeably, specially in the context of training, e.g., p10
2. -"hide the extend". P. 12
- "selectivity in LC" p. 13. What does it refer to?
3. -" We use the Euclidean distance between parameter HSM and Tanaka vectors to measure
4. column differences." Is this referring to the one-hot encoding?
5. "Second, we studied the influence" ? first was analyzed the fraction of conflicting pairs in RepoRT? And then Third and fourth are close to each other. Maybe rephrase to make it easy to follow
6. Can you clarify how these are datasets, yet 19 datasets were excluded, "For FEM long, IPB Halle, LIFE new, 19 datasets were excluded"?
7. "six pairs informatory"
8. P. 33, What does this mean, "pretrain/fine-tune methods solely for readability"?
9. decide for our --> decide on?

Reviewer #2 (Remarks to the Author):

Firstly, apologies for the slow re-review of the paper, it arrived just before a month of holiday and conferencing!

Thank you for the update to the paper. Generally, I found the paper to me much more readable with the only stylistic issues being those enforced by the journal! It now reads very well and I did not need to read the paper multiple times to understand what is going on and what the advantages are of the methods over existing similar sounds deep learning methods.

I think its now really clearly demonstrated what the advantages are off the methods. Namely, that by incorporating the chromatographic settings into the GNN, you are able to generalise to new setup. This is achieved by combining these chromatographic settings with reference annotations that can be made using any sample, rather than requiring a large number of experiments to gather reference data. The result is that this work is important for metabolite identifications on any new LC setup or after changes to an existing setup. Based on this, I think this method does offer a valuable contribution to the community and would be used by many people to help with their identifications. I would therefore be happy to see the work published in this journal.

One final comment for future consideration. In the future you might still want to consider more epochs if you can. There is a phenomenon in deep learning called double descent where you initially see a graph like the final one in your reviewer response and then eventually you see a second decent in the test error rate. I am not saying that is necessarily happening here, just something to consider looking at.

Reviewer #2 (Remarks on code availability):

Code looks reasonable and well maintained

Reviewer #3 (Remarks to the Author):

The authors introduce a method for transferable prediction of the elution retention time of small molecules in reversed-phase liquid chromatography. Their innovative approach involves dividing the process into two steps. The first step is to train a [structure+LC conditions] to retention order (index) predictor. This is a computationally intensive and data-demanding task, but it was “solved" by the authors using a large collection of reported experimental data. Conversion from ROIs to retention time is then performed in the second step, where a simple polynomial is fitted. This second task is much simpler, and the authors demonstrate that a limited number of anchor metabolites (identified through MS2-matching) is sufficient.

I noticed that the original reviewers raised reasonable concerns regarding the clarity of the text and presentation, the novelty of the method, its impact, and its broad applicability. As the method and the statistical analyses are robust, I will focus on these four points.

1) Text quality
I understand that the authors have already rewritten the manuscript entirely for the revised version, but it still remains inadequate. The text stays wordy, has a weak structure, often includes unnecessary details, introduces “new” problems and concepts too early, fails to motivate what’s coming next or to conclude sections, and switches between design and benchmarking in a confusing way. More effort is required to streamline the manuscript.

By admission of the authors, “The manuscript is too long.” Large portions of the statistical analysis and the final part on the influence of LC conditions on order can be shortened or moved to the supplement. The analysis of factors affecting order and time is, in fact, of little interest. The main conclusion is that “name, column, mobile phase, gradient, temperature, and flow rate” influence retention order. Isn’t this obvious?

The abstract needs revision. Rewrite the first four sentences to clearly state what was accomplished. Avoid phrases like “Our goal is…”, “Our method should…”. This section is a summary of results, not an introduction. Avoid vague statements such as “Unfortunately, retention times may change massively, even for nominally identical chromatographic conditions,” especially if you already demonstrate the opposite — or clarify what “nominally” means. The results section must be precise: the new method does NOT outperform DeepGCN-RT, except when focusing on compounds that are very structurally different. The last sentence is of limited relevance and should be replaced with a statement on broader impact and applications. The introduction could also be shortened by combining the last paragraph with the first. The paragraph on retention time indexes could instead be moved later in the results section, when describing the 2-step approach.

2) Novelty
The novelty of the new method is sufficiently clear in the revision. (i) The architecture with the split (structure + LC conditions → ROI, and ROI + anchors → RT prediction) is novel, (ii) they are the first to demonstrate transferable predictions.

3) Impact and wide applicability
By designing a transferable prediction system, the authors established a foundation that ensures broad applicability. Any scientist can predict elution times in their own reversed-phase LC setup.

However, I have several concerns about the impact. The main reason is that the authors have focused on demonstrating performance, but little has been done to illustrate the impact.

For example, the results (Figure 4) show that the new method is comparable to the state-of-the-art model trained on the training data (DeepGCN-RT), but the new method generalises better when tested on a subset of molecules that differ from the training set (' realistic split”). This is important from a theoretical perspective, but not a decisive argument. Are there good examples to

demonstrate that better transfer leads to improved annotation?

In my view, the impact of improved RT prediction should be assessed by its ability to disambiguate isomers in LC feature annotation, which could involve known compounds that are structurally similar to others in the sample. I assume this is what the analysis of the faecal sample dataset aimed to show (Figure S17), but this part is buried at the end of the methods, not connected or referenced elsewhere in the paper, and lacking figures in the main text or citations in the manuscript, abstract, or conclusion. I wondered whether this is a remnant from an earlier version of the manuscript. Perhaps it’s because the two cholate examples shown are not particularly impressive, as they could be distinguished by different LC methods, and probably also by Deepgcn-RT. In any case, analyzing the impact of the new method on isomer annotation is crucial to demonstrate the broader significance of the improved RT prediction.

Finally, there is also the limitation of the existing method to reversed-phase LC, which is adequate for moderately polar to nonpolar compounds, including drugs, but not suitable for polar metabolites that differ by the position or orientation of a polar group. This limitation is discussed in the conclusion, highlighting HILIC as the next major challenge. My impression is that, regardless of how sophisticated and elegant the new method already is, its impact remains limited until transferable predictions and impact are demonstrated for non–reversed-phase LC.

Minor points
• It would be helpful for the broader readership if Figure 1A presented the overall architecture, highlighting what step 1 and step 2 are, and where and what experimental data flow into (what is now divided across 1e–g). Details on the architecture or training of the ROI should be moved to 1B etc.
• Figure 3: What data are shown? Are these 3 studies from MetaboLights?
• Figure 4 and Table 2: What is the unit? Is it seconds or delta(normalized retention time)?
• Supp Figure 17: Check the molecular structure shown in panel g: if I remember correctly, the methyl group in the fatty acyl chain is in the opposite direction.

Version 3:

Decision Letter:

Our ref: NMETH-A59192C

31st Mar 2026

Dear Sebastian,

Thank you for submitting your revised manuscript "Times are changing but order matters: Transferable prediction of small molecule liquid chromatography retention times" (NMETH-A59192C). We find that the paper has improved in revision, and therefore we'll be happy in principle to publish it in Nature Methods, pending final requests to comply with our editorial and formatting guidelines.

We are now performing detailed checks on your paper and will send you a checklist detailing our editorial and formatting requirements within two weeks or so. Please do not upload the final materials and make any revisions until you receive this additional information from us.

TRANSPARENT PEER REVIEW
Nature Methods offers a transparent peer review option for new original research manuscripts submitted from 17th February 2021. We encourage increased transparency in peer review by publishing the reviewer comments, author rebuttal letters and editorial decision letters if the authors agree. Such peer review material is made available as a supplementary peer review file. **Please state in the cover letter ‘I wish to participate in transparent peer review’ if you want to opt in, or ‘I do not wish to participate in transparent peer review’ if you don’t.** Failure to state your preference will result in delays in accepting your manuscript for publication.

Please note: we allow redactions to authors’ rebuttal and reviewer comments in the interest of confidentiality. If you are concerned about the release of confidential data, please let us know specifically what information you would like to have removed. Please note that we cannot incorporate redactions for any other reasons. Reviewer names will be published in the peer review files if the reviewer signed the comments to authors, or if reviewers explicitly agree to release their name. For more information, please refer to our FAQ page.

ORCID
IMPORTANT: Non-corresponding authors do not have to link their ORCIDs but are encouraged to do so. Please note that it will not be possible to add/modify ORCIDs at proof. Thus, please let your co-authors know that if they wish to have their ORCID added to the paper they must follow the procedure described in the following link prior to acceptance:
https://www.springernature.com/gp/researchers/orcid/orcid-for-nature-research

Author names using non-Roman characters
Nature Portfolio journals can support presentation of author names using non-Roman characters in the HTML version of the article. If you wish to, please include author names in parentheses after the Roman-character spelling; <a

href="https://www.nature.com/articles/s44222-024-00258-2">see example online here. Currently supported scripts are: Arabic, Chinese, Cyrillic, Devanagari, Greek, Hebrew, Hangul, Japanese and Persian. You will be asked to verify the rendering is correct at proof stage.

Thank you again for your interest in Nature Methods. Please do not hesitate to contact me if you have any questions. We will be in touch again soon.

Sincerely,
Arunima

Arunima Singh, Ph.D.
Senior Editor
Nature Methods

Version 4:

Decision Letter:

25th Aug 2026

Dear Professor Böcker,

I am pleased to inform you that your Article, "Times are changing but order matters: Transferable prediction of small molecule liquid chromatography retention times", has now been accepted for publication in Nature Methods. The received and accepted dates will be December 20, 2024 and August 25, 2026. This note is intended to let you know what to expect from us over the next month or so, and to let you know where to address any further questions.

Over the next few weeks, your paper will be copyedited to ensure that it conforms to Nature Methods style. Once your paper is typeset, you will receive an email with a link to choose the appropriate publishing options for your paper and our Author Services team will be in touch regarding any additional information that may be required. It is extremely important that you let us know now whether you will be difficult to contact over the next month. If this is the case, we ask that you send us the contact information (email, phone and fax) of someone who will be able to check the proofs and deal with any last-minute problems.

After the grant of rights is completed, you will receive a link to your electronic proof via email with a request to make any corrections within 48 hours. If, when you receive your proof, you cannot meet this deadline, please inform us at rjsproduction@springernature.com immediately.

Authors may need to take specific actions to achieve compliance with funder and institutional open access mandates. If your research is supported by a funder that requires immediate open access (e.g. according to Plan S principles or the NIH public access policy) then you should select the gold OA route, and we will direct you to the compliant route where possible. Because authors warrant under our subscription licensing terms that they haven’t committed to licensing any version of their article under a licence inconsistent with the terms of our agreement – including the applicable embargo period – publication under the subscription model isn’t suitable for authors whose funders require no embargo.

If you have any questions about our publishing options, costs, Open Access requirements, or our legal forms, please contact ASJournals@springernature.com

If you have posted a preprint on any preprint server, please ensure that the preprint details are updated with a publication reference, including the DOI and a URL to the published version of the article on the journal website.

You may wish to make your media relations office aware of your accepted publication, in case they consider it appropriate to organize some internal or external publicity. Once your paper has been scheduled you will receive an email confirming the publication details. This is normally 3-4 working days in advance of publication. If you need additional notice of the date and time of publication, please let the production team know when you receive the proof of your article to ensure there is sufficient time to coordinate. Further information on our embargo policies can be found here: https://www.nature.com/authors/policies/embargo.html

To assist our authors in disseminating their research to the broader community, our SharedIt initiative provides you with a unique shareable link that will allow anyone (with or without a subscription) to read the published article. Recipients of the link with a subscription will also be able to download and print the PDF.

As soon as your article is published, you will receive an automated email with your shareable link.

If you are active on Twitter/X or Bluesky, please e-mail me your and your coauthors’ handles so that we may tag you when the paper is published.

You can now use a single sign-on for all your accounts, view the status of all your manuscript submissions and reviews,

access usage statistics for your published articles and download a record of your refereeing activity for the Nature journals.

Please note that you and any of your coauthors will be able to order reprints and single copies of the issue containing your article through Nature Portfolio's reprint website, which is located at http://www.nature.com/reprints/author-reprints.html. If there are any questions about reprints please send an email to author-reprints@nature.com and someone will assist you.

Please feel free to contact me if you have questions about any of these points.

Best regards,
Arunima

Arunima Singh, Ph.D.
Senior Editor
Nature Methods

** Visit the Springer Nature Editorial and Publishing website at www.springernature.com/editorial-and-publishing-jobs for more information about our career opportunities. If you have any questions please click here.**

Open Access This Peer Review File is licensed under a Creative Commons Attribution 4.0 International License, which permits use, sharing, adaptation, distribution and reproduction in any medium or format, as long as you give appropriate credit to the original author(s) and the source, provide a link to the Creative Commons license, and indicate if changes were made. In cases where reviewers are anonymous, credit should be given to 'Anonymous Referee' and the source.
The images or other third party material in this Peer Review File are included in the article's Creative Commons license, unless indicated otherwise in a credit line to the material. If material is not included in the article's Creative Commons license and your intended use is not permitted by statutory regulation or exceeds the permitted use, you will need to obtain permission directly from the copyright holder.
To view a copy of this license, visit https://creativecommons.org/licenses/by/4.0/

Answer letter for our manuscript

Dear Arunima,

I am coming back to our manuscript “Times are changing but order matters: Transferable prediction of small molecule liquid chromatography retention times”. Below, you will find answers to the questions and issues raised by the reviewers, as well as the changes we have made to the manuscript. Reviewer comments in blue, changes to the manuscript in green. Since this is almost a complete rewrite of the manuscript, we did not highlight the changes in the manuscript.

You will see that, while they find your work of some potential interest, the reviewers raise several serious concerns about the advance your methodological approach represents over available methods and about its broad applicability at this stage. They also question the novelty and evaluation of the approach as presented in the paper. We think that these criticisms are sufficiently important as to prevent publication of your work in Nature Methods.

The main critique point of the reviewers is that **the presentation of our results was really, really bad**. Looking at the manuscript from a distance, we must agree with the reviewers. We fell for the trap of ordering our results in the order in which we obtained them, instead of logically. Following the reviewers’ advice, **we have massively restructured and rewritten our manuscript, and performed numerous additional experiments** that prove the superiority of our approach. We now better explain the advantages of our method both in the results section and in two Supplementary Notes.

- Regarding the **advance of the method**, our method is the first ever to predict retention times for a chromatographic setup for which we (the user, the world) have not measured a training dataset with authentic standards. All 2000+ papers published so far train a machine learning model on a dataset from authentic standards, measured on the machine where the model is then also applied. Our method does not need that; we only require a few (30) putative identifications via library search. Second, **the performance of our method improves when more data become available**: For every 30 datasets in RepoRT, we find that our error rate drops by roughly 0.1 percentage points. You may think that this is to be expected for a machine learning method; yet, we also demonstrate that pretrain/fine-tune methods do not improve in performance with more data. Finally, **pretrain/fine-tune methods substantially overfit to the target datasets**. Using a realistic split of the data results in much worse predictions, see below. In contrast, our method does not suffer from this problem, and we now outperform all other methods for all but one datasets. We have added a Supplementary Note describing the advantages of our method over the current state-of-the-art.
- For the **novelty of our method**, we believe this is indeed a game changer: After thousands of published papers that presented methods for retention time prediction, all of which required you to measure thousands of reference compounds on your instrument and train a machine learning model yourself, **our approach can be applied to any dataset measured on any instrument, without the need of you measuring references** or training a machine learning model. Our machine learning

model is the first that can generalize between chromatographic columns; we can do predictions for columns that will only be developed in the next decades, as well as for compounds or even compound classes that are not known today. This is a massive breakthrough. In contrast, all existing methods require that you first measure a comparatively large number of compounds (hundreds) on your instrument. Besides the time, work and money (buying hundreds of authentic standards is not cheap), you also have to redo this every time you change any parameter of your chromatographic setup. If someone wants to re-analyze your data, you have to save the model for retention time prediction together with the data. If you want to analyze data from somebody else (we now have repositories of such data), you are basically screwed.

- For its **broad applicability**, we have added a paragraph and extended the Supplementary Note, describing applications that you can do with our model, but that are not possible (or highly tedious, meaning impossible in practice) with any of the existing models. Just to mention one important example: It is basically impossible to integrate previous retention prediction models into *in silico* methods such as SIRIUS and CSI:FingerID. How should that even be done; is the user supposed to upload his/her model that we then use for the retention time predictions? That sounds like an implementation and security nightmare. Will the user have to provide a web service for us to get the predictions? Or, are we (as the CSI:FingerID providers) supposed to train an individual model for each user? In which case the user first has to upload a reference dataset with annotations, before s/he can start the actual analysis? Do we have to store the model somewhere, to ensure reproducibility? We have no idea how that could possibly work in practice, and we must assume that nobody else has, since no retention prediction models have been ever integrated into any *in silico* method. In contrast, we plan to integrate our 2-step method into SIRIUS in the near future, and we assume that other developers of *in silico* methods will do so, too.
- Both reviewers indeed question our **evaluation**, and their points of criticism are valid, helpful and smart. We have performed numerous additional evaluation experiments, demonstrating the power of our approach. This includes “our method gets better with more data, previous methods do not” and “previous methods only shine in evaluations because these evaluations did not avoid overfitting”.

Following your advice, we have also added a sample application of transferable retention time prediction using an experimental dataset, where we showcase some of the possibilities described in the Supplementary Note.

Our manuscript is currently too long. Maybe, the complete statistical part (now the second part, in particular the second reviewer did not like it) could be removed. It is not important for the 2-step method. But we are also happy to keep it in.

Best regards, Sebastian Böcker (for the authors).

We want to explicitly thank the reviewers for their excellent comments, questions and suggestions. Following their advice has substantially improved our manuscript. We have to excuse ourselves (if that is possible) for the state the manuscript was previously in.

Reviewer #1 (Remarks to the Author):

The authors present a method to predict retention time of molecules on different chromatography setups. They present a 2-stage solution where a GNN model is trained to predict a retention order index and then a model to predict the retention time using the predicted retention order index.

Detailed description of how prior works relate to the contributions of current works needs to be clearly stated in the introduction. The novelty needs to be highlighted to better place the work in context. The results also need to be put in context of other works.

We have substantially rewritten and reworked the manuscript. We hope that this now makes it clear what the novelty etc of our method are. The true story was indeed rather hidden in the previous version, sorry for that. We have rewritten the (now) three paragraphs on our contribution, in particular the advantages and applicability of our method. See the start of the Results section; we believe that this is where *Nature Methods* wants it.

Include in methods an explanation why a non-zero margin in the Margin Ranking Loss makes sense

We have chosen a non-zero margin for faster convergence, but forgot to justify this in the manuscript. We have included an explanation in the manuscript, and also performed an analysis on how the margin affects performance. See also the next answer.

Run a grid search for hyperparameter search (currently manually selected)

We have restructured our evaluation splits and added a grid search on a holdout portion to determine important hyperparameters. We find that overall performance is very robust. We describe the details in the manuscript, see in particular STab 10.

Include in methods an explanation for why you used a sigmoid in the final layer, why does ROI need to be a value between 0-1?

Well-spotted, thank you. There is indeed no theoretical justification why the ROI needs to be restricted to [0,1] and, hence, there is no need for the sigmoid layer. We changed the model accordingly, and also the description in the manuscript. The ROI now uses a much larger interval, see below. Also, the sigmoid layer resulted in ROI values that were supposedly artificially limited for certain datasets, leading to a “band” for ROI 1.0. Without a sigmoid layer, this artifact disappears, see below. Unfortunately, this change did not result in an overall improvement in prediction quality, at least for the statistical measures. Which tells us that evaluation statistics are not the whole story. In full, thank you, excellent suggestion.

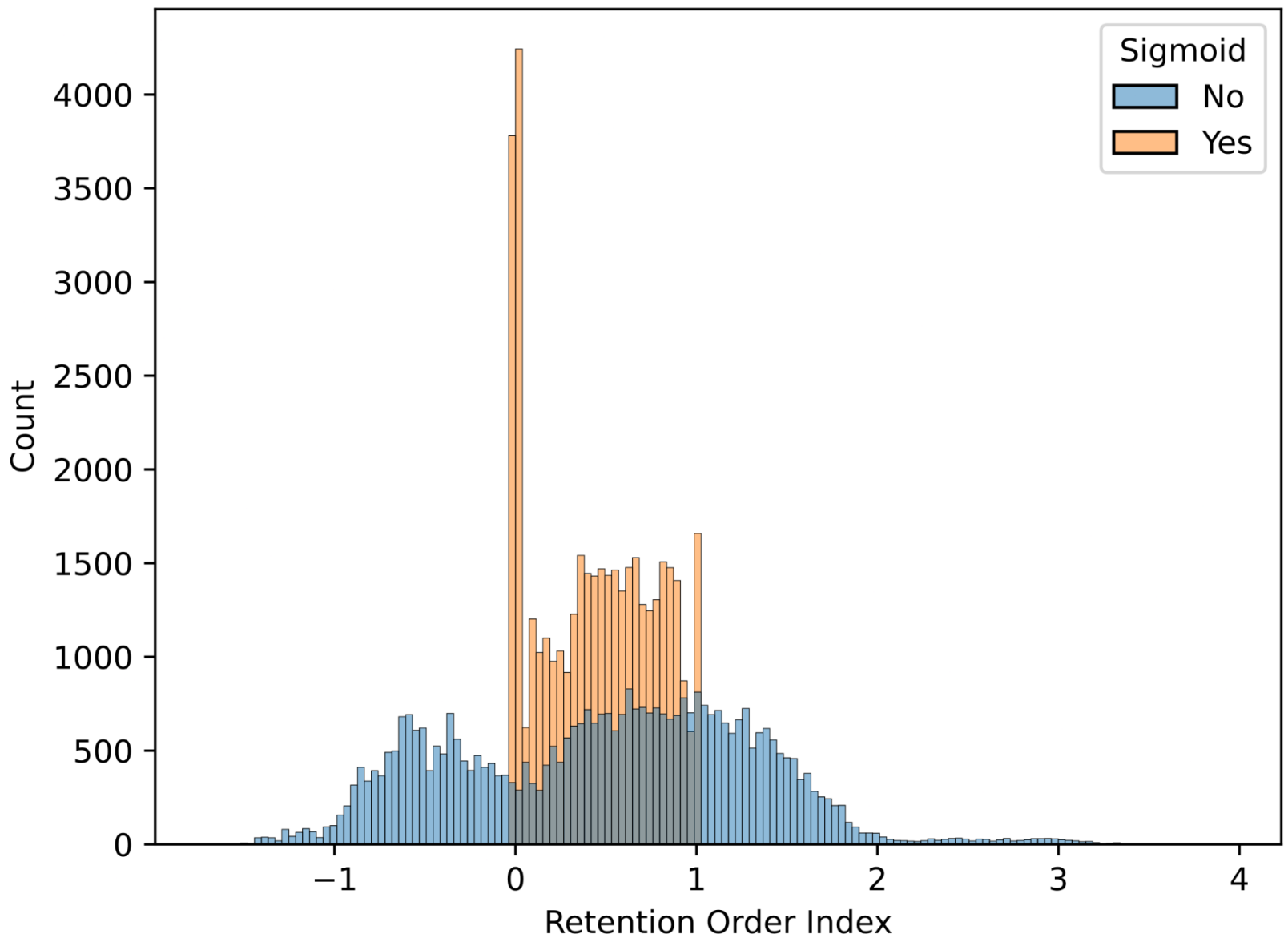

Distribution for Retention Order Index with and without final sigmoid layer

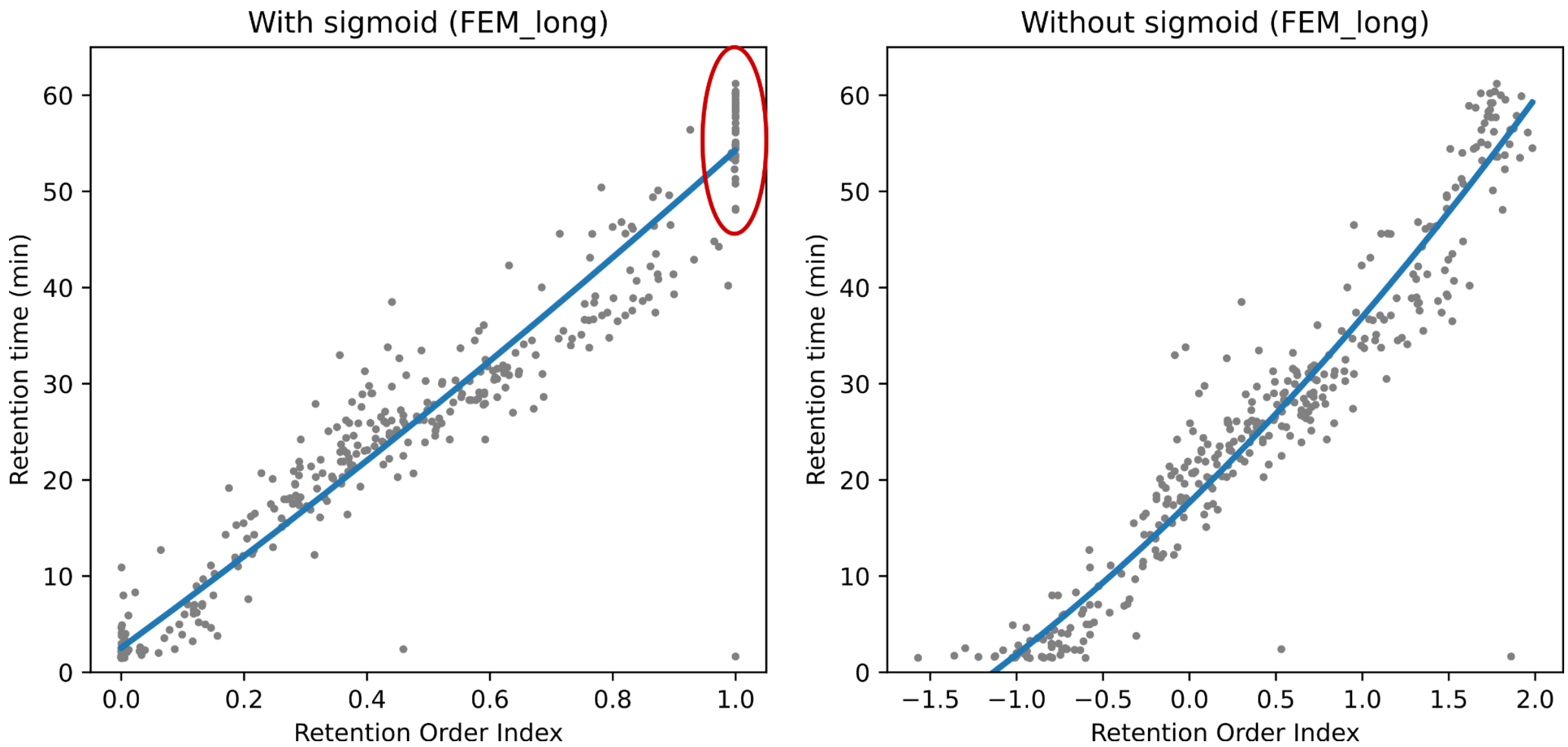

Without the final sigmoid layer (right), an artifact for high ROI values (left) disappears

Please provide a better description of the siamese network and how it is used in this work so readers can understand this model

We have rewritten and extended the description of our neural network architecture, in particular the differences between training vs. prediction phase, both in the text and the workflow figure (fully reworked).

Describe in methods how Tanaka and HSM vectors are obtained

We have added a short description to the results, and details to the Methods section.

Describe in the paper what data is contained within RepoRT

We have added two sentences to the results section: “For training and evaluation, we used data from RepoRT, a repository for retention time data plus metadata on chromatographic conditions. RepoRT contains thousands of unique compounds measured on tens of chromatographic columns using varying eluents, flow rates, and temperatures.”

“We also evaluated numerous modifications of model architecture and features, but doing so did not result in substantial improvements of prediction performance (data not shown)” – please explain in more detail

As suggested above, we have added details about a hyperparameter search to the manuscript. Beyond that, we have removed the sentence.

Make sure DeepGCN-RT,RT-Transformer and your own models are trained on the same data

RepoRT consists of hundreds of datasets, and retention times measured in different datasets are basically incomparable. A compound may have retention time 100s in the first dataset, and 500s in the second. To this end, we assumed that DeepGCN-RT and RT-Transformer could not reasonably be trained on RepoRT, **but we were wrong; it actually worked**. To account for the vastly different retention times between datasets, we invented (or maybe, this is known?) a new pretrain/fine-tune method: In addition to “single-pass pretrain/fine-tune” where all of RepoRT is used uniformly during pretraining, we also use “repeated pretrain/fine-tune” where the model is pretrained on a dataset, then refined on the next, which is then used as pretraining for the third, and so on. To our surprise, both variants performed mostly on par, and even better than pretraining on the (homogeneous and huge) SMRT dataset. We have added all details to the paper.

We find that DeepGCN-RT and RT-Transformer perform slightly worse than what has been reported in the literature. We attribute this to three inconsistencies in previous evaluations: First, the target datasets contained some duplicates (i.e. identical lines in the file, with identical compounds and identical retention times) and doublets (same compound, different retention times). Duplicates usually end up in different batches, and just have to be memorized by the model. Second, the authors did not exclude the void volume from evaluation. Clearly, you are free to use all compounds from the void volume for training your method; but the exact time that a compound elutes in the void volume is basically meaningless, and must not be used to evaluate a prediction. Third, different from what is described in the paper, the DeepGCN-RT code does not establish the number of training epochs via cross-validation; rather, it uses the best number it observes throughout training. We have corrected all of these issues; the effect is not dramatic, though.

Importantly, DeepGCN-RT and RT-Transformer are fine-tuned on the target dataset using cross-validation. Our 2-step method is not trained on the target dataset at all. In short, the other methods have substantially more (and more relevant) data to train on.

Notably, RT-Pred (Zakir *et al.*, 2025) was published in February of this year. Unfortunately, the paper fails to evaluate RT-Pred against DeepGCN-RT. Manually comparing numbers in the two papers, we found that RT-Pred does not perform better than DeepGCN-RT; furthermore, the source code of RT-Pred is not public. Hence, we did not include RT-Pred in our evaluations.

Explain in the paper why retention order prediction is a meaningful problem even if only ~7% of data are informative pairs

Retention order prediction per se has nothing to do with informatory pairs: such pairs have different orders in different datasets. Yet, the first task of a model is to predict the order of all pairs, whether they are informatory or not. We added “We stress that predicting ROIs would still be a sensible and important task if there were zero informatory pairs; in this case, we could simply ignore chromatographic conditions” to the manuscript.

The low fraction of informatory pairs is basically an artifact of considering compound **pairs**. If 10% of the compounds change retention order by 10% of the run length, then only 1% of the compound pairs are affected. We now mention this fact in the text, and added a Supplementary Note with details. Next, when more datasets with more chromatographic conditions are added to RepoRT, then more pairs will differ between at least two datasets and, hence, become informatory. We have added, “Numbers on monotonous and informatory pairs reported below are solely a snapshot of the current RepoRT repository. When new datasets are added, the number of informatory pairs in existing datasets can only increase, because a single dataset with deviating order is sufficient for that.”

We also added a comparable text for setup-characteristic pairs. At present, these make up only 1.26% of pairs; but we are convinced that they are indeed the most interesting ones, both from the theoretical and the practical side.

If considering compound pairs leads to statistical artifacts, why did we nevertheless use it? In fact, we found a statistic that is linear in the number of compounds, not in the number of compound pairs: This is the number of compounds you have to delete so that both orders are identical. Yet, numbers that came out of this analysis were really confusing, even for us, because tiny changes in retention order may force you to delete 50% of the dataset. (The best method was still the best method, though.) We decided to remove this analysis before submission. This statistic may become handy in the future, though.

Explain in the paper why an accuracy below 60% on Setup-characteristic cases is good compared to 50% accuracy expected with random selection

Setup-characteristic pairs are those that deviate from the majority of datasets. Any reasonable machine learning model will mainly try to predict the majority vote and get few, if any, pairs correct that deviate from the majority. Hence, the random method will get 50%, but a good model not aware of chromatographic conditions may be at 0%. We have clarified this in the manuscript.

Explain in the paper why people should use your model if the retention time predictions have lower accuracy than DeepGCN-RT

We have added a paragraph on the advantages of our method, plus a Supplementary Note with details. We find that our performance is now on par even for the original (uniform) cross-validation split. Most importantly, using a realistic evaluation of the pretrain/fine-tune methods instead of the generally frowned-upon uniform split of compounds, **we now strongly outperform all existing methods** for most datasets, and perform roughly on par for the remaining. We have added this evaluation to the manuscript. Finally, we have added

an evaluation showing that our method's performance improves when more data become available, whereas pretrain/fine-tune methods do not.

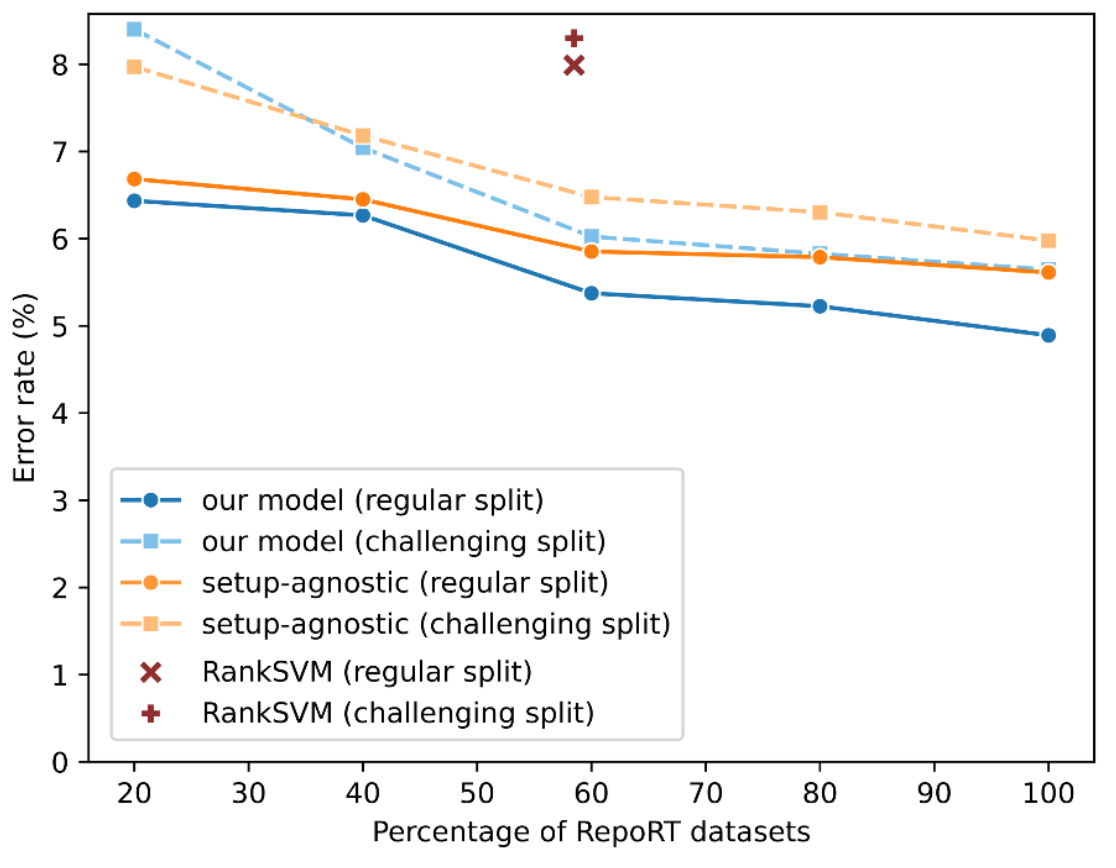

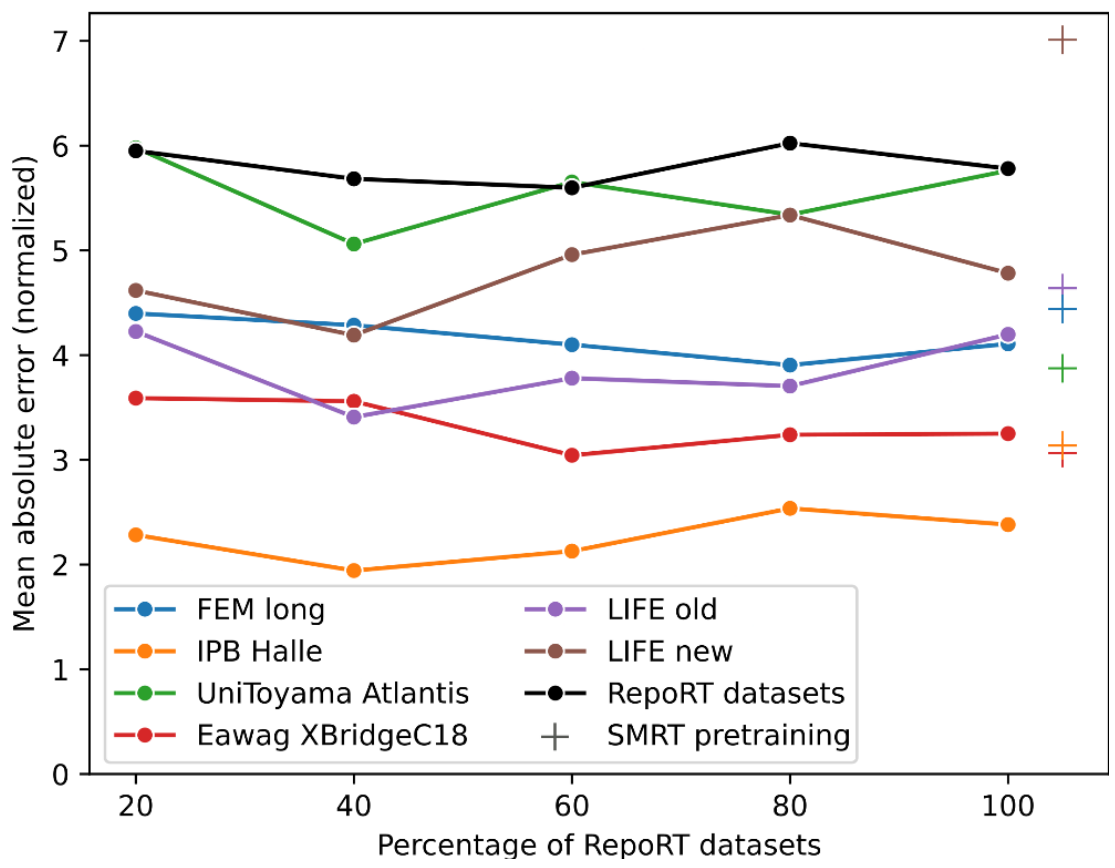

Left: Our models' performance improves when there is more training data. Apparently, we are still in the linear phase of performance gain. As a back-of-the-envelope estimate, the error rate will drop to about 3% (regular split) when the amount of available training data doubles. This is clearly too optimistic, but gives an idea. Right: DeepGCN-RT does not improve when there is more pretraining data. Some errors go down, some go up, and for some, there is no trend. Assuming that the amount of (pre)training data will grow is very realistic; for fine-tuning data this is not realistic, see Supplementary Note 2. We normalized errors by length of the run, see below.

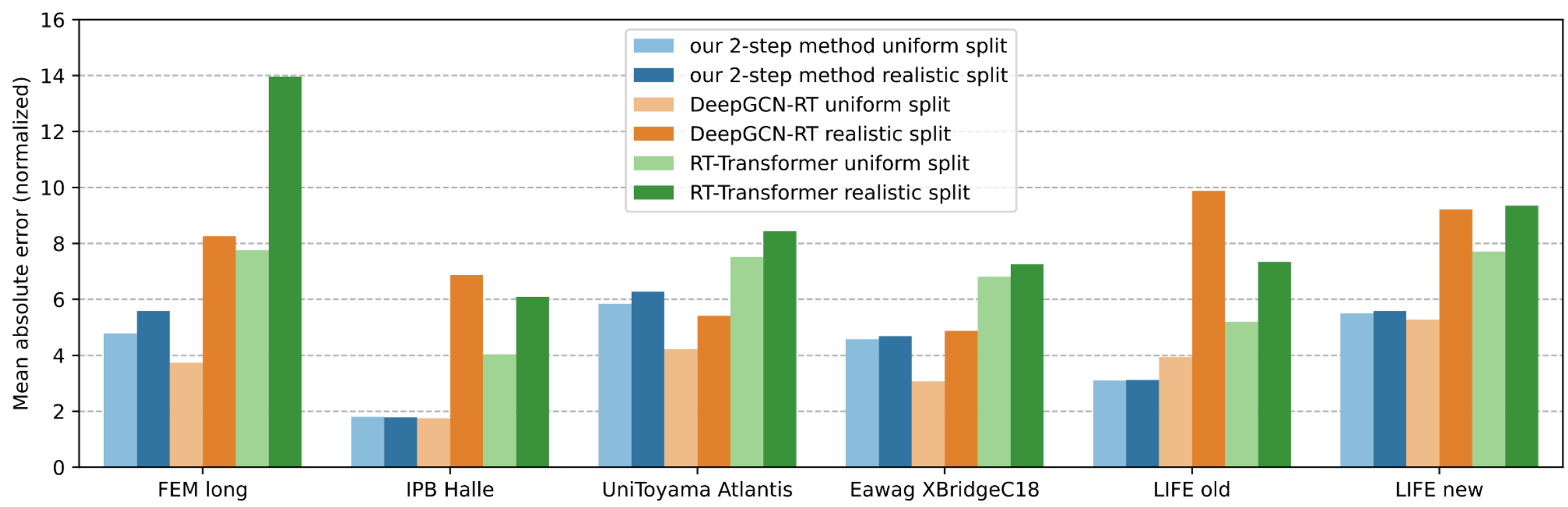

Uniform splitting drastically overestimates the expected performance of machine learning models. This is well-known in the community, and it is somewhat surprising that all previous papers on RT prediction nevertheless use a uniform split. In the previous version of the manuscript, we only conjectured that there might be overfitting; we have now executed evaluations to prove it. We use a recently suggested split based on Maximum Common Edge Subgraphs, that agrees well with chemical intuition. For this realistic split, we observe a substantially worse performance for pretrain/fine-tune methods. Notably, evaluation results for the realistic split are still too optimistic for pretrain/fine-tune, as we explain in the manuscript. **Our 2-step method now clearly outperforms pretrain/fine-tune for all but two datasets**, and is roughly on par for the remaining. For one of the datasets, our method suffers from a small but notable performance decrease; this is actually another evaluation artifact, but this discussion is probably deviating too much from the main story.

Include other retention time prediction evaluation metrics, prior works (DeepGCN-RT, RT-Transformer) evaluated on including R^2, MedAE, etc.

We now report these metrics in Supplementary Table 5. Results all show the same trend. For the realistic split, we outperform all other methods by a large margin for all metrics.

In our evaluation, we now normalize errors by the length of the gradient. Clearly, prediction errors in a 12 min run are much smaller than in a 180 min run; normalizing by gradient length takes care of this problem. We already mentioned this problem in the previous version of the manuscript. Without this normalization we cannot integrate statistics from different runs in one number (Supplementary Table 5) or one plot (Figure 4). We argue that normalizing by gradient length is more reasonable than reporting mean relative error or mean absolute percentage error: An error of 60 seconds is equally important at the beginning and at the end of the run.

In the paper, add a description for how DeepGCN-RT and RT-Transformer work

We added a sentence for each method.

In the paper, add a description for how prior models were fine tuned for the target dataset, what data was used for fine tuning vs testing

We added information about the general pretrain/fine-tune setup to the Results section. We added a new subsection to Methods, describing how these models were trained and evaluated.

Larger fonts across all figures

We have better distributed the plots and subfigures into figures; for example, Figure 1a is now a separate figure in the supplement. We assume that this has fixed the problem of tiny fonts.

The number of compound pairs under different thresholds and the number of conflicting pairs should be placed in a table

Done (Supplementary Table 9) and removed from the text.

STable 3 should be moved to the main text

We have moved the table. We have removed the results for the "additional training data" (ATD) model, because we now have a separate ablation study on the influence of removing/adding training data (Figure 2d).

Explicitly state where the weight function f(x) is used

We have added further details about the weight function to the Methods, and explained where it is used in the Results.

Reviewer #2 (Remarks to the Author):

The primary contribution of this paper is to develop a Graphical Neural Networking with message passing that is able generalise retention time prediction across samples analysed

under different conditions. The method appears relatively sound and there is some analysis to show why it might be necessary. I am not completely convinced by the novelty improvements offered by the work. No comparison is done for the actual situation where this method would be used, so its unclear how much it is better than existing non-transferable methods, although presumably these results would be positive. I am also not sure its contribution compared to say ref18, or other competitor methods mention, as the GNN+MP is not novel in itself. My conclusion is that the method is probably nice, but I am not convinced that the contribution is enough for this level of paper.

We have added two paragraphs and Supplementary Notes to the manuscript on the applicability and the advantages of our method. In short, our model can be applied to any dataset measured on any instrument, without requiring a user to measure references or train a machine learning model. Just to mention a single application where our model can be used, but existing approaches including pretrain/fine-tune cannot: It is extremely challenging to integrate previous retention prediction models into *in silico* search methods, such as SIRIUS and CSI:FingerID developed in our lab. Is the user supposed to upload his/her model that we then use for the retention time predictions? That sounds like an implementation and security nightmare. Will the user have to provide a web service for us to get the predictions? Or, are we (as the CSI:FingerID providers) supposed to train an individual model for each user? In which case the user first has to upload a reference dataset with annotations, before s/he can start the actual analysis? Do we have to store the model somewhere, to ensure reproducibility? Notably, the group of David Wishart published a web service for RT predictions in February (Zakir *et al.*, *J Chromatography A*, 2025); this would at least take away the burden to train and store your own model, but comes with the usual downsides (see Supplementary Note 2). We have objections that this will work in everyday practice.

We conjectured in the previous version of the manuscript that the good performance of pretrain/fine-tune methods might be due to overfitting. In this version, we prove this: Doing a realistic split of the training dataset, our method now clearly outperforms all existing pretrain/fine-tune methods (Figure 4). We have changed the manuscript accordingly.

Our machine learning model is the first that integrates an encoding of the chromatographic column and, hence, can generalize between different columns. Beyond that, we do not claim that the machine learning model is particularly noteworthy; in fact, coming up with (yet another) machine learning model for retention time prediction would be somewhat underwhelming. Literally hundreds of papers have done that. The novelty of our method is the idea to split the prediction: The machine learning model only learns to predict a retention order index; the actual retention time prediction is done by mapping those ROIs to RTs using a second-order polynomial. **This 2-step procedure allows us to predict retention time for any chromatographic setup**, without having ever seen any data from that setup during training of the model, even for a setup that has not been measured by mankind so far. We have considerably rewritten the manuscript, to clarify this point.

Grammer and language wise, the paper is fine. However, beyond that I find it poorly written, lacking consistent motivation for the reader throughout the paper, and lacking adequate comparison to existing methods. On second read it becomes clearer, but it would need a substantial rewrite in my opinion even if the scientific contribution is judged to be sufficient. It took be to read 10 pages and 2 pages of comments and questions before I fully understood

what the method was and why it was useful. Tables and figures generally need improving as well. Despite some of these comments, I could see a possibility of accepting the paper, but it would need a substantial rewrite, more analysis with motivated explanation, and more links to the existing literature.

We agree that the manuscript was written poorly. We fell for the trap of ordering our results in the order in which we obtained them, instead of logically. Sorry about that. We have reordered and rewritten large parts of the manuscript, and hope that it is clearer now. Many thanks for the numerous helpful comments!

For the literature, we think we have cited everything that is remotely of interest for the manuscript, but we now describe the existing approaches in more detail. If we have missed a particular paper, we are happy to add that as well.

Introduction – the introduction gives a nice introduction to some of the background (and is well written), but strangely (at least in my eyes) does not mention the work in this paper, or more crucially give the reader any idea how this work fits with the existing research

Nature Methods wants this to be the first paragraph of the results section, if we are correctly informed. We have substantially extended this description, mostly on the applications and advantages of our method, see other comments, and we have added and extended Supplementary Notes on the applications and the advantages of our method. We are happy to incorporate further suggestions.

Figure 1a – there is not enough explanation here to do more than guess what is going on. Would suggest making it bigger with a more detailed caption (e.g. move side titles to top titles, double width and height of images, add an explanation for each image). Maybe need to make arrows thicker as well. Images are great if bigger and with explanations

We made a separate supplementary figure out of Figure 1a (now Supplementary Fig. 8). We have added explanations in the caption.

Results: ‘see below’ – completely unclear what this means

Removed.

Results: ‘dataset boundaries’ – completely unclear what this means

Removed.

Results: I don’t see how ‘chromographic conditions to be “nominally identical”’ leads to ‘Accordingly… is futile’ – you define something, then that leads to something being futile, doesn’t really make sense.

Removed. Yes, that did not make any sense.

Results: ‘highly restricted compound’ – hard to follow this, but probably relates to previous comment

Removed.

Results: ‘such deviations’ – what deviations?

Replaced by: "Variations may be due to different void volumes, gradient delay volumes and related parameters of the LC systems, which in turn depend on the used capillaries and numerous other parts of the LC, or alterations of the column due to repeated use. Clearly, such variations are the rule and not the exception."

Results: Is this second paragraph meant to be an introduction to the results section? Why is it given a summary of results we have seen yet of studies we don't know what they [do]? How is the reader meant to have any idea what is going on? It needs to be written in a way that explains what you want to do. Maybe it would make sense if there was a summary saying what the paper was about in the introduction but currently it makes no sense.

We have substantially reordered the manuscript, and the first three paragraphs of the Results section now explain what the paper is about. Following *Nature Methods* politics, we made this the first paragraphs of the Results.

Results: Be more precise than 'machine learning model', this would be equivalently vague to describing all the data as being from a 'chemistry experiment'

We assume that this comment refers to the sentence, "we present a machine learning model to predict retention order indices." This is the only comment where we have to disagree: The actual machine learning model is of comparatively little importance. (The important ideas are: predicting ROIs instead of RTs; integrating chromatographic conditions into the machine learning model; training the model as a Siamese network; mapping ROIs to RTs via a low-degree polynomial. We hope that the rewritten manuscript gets these messages across.) In fact, we evaluated other model architectures; it turns out that they all perform basically on par, so we decided to choose a relatively simple one. Simple means, less chance to overfit. We could add "relatively simple" to the sentence, but you are free to use a complicated one instead. Indeed, we hope that others will come up with better models for ROI prediction, be those models simpler or more complicated, as this will only benefit our 2-step approach. We now describe the model in more detail at a later point of the manuscript.

Conflicting pairs as a measure of disagreement: vs. to versus

Done.

Conflicting pairs as a measure of disagreement: This section is lacking some motivation for the reader, probably as its missing an introduction type chunk of text at the start to give context

We added: "We now analyse what metadata about chromatographic conditions are required for the analysis of small molecule retention in a dataset. [...] Naturally, we are interested in a minimum set of parameters: The fewer fields to fill in, the likelier it is that scientists include this metadata when uploading a dataset to a repository."

Conflicting pairs as a measure of disagreement: The final conclusion is essentially a list of numbers. Presumably this motivates the results of the remaining sections, but again the reader is not told that and has to guess. I am not convinced a list of numbers with little motivation is Nature Methods worthy, sorry!

We have substantially rewritten the sections about conflicting pairs, and hope that the intention of each step of our analysis now becomes clear. In short, changes of retention order are a rather common phenomenon.

Influence of chromatographic conditions on retention order: Same comment as before, needs clear introduction and motivation, and needs linking to your actual methods

Done.

Influence of chromatographic conditions on retention order: So far both results sections are essentially summary statistics and results, not particularly inspiring

As described above, we have tried to motivate each step of our analysis in the new version. We have also moved numbers to Supplementary Tables etc..

Influence of chromatographic conditions on retention order: Figure 2 – quality leaves a bit to be desired. Figures are already very small, then there is small writing and thin lines. Its acceptable at 200% zoom on my computer, but only just.

Sorry about that, we restructured the figure to make the subfigures much larger.

Predicting retention order indices: Please explain how the graph structure is set up for these predictions, and why intuitively that might be helpful. Is it the graph structure in Fig 3a, e.g. the metabolite, if so then intuitively how is this represented? – there is more information in the methods, but a little more is needed here

The metabolite is indeed encoded via its graph structure. We have added an explanation of how the molecules and the chromatographic conditions are represented in the prediction model.

Predicting retention order indices: 'To predict the retention order, the two ROIs are subtracted' – why is this necessary? Is one number being bigger/smaller than another not sufficient for this?

Comparing which number is larger, is exactly what is happening; we have clarified the description of what constitutes an correct/incorrect prediction and how that affects the loss in the manuscript.

Predicting retention order indices: Is the key advance here compared to existing methods that you combine the experimental conditions with the graph representation? I think this section could do with explaining more why you are different to existing methods. The introduction to the paper, did a nice overview of methods which appear to do the same task as what you describe. You compare to one, presumably much simpler, method from 7 years ago, surely there are simpler NN / GNN approaches that would be more modern, speedier, and comparable performance wise.

We evaluated our method against exactly the same state-of-the-art model with exactly the same training procedure and the same training data; only the chromatographic information was removed. The old method (RankSVM) is not simpler and in fact, the maths behind it are somewhat more "elegant"; it is just a different type of machine learning. Neural networks are

known to better handle large amounts of training data; indeed, this appears to be the case for RepoRT. We have modified and extended the text, to make all of this clear.

In addition, we have evaluated another “classical” descriptor-based method (RankNet) that can predict retention order indices, and added results to the manuscript. Performance is slightly worse than that of the ROI model excluding chromatographic conditions. We find it reassuring that different architectures result in very similar evaluation statistics. In fact, numerous other machine learning architectures will reach a similar performance and, hopefully, an even better performance in the future. That would be great, **as our 2-step approach would then allow us to predict better retention times**, too. See our answer below about the advantages of our method.

Predicting retention order indices: What is the relationship of your work in this section to ref18? They must be doing a similar thing given the title?

We are not sure which reference is meant here. Osipenko *et al*. [18] present a “regular” pretrain/fine-tune model, which performs worse than the two we discuss in the paper. Only one paper besides Bach *et al*. mentions “elution order”, namely [26]; but despite the title, the model in [26] also predicts retention *times*. To the best of our knowledge, we cite all papers that discuss the prediction of retention order.

Mapping retention order indices to retention times: You mention two competitor methods (RTTransformer and DeepGCN-RT). Based on the name alone, these must be relatively similar concept at the basic ML level. Please give some intuition around what the similarities and differences are to your method. What is the intuitive reason why your method performs better and are these methods designed for the same task or for a fixed experimental setup?

We added substantial details on the differences and advantages of our 2-step method over classical pretrain/fine-tune methods, see the start of the results section and Supplementary Note 2. The main differences are our 2-step procedure of predicting ROIs, then mapping to RTs, and the ML model that takes into account chromatographic parameters. We hope this is clear with the new version of the manuscript. We also added two sentences on the architecture of the two models. These are “standard” pretrain/fine-tune models for predicting retention times. As noted, the actual model architecture used for predicting ROIs is not of that much importance; in fact, we conjecture that when even more data become available within RepoRT, transformer-based models may outperform the GNN architecture. As mentioned, our 2-step approach for ROI prediction would benefit from that, whereas pretrain/fine-tune models for RT prediction cannot.

Mapping retention order indices to retention times: Table 1 – I struggled to work out which your methods even were. I have concluded its all but the top two? Then it’s a load of different scenarios? My understanding is then that they have partially seen target data and you have not and that is why your methods are worse? If so, then essentially the results aren’t comparable between the first two rows and the rest? Is there some form of fair comparison that you can do? What would happen if you naively used the two existing methods on data they had not seen before, or is the point that this would just not work at all? There must be some methods that you can compare to in a fair way, where both methods have not seen the target data. Apologies if I have misunderstood here, I have struggled with the writing style and organisation of your paper.

The table was confusing, as it mixed model comparison with an ablation study for our model. We made a separate table for the ablation study. We stress that the comparison is absolutely fair. In fact, previous evaluations (uniform cross-validation split) were “unfair” because they rewarded overfitting and memorizing.

We have added an evaluation where we use the DeepGCN-RT model pretrained on RepoRT (this one performs better, see above) to predict retention times in a target dataset **without fine-tuning**. This is equivalent to fine-tuning on some target dataset A, then applying the method to dataset B. This cannot work and indeed, it does not work, see Supplementary Table 5, top two rows. We observe a coefficient of determination below zero, meaning that simply returning the average retention time in the dataset would have performed better than this predictor.

Mapping retention order indices to retention times: Having read further I now understand that matching performance would be a very good thing. This needs to be made clearer to the reader throughout, I think you are lacking the motivation in the writing, which I think is actually very sound, just not explained. An additional comparison that shows the other methods are not transferable between datasets would be beneficial, even if it shows that your results are much better

We agree that proper explanation was missing; we have rewritten our manuscript substantially, to explain the numerous benefits of our approach. See above for the discussion on applying a pretrained model without fine-tuning or, equivalently, using a model fine-tuned to the wrong target dataset. This highlights the central disadvantage of these methods, which by design are not transferable without additional training.

Mapping retention order indices to retention times: least squares optimization – least squares is the solution method and therefore does not need optimising (instead calculating). You could optimise the loss of an MSE which would be essentially the same.

We have corrected this in the manuscript.

Training retention order index prediction models: is 10 epochs enough for any form of convergence, seems unlikely. Have you explored what the effects of changing this is?

We use 500,000 sampled compound pairs per epoch, generated from approximately 30k compounds, therefore 5M compound pairs are seen by the model in 10 epochs. We conjecture that this is enough data for convergence; exploring performance empirically for a higher number of epochs we observe notable overfitting for more epochs, with the error rate on unseen datasets decreasing after more than 20 epochs, see below. As the loss on extra validation data from the training datasets does not reflect this overfitting, therefore not allowing early stopping, and we decided against excluding whole datasets only to determine the optimal number of epochs in order to keep the number of training data high, we opted for the manually determined value of 10 epochs for all models. We have added an explanation for our decision for 10 epochs to the manuscript.

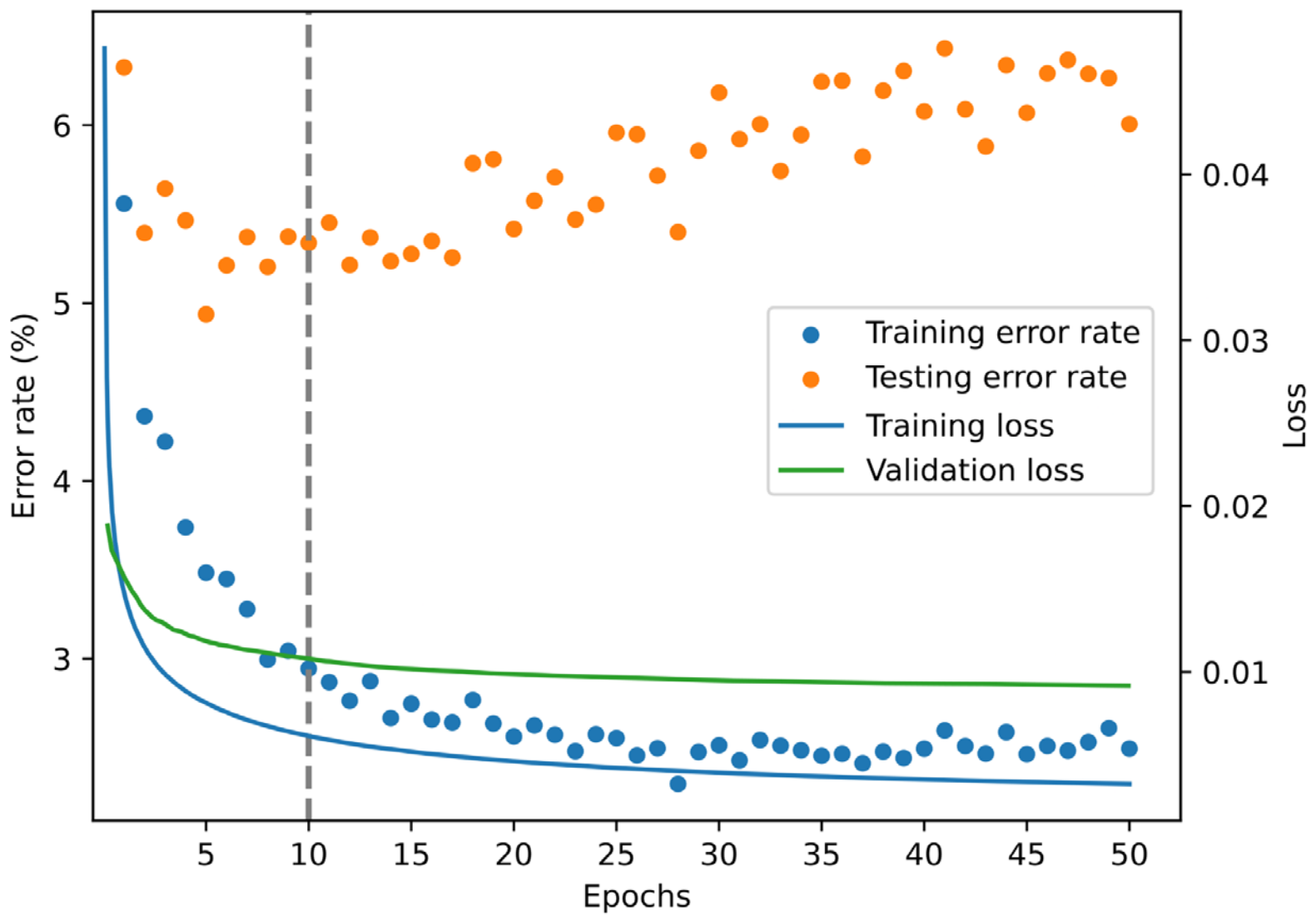

Error rates for train and hold-out datasets, exhibiting noticeable overfitting for high numbers of training epochs.

Code looks sensible and well organised

Thank you.

Answer letter for our manuscript

Dear Arunima,

I am coming back to our manuscript “Times are changing but order matters: Transferable prediction of small molecule liquid chromatography retention times”. Below, you will find answers to the questions and issues raised by the reviewers, as well as the changes we have made to the manuscript. Reviewer comments in blue, changes to the manuscript in green.

For the comment regarding conciseness by reviewer #1: We cannot do that, unless the reviewer tells us what exactly (s)he believes is not concise. We are also not sure if the reviewer really wants more conciseness; the individual changes the reviewer asked for, required explaining things in more detail, making the descriptions longer, not more concise. We are happy to make further individual changes if the reviewer tells us what. Otherwise, the comments of this reviewer are helpful and positive, and we implement all changes.

The comment of reviewer #3 that the text “still remains inadequate” leaves us puzzled. What is a wordy text? What is a weak structure? What details are unnecessary? What does it mean that the text introduces new problems and concepts “too early”? What is “streamlining the manuscript” supposed to mean? We will be happy to further improve the quality of the manuscript together with the copy editor, but these comments are not helpful in doing so. This is not the first manuscript that we are writing. We think it is very well written, given that the audiences that will read it are rather heterogeneous (analytical chemists, chemo- and bioinformaticians, some people from machine learning etc.).

Both reviewers #1 and #3 have issues with the second part (statistical analysis of what chromatographic parameters result in retention order changes) of the manuscript, as this is causing some confusion. We do not think that we should make this part any shorter. In fact, we have already removed large parts that are interesting, too, such as the influence of the flow rate on retention order. If this is causing only confusion and the reviewers do not like it, it might indeed be best to cut it out.

We are well aware that certain details and facts are now mentioned repeatedly throughout the manuscript. For example, the fact that pretrain/fine-tune methods need training data from the target system, whereas our method does not, and that **2-step is the first method for transferable retention time prediction**. For us, these facts were clear from the introduction. We added more details because reviewers 1 and 3 had issues understanding these facts and their implications. So, to make the manuscript understandable for readers, should the repetitions probably remain? We leave that up to you and the copyeditor.

Reviewer #1 (Remarks to the Author):

overall great improvement in the clarity of the paper. Because this was a major rewrite and the authors chose not to highlight their changes, I reviewed it afresh. Here are the comments.

Thank you. We also wanted to say “thank you!” once more for the comments from the previous revision, which indeed helped massively to improve the readability of the manuscript. Since a huge portion of the manuscript was moved or rewritten, we refrained from explicitly highlighting changes.

1. Biggest concern is the wordiness of the paper. Please try to re-write for conciseness. Some examples are listed below.

We were unable to spot any places where we could be more concise. We are happy to make additional changes that the reviewer pinpoints us to.

2. Justification of the importance of the retention problem is not clearly articulated. Please provide better explanation why this problem is important and how it helps with elucidation and how it offers a new paradigm different than the existing one. The # of hits in google is not sufficient justification that it is an important problem. Unfortunately, the application does not show until the supplementary. Please move parts or some into the main text to stress the importance of this technique

Importance and applications were covered in the second paragraph of the results section. We have further extended this section and rewritten the paragraph on “what is transferable RT prediction” (see below) to make clear why transferable retention time is a game-changer. We now also mention repeatedly that existing methods cannot do transferable retention time prediction; 2-step is the first such method.

3. Please provide in the manuscript an intuitive explanation of “Retention order is known to be much better conserved than retention time”

Retention time is influenced by numerous factors that do not affect the retention order, e.g. gradient length, length of connection capillaries between column and detector, etc. We have added the following sentence to the manuscript: “Using a longer capillary between the exit of the column and the entrance of the mass spectrometer will cause a shift of retention times for all compounds, but does not change their elution/retention order (Supplementary Fig 7 ef).”

4. This sentence is not clear: “This differs from predicting retention indices mentioned above, where we first choose a particular index type, then try to predict it.” Please re-write this sentence and others for clarity (not sure what is mentioned above, retention times?)

We changed this to, “This differs from the computational task of predicting a retention index: There, one would decide for one particular retention index type (based on specific chemical indexing substances) upfront, then transform all measured retention times into retention index values, and finally, train a machine learning model that predicts exactly this retention index type.”

What other sentences? We believe that we have described this important yet highly non-trivial difference as detailed as possible, while staying relatively concise.

5. Please re-phrase: “and this is extremely unlikely to happen in the future” - just seems unnecessarily pessimistic considering it is not the main focus of the paper.

Done. The statement is nevertheless true, and we doubt that anybody working in metabolomics would object to it. It is also not pessimistic: The diversity of available column chemistries is actually an advantage of liquid chromatography, making it a versatile tool for metabolite analysis.

6. You introduce a new term, “Transferable retention time prediction”, but it is not defined anywhere

Indeed, this was only defined in the abstract. We have added an explanation to the beginning of the Results section: “We present a method for transferable prediction of retention times, meaning that our method can do predictions for an arbitrary chromatographic setup, without the need of training data from that setup.” That is, in one sentence, the message of the paper: Our method does predictions for any column, without training. As noted above, we now more clearly state that it is the first such method.

7. In the manuscript, add a description of the SMRT dataset, p. 9, and put in context for the reader - I just now know it is larger than all others.

Done, added “SMRT, a large dataset of retention times (80,037 compounds), measured on one chromatographic setup.” In the Methods (“Evaluation of DeepGCN-RT and RT-Transformer.”) we contrast it to RepoRT, there we added “For RepoRT datasets, the distribution of compounds follows much more closely that of known biomolecules than this is the case for SMRT.”

8. There is a “challenging split” and a “realistic split”. Please add a couple of sentences to justify the use of these labels - used in prior publications? I like the “maximum challenge” terminology!

Unfortunately, “challenging split” and “realistic split” refer to two different concepts. We therefore changed “regular split” and “challenging split” to “regular dataset split” and “challenging dataset split”. In contrast, “uniform split” and “realistic split” refer to splitting the set of compounds. We refrained from renaming these splits “uniform compound split” and “regular compound split”, to stay concise. We are happy to also change that, at the (copy) editor’s discretion. We hope that these changes improve readability.

We added a sentence when introducing the realistic split and also reworded the following paragraph, to better explain why the realistic split will result in realistic results, i.e., results similar to what we will see in practice.

9. Paragraph, “Finally, we performed ablation studies..”. Please add a sentence or two summary to explain the results (instead of having to look into the supplementary).

The paragraph already contains the important results from the tables: “In all three cases, we observe only a slight drop in performance. Notably, even with the ‘maximum challenge’ evaluation the 2-step method outperformed the pretrain/fine-tune methods for the realistic

split. [...] Notably, as few as 15 anchors are sufficient to establish the mapping." We have moved the reference to the supplementary tables to the end of the paragraph, to make clear that no massive surprises (beyond the surprisingly small drop in performance) are to be found there.

10. Why not use grid search for Ranknet?

For our GNN-based model, we perform grid search on GNN embedding size, the number of message-passing steps, and the dimensions of the two parts of feed-forward layers. The first two GNN-related hyperparameters do not exist for the RankNet, and the first part of feed-forward layers is part of encoding chromatographic conditions, likewise not applicable to RankNet. Consequently, we performed a "grid search" for the last remaining hyperparameter for RankNet, but decided against introducing additional hyperparameters for this architecture. We clarified that the three configurations tested for the RankNet correspond to the relevant parameters of the grid search.

We want to stress that this is not relevant; the RankNet architecture showed a small yet consistent decreased performance in our evaluation setup, and we doubt that any hyperparameter magic can change that. But if somebody makes it work, great! We will be happy to use RankNet instead of a GNN in the future.

11. Fixing the number of training epochs: Why not stop on validation loss as is standard in practice. Even some models can overfit with 10 epochs.

We previously touched on this in the answer to the second reviewer in the first revision. Yet, this point was not made sufficiently clear in the manuscript. We therefore added a new Supplementary Figure (24) with details on the choice of ten epochs to counteract overfitting. In short, unless we leave out complete datasets for validation, we do not find signs of overfitting on validation samples (i.e., compound pairs from training datasets) where the validation loss keeps decreasing while error on hold-out datasets already increases. We conjecture that the overall performance drop of leaving out a sufficient number of datasets only to determine the optimal number of epochs is not worth it. All our evaluations are based on hold-out datasets instead, demonstrating that overfitting is not a substantial problem and the ROI prediction model does work very well for unknown datasets.

12. There is a lot of repetition on what was removed: in the void volume, doublets, compound pair, etc.. Search for these key words and see if you can consolidate the description please to reduce redundancy.

This confusing "repetition" is due to the two parts of the manuscript: One is the 2-step method and its evaluation, the second part is the statistics on what chromatographic parameters are relevant for retention order change. Unfortunately, we indeed need different datasets for evaluation vs. statistics: For the evaluation we discard anything that is clearly dubious (you do not want to judge and compare the methods' performance based on dubious data) whereas for the statistics, we keep as much as possible (you do not want to do statistics on data when you have previously removed a lot of that data, basically using the same statistics).

We agree that this situation is far from perfect. The simplest and cleanest solution might be to cut out the complete statistics part of the paper.

13. The evaluation is sometimes reported as accuracy and sometimes as error rate. Is there a method or pattern? Please explain or use the same metric.

For all main evaluations, we use the error rate, as accuracies often reach beyond 90%. We use accuracy only for evaluations on setup-characteristic compound pairs as part of our ablation study. We added explanations for this distinction in Results and Methods.

14. Not clear how the "Measuring MS/MS libraries" section is substantially different from "Deconvolution of chimeric spectra". Please re-write to clarify, and if related combine.

Construction of MS/MS libraries is usually based on measuring pools of substances; doing so saves time compared to single-compound injections. For that, mixtures of reference standards of non-isomeric and non-isobaric substances are prepared. Unfortunately, contaminants, unforeseen overlap of adduct m/z values, and other factors can hamper the unambiguous assignment of a compound to a peak. RT prediction can help to identify the correct peak or to evaluate beyond mass for the experimental design (generations of mixtures), which compounds can safely be pooled together.

For deconvoluting chimeric spectra, this is merely an observation: Unfortunately, some compounds eluted at the same time, and resulted in chimeric spectra. Here, RT prediction can help when deconvoluting the actual experimental spectral.

We added a sentence to "Measuring MS/MS libraries" to clarify that these are indeed two very different applications.

15. Supplementary Note 2 seems redundant, and its tone is inappropriate for publication. Please remove or re-write, or potentially add in a table format for emphasizing the point, if not covered in the paper?

We were asked repeatedly and explicitly, by both the editor and reviewers, **to describe the advantages of our 2-step method over the pretrain/fine-tune paradigm**. We collected the advantages in SNote 2, and referenced this note in the results section. We are not sure in what form the tone is inappropriate for publication. This note is merely stating the facts of what is required to use a pretrain/fine-tune method in practice. We assume that everybody who has used a pretrain/fine-tune method, would describe it in a similar fashion. If this is not the case, we are happy to change that.

16. Supplementary Figure 18 is confusing. You are assuming you are not doing library search first? Why does the reader need to see candidates 3, 4, and 9? Maybe re-write to clarify what step is happening first, and how the cosine similarities from the library are being used. There is no clear methodology on how to combine SIRIUS or other annotation techniques and retention time.

The naive way to combine those is the same that is used for "in-house spectral library search": Define some threshold of what retention time difference you are still accepting as valid, and discard any candidates that are beyond this threshold. This may not be the smartest way to go for, and we would much prefer not to open this discussion in the current manuscript, because it is clearly beyond its topic. Yet, it is without doubt that one can combine retention times and MS/MS for improved structure annotation.

For our example application, we performed both spectral library search and in-silico structure database search (this is combined in SIRIUS), to showcase that retention time filtering can help in both cases. For the best spectral library match (candidate 9) the retention time does not match; same for the best in-silico structure candidate (position 1). With transferable retention time prediction, we were able to filter out these candidates, leaving one highly-ranked candidate with a good spectrum and retention time match (candidate 2). The remaining candidates are shown because they are structurally very similar to the first two, yet have notably different retention time predictions. We described this in more detail both in the figure and the figure caption. Regarding cosine similarity, we clarified that this is shown in percent in the SIRIUS interface and thus the figure, perhaps this was confusing. We also now highlight more which of the candidates have a spectrum and retention time match.

17. Please use newer references for de novo generation.

Done. The field of de novo structure generation from mass spectra is currently "exploding", so we added a selection of newer references, which we can expand at the discretion of the editor.

18. Supplementary Figure 9 panels are not provided. Not clear what the peaks are showing

Fixed this, and added short descriptions for the panels, also directly in the figure.

19. Add a limitation that it is not clear how to incorporate retention time prediction with generation or annotation. Lots of research to be done!

We would rather call that "future work" than a limitation; we have added a sentence to the conclusion (end of 2nd paragraph)

- P. 33, What does this mean, "pretrain/fine-tune methods solely for readability"?

We have changed that to, "Notably, methods without pretraining suffer from the same issues, but perform substantially worse than pretrain/fine-tune methods in practice."

Reviewer #2 (Remarks to the Author):

Firstly, apologies for the slow re-review of the paper, it arrived just before a month of holiday and conferencing! Thank you for the update to the paper. Generally, I found the paper to me much more readable with the only stylistic issues being those enforced by the journal! It now reads very well and I did not need to read the paper multiple times to understand what is going on and what the advantages are of the methods over existing similar sounds deep learning methods.

I think its now really clearly demonstrated what the advantages are off the methods. Namely, that by incorporating the chromatographic settings into the GNN, you are able to generalise to new setup. This is achieved by combining these chromatographic settings with reference annotations that can be made using any sample, rather than requiring a large number of experiments to gather reference data. The result is that this work is important for metabolite identifications on any new LC setup or after changes to an existing setup. Based on this, I think this method does offer a valuable contribution to the community and would be used by many people to help with their identifications. I would therefore be happy to see the work published in this journal.

We thank the reviewer for the positive evaluation. We share the view that this will be a valuable contribution to the community and improve metabolite identification rates, confidence, and lead to exciting new metabolomics insights.

One final comment for future consideration. In the future you might still want to consider more epochs if you can. There is a phenomenon in deep learning called double descent where you initially see a graph like the final one in your reviewer response and then eventually you see a second decent in the test error rate. I am not saying that is necessarily happening here, just something to consider looking at.

Our observations are indeed somewhat counterintuitive or, more precisely, different from what other people have observed in ML. See also the answer to reviewer #1. Potentially, more epochs will become beneficial as soon as we have more training data and move to a transformer-based architecture.

Reviewer #3 (Remarks to the Author):

I understand that the authors have already rewritten the manuscript entirely for the revised version, but it still remains inadequate. The text stays wordy, has a weak structure, often includes unnecessary details, introduces "new" problems and concepts too early, fails to motivate what's coming next or to conclude sections, and switches between design and benchmarking in a confusing way. More effort is required to streamline the manuscript.

We are happy to change any individual suggestions, and also to incorporate changes by the editor and copyeditor. What does “inadequate”, “wordy”, “weak structure”, “unnecessary details”, “introduces too early”, “fail to motivate” etc. actually mean? We are very, very puzzled.

By admission of the authors, "The manuscript is too long." Large portions of the statistical analysis and the final part on the influence of LC conditions on order can be shortened or moved to the supplement.

We are happy to do so, at the discretion of the editor. We cannot move much more of the second part (statistics) to the supplement, since there are less than 2 pages remaining. A possible solution would be to cut out the statistical part from the manuscript.

The analysis of factors affecting order and time is, in fact, of little interest. The main conclusion is that "name, column, mobile phase, gradient, temperature, and flow rate" influence retention order. Isn't this obvious?

No, it is definitely not. Before this manuscript, it was unknown. We did not know it, either, when we started the project. For the “little interest” comment: A possible solution would be to cut out the statistical part from the manuscript, see above.

The abstract needs revision. Rewrite the first four sentences to clearly state what was accomplished. Avoid phrases like "Our goal is...", "Our method should...". This section is a summary of results, not an introduction.

We are again puzzled, and will be happy to discuss with the editor and copyeditor on how we should best state the topic of this manuscript. This is not the first paper we write, and we believe that the abstract is rather crisp.

Avoid vague statements such as "Unfortunately, retention times may change massively, even for nominally identical chromatographic conditions," especially if you already demonstrate the opposite - or clarify what "nominally" means.

This appears to be a misunderstanding, see SFig. 7. **Retention times do vary massively.** We have added a sentence why retention order is better conserved than retention times.

The results section must be precise: the new method does NOT outperform DeepGCN-RT, except when focusing on compounds that are very structurally different.

We disagree. The sentence does not say, “our method outperforms all other methods for all splits on all datasets that you can ever come up with.” This appears to be unnecessary fighting over words, and we are happy to discuss all details with the editor and copyeditor.

Yet, to make that clear: We are considering two splits in our manuscript. First, a uniform split, which is rightfully "banned" from publications on bioactivity and protein binding etc., because it is of extremely limited value for assessing a model's performance in real life. There, DeepGCN-RT and our method perform basically *on par*. The second is a realistic split, that shows the performance of the models in the desired application domain: For metabolomics and related fields, this includes all molecules of biological interest, i.e. everything we would expect to see in a biological sample. There, **our method clearly outperforms DeepGCN-RT**. In combination, the sentence appears valid to us.

The last sentence is of limited relevance and should be replaced with a statement on broader impact and applications.

The last sentence is required for the second part of our manuscript, i.e. statistics. See above.

The introduction could also be shortened by combining the last paragraph with the first. The paragraph on retention time indexes could instead be moved later in the results section, when describing the 2-step approach.

We are happy to discuss these details with the editor and copyeditor.

2) Novelty
The novelty of the new method is sufficiently clear in the revision. (i) The architecture with the split (structure + LC conditions -> ROI, and ROI + anchors -> RT prediction) is novel, (ii) they are the first to demonstrate transferable predictions.

Thank you. Precisely speaking, 2-step is the first method that **can do** transferable retention time predictions. No existing method can.

3) Impact and wide applicability
By designing a transferable prediction system, the authors established a foundation that ensures broad applicability. Any scientist can predict elution times in their own reversed-phase LC setup. However, I have several concerns about the impact. The main reason is that the authors have focused on demonstrating performance, but little has been done to illustrate the impact.

Following the suggestions of the reviewers from the previous revision, we have added a detailed paragraph at the start of the results section, plus a detailed supplementary note that describes the impact and applications of our method. In addition, we have added a demonstrator analysis of a biological (fecal) dataset. The main impact is not that everybody can now predict elution times for their own chromatographic setup; the main impact is that you can do so without first measuring hundreds of authentic standards on your setup.

For example, the results (Figure 4) show that the new method is comparable to the state-of-the-art model trained on the training data (DeepGCN-RT), but the new method generalises better when tested on a subset of molecules that differ from the training set ("realistic split"). This is important from a theoretical perspective, but not a decisive argument. Are there good examples to demonstrate that better transfer leads to improved annotation?

Again, the most important difference is that our method was not trained on the evaluation (training) dataset. **Pretrain/fine-tune methods cannot do transferable retention time prediction.** The fact that our method achieves better performance than the previous best-in-class method is, in comparison, almost of secondary importance.

This is demonstrated for the biological (fecal) dataset, where the only known molecules measured on the chromatographic setup were a set of NAPS molecules not found in the biological samples. **No pretrain/fine-tune models can be fine-tuned using only 19 NAPS molecules.** We have added a description of what transferable prediction means to the results section, and we have extended the corresponding paragraph to better explain the impact. We have added a description to the results section, explaining why it is impossible to use pretrain/fine-tune methods for the analysis of the fecal dataset. Also, we have clarified that pretrain/fine-tune methods cannot do transferable retention time predictions.

Measuring an arbitrary set of reference molecules to be used as anchors, or not measuring them at all (high-confidence spectral library hits), frees the user from the need to have potentially expensive chemicals showing similarity to the targets of interest, and instead use cheap and readily available substances. We have added this to Supplementary Note 2.

In my view, the impact of improved RT prediction should be assessed by its ability to disambiguate isomers in LC feature annotation, which could involve known compounds that are structurally similar to others in the sample. I assume this is what the analysis of the faecal sample dataset aimed to show (Figure S17), but this part is buried at the end of the methods, not connected or referenced elsewhere in the paper, and lacking figures in the main text or citations in the manuscript, abstract, or conclusion. I wondered whether this is a remnant from an earlier version of the manuscript. Perhaps it's because the two cholate examples shown are not particularly impressive, as they could be distinguished by different LC methods, and probably also by Deepgcn-RT. In any case, analyzing the impact of the new method on isomer annotation is crucial to demonstrate the broader significance of the improved RT prediction.

Differentiating between isomers and improving annotations is just one of the ten applications mentioned in SNote 1. Also, we present an example of exactly this question for the fecal dataset (SFigs 17 & 18). Again, pretrain/fine-tune methods such as DeepGCN-RT cannot be applied for the fecal dataset example, because there are no data for fine-tuning.

Importantly, the fecal dataset "evaluation" is anecdotal. We are aware that chemists and biochemists often prefer such anecdotal evaluations, and this has resulted in a large number of published computational methods that work basically for one or two datasets, and never again after that. But this is a computational methods paper and our evaluations must follow the standards of the field. We have clearly demonstrated that our method works for hundreds of datasets, using a thorough and statistically sound evaluation.

Doing a full statistical evaluation on how 2-step will improve compound annotation is beyond the scope of this manuscript, would take many months to execute, and would definitely merit a paper of its own. Again, demonstrating this is not required to see that transferable retention time prediction is important.

Finally, there is also the limitation of the existing method to reversed-phase LC, which is adequate for moderately polar to nonpolar compounds, including drugs, but not suitable for

polar metabolites that differ by the position or orientation of a polar group. This limitation is discussed in the conclusion, highlighting HILIC as the next major challenge. My impression is that, regardless of how sophisticated and elegant the new method already is, its impact remains limited until transferable predictions and impact are demonstrated for non-reversed-phase LC.

Reversed-phase separation currently represents the most widely used chromatographic mode in metabolomics (see Harrieder et al., 2022), constituting about 75% of all datasets in public repositories. Transferable retention time prediction for "only" RP therefore will be immediately useful for the vast majority of experiments. Indeed, RP is not well-suited for the analysis of polar metabolites, and HILIC represents a suitable alternative. However, in contrast to RP, even more diverse column chemistries exist for HILIC, and the interaction between the metabolite, the stationary and mobile phase is even more complex, and secondary interactions such as electrostatic interaction, dipole interaction, etc. play an even more important role than for RP. HILIC is not simply the reversal of the elution order of RP. Furthermore, no system exists to describe column selectivities, similar to the HSM and Tanaka parameters for RP. Without this, transferable retention time prediction for HILIC may be impossible to achieve, let alone an integrated method for both RP and HILIC. But just like nobody would ask for a computational method that can analyze both GC and LC data, asking for one method that works both for RP and HILIC may simply not be expedient.

Minor points

- It would be helpful for the broader readership if Figure 1A presented the overall architecture, highlighting what step 1 and step 2 are, and where and what experimental data flow into (what is now divided across 1e-g). Details on the architecture or training of the ROI should be moved to 1B etc.

- Figure 3: What data are shown? Are these 3 studies from MetaboLights?

Sorry, this was missing from the caption; we added the IDs for these *RepoRT* datasets.

- Figure 4 and Table 2: What is the unit? Is it seconds or delta(normalized retention time)?

As written in the first sentences of the respective captions, these are normalized retention times, given in percentage points with the gradient length representing 100 (detailed in the manuscript at the first usage of this term).

- Supp Figure 17: Check the molecular structure shown in panel g: if I remember correctly, the methyl group in the fatty acyl chain is in the opposite direction.

Thank you for catching this, fixed. We had previously used the automatic representation from RDKit.
